# Traditional Chinese medicine injections with Tonifying Qi, equivalent effect of regulating energy metabolism, for acute myocardial infarction: a systematic review and meta-analysis of randomized clinical trials

**DOI:** 10.3389/fphar.2025.1511486

**Published:** 2025-03-28

**Authors:** Huiwen Zhou, Jiaping Chen, Hongxu Liu, Xiang Li, Huiqi Zong, Shuwen Zhang, Yuxin Shi, Yunze Li

**Affiliations:** ^1^ Department of Cardiology, Beijing Hospital of Traditional Chinese Medicine, Capital Medical University, Beijing, China; ^2^ Department of Cardiology, Linping Branch of Hangzhou Hospital of Traditional Chinese Medicine, Hangzhou, Zhejiang, China; ^3^ Department of Cardiology, Lhasa People’s Hospital, Lhasa, China

**Keywords:** Chinese medicine injections, acute myocardial infarction, meta-analysis, traditional Chinese medicine, energy metabolism

## Abstract

**Background:**

Traditional Chinese medicine injections for Tonifying Qi (TCMi-TQs), which exhibits comparable effect of regulating energy metabolism, is commonly used as an adjuvant treatment for acute myocardial infarction (AMI) in China.

**Objective:**

A systematic review and meta-analysis was conducted to contrast the effectiveness and safety of four TCMi-TQs in AMI.

**Methods:**

Eight Databases were thoroughly searched before 31 July 2024, for randomized controlled trials (RCTs) focusing on the application of TCMi-TQs combined with conventional treatments (CT) to treat AMI. The primary outcomes were in-hospital mortality and long-term mortality. Secondary outcomes included malignant arrhythmia, left ventricular ejection fraction (LVEF), and adverse events. Stata17.0 and RevMan 5.4.1 software were employed for meta-analysis. The quality of evidence was evaluated using the GRADE approach.

**Results:**

A total of 113 RCTs involving 10,779 patients were included in the analysis, none of which described in-dependent testing of the purity or potency of the TCMi-TQ product used. 51/113 reported random sequence generation. All RCTs lack adequate description of allocation concealment. 112/113 failed to assess blinding. The meta-analysis results demonstrated that the combined application of TCMi-TQ + CT, compared with CT, significantly reduced in-hospital mortality in AMI patients [RR = 0.58, 95% CI (0.51, 0.67), *P* < 0.05], decreased the incidence of malignant arrhythmia [RR = 0.51, 95%CI(0.42, 0.63), *P* < 0.05], increased LVEF [MD = 6.52, 95%CI(5.54, 7.50), *P* < 0.05], and decreased the incidence of adverse events [RR = 0.70, 95%CI(0.60, 0.81), *P* < 0.05]. The GRADE evidence quality classification indicated that the evidence for in-hospital mortality, malignant arrhythmia, and adverse events was of moderate quality, while the evidence for LVEF was of low quality.

**Conclusion:**

TCMi-TQ demonstrates additional clinical value in reducing mortality, the risk of malignant arrhythmia, and adverse events in patients with AMI. However, further validation of these findings is warranted through high-quality clinical trials due to methodological weaknesses in randomization, blinding, allocation concealment, and insufficient assessment of the purity/potency of botanical drugs and the quantity of active metabolites.

**Systematic Review Registration:**

https://www.crd.york.ac.uk/PROSPERO/view/CRD42024573818, identifier PROSPERO (CRD42024573818).

## 1 Introduction

Acute myocardial infarction (AMI) is a significant global health issue characterized by high morbidity and mortality rates, imposing substantial economic and medical burdens (Mensah et al., 2023). AMI is usually caused by coronary artery disease, and research has found that AMI can be associated with cerebrovascular diseases, making clinical diagnosis and treatment more difficult due to comorbidities of the heart and brain (Suzuki et al., 2023). Over the past decade, the management of AMI in China has made some progress. However, the *China Cardiovascular Health and Disease Report 2023 Summary* revealed an increase in AMI mortality in China from 2002 to 2021 (National Center Cardiovascular Diseases, 2024). With the active promotion of secondary prevention measures for coronary heart disease and early reperfusion therapy for AMI, the mortality rate of AMI patients has decreased (Roger et al., 2010). There are still several unresolved issues after reperfusion, including decreased myocardial contractility, ventricular arrhythmia, and no-reflow phenomenon (Thiele et al., 2017). These complications have a significant impact on the prognosis of patients (Heusch and Gersh, 2017). Therefore, exploring additional effective treatment methods remains essential.

Traditional Chinese medicine (TCM) can play a unique role in improving the clinical prognosis of AMI. Research has found that for STEMI patients, on the basis of standardized biomedicine treatment (including reperfusion therapy and optimal drug therapy), Tongxinluo can significantly improve clinical prognosis, and reduce the risk of major adverse cardiovascular and cerebrovascular events at 30°days and 1°year (Yang et al., 2023). Consequently, there is a growing interest among Chinese medical professionals in exploring therapeutic approaches from TCM to help reduce AMI mortality. This research direction aligns with the principles exemplified by Professor Tu Youyou, the Nobel Prize laureate who successfully extracted artemisinin from Artemisia annua, thereby revolutionizing malaria treatment.

One such intervention gaining attention is the use of traditional Chinese medicine injections for tonifying qi (TCMi-TQs), which possess comparable effects of regulating energy metabolism (Li et al., 2023; Wang et al., 2022; Wang A. et al., 2024; Yang et al., 2022). TCMi-TQs have shown promise in reducing mortality and the incidence of re-infarction among AMI patients (Jia et al., 2023; Lu et al., 2018). To obtain high-quality evidence regarding the safety and efficacy of TCMi-TQs in AMI, this study initiated a search for TCMi-TQs used in the treatment of AMI. The search yielded four TCMi-TQs: Shengmai injection (SGMI), Shenmai injection (SMI), Shenfu injection (SFI), and Astragalus injection (AI). Research has demonstrated that these four TCMi-TQs and their main active metabolites play a significant role in modulating myocardial energy metabolism in patients with myocardial ischemia. SGMI increases the number of myocardial cell mitochondria and scavenges oxygen-free radicals (Lu et al., 2005). SMI enhances myocardial microcirculation parameters (Yang and Wang, 2021), while SFI mitigates mitochondrial oxidative stress (Lu and Xiang, 2023). The primary active compound of AI, astragaloside IV, regulates myocardial cell oxidative stress and enhances mitochondrial function (Guan et al., 2023). Detailed information on these four TCMi-TQs is provided in Supplementary Tables S1–S4. The objective of this study is to systematically collect and analyze the data from these four randomized controlled trials (RCTs) investigating TCMi-TQs in the treatment of AMI, with the aim of evaluating its efficacy and safety for AMI patients presenting with relevant indications based on the current evidence. This study has the potential to bridge the gap between TCMi-TQs and modern medicine for AMI treatment and open up avenues for integrative care models for AMI patients.

## 2 Methods

The systematic review has been registered in the PROSPERO platform for prospective registration with the registration number CRD42024573818. The Preferred Reporting Items for Systematic Reviews and Meta-analyses (PRISMA) guidelines (Moher et al., 2015; Page et al., 2021) were employed to conduct our network meta-analysis, as seen in Supplementary Material. To ensure accurate reporting of four TCMIs in this analysis, we adhered to the guidelines established in the consensus statement on the Phytochemical Characterization of Medicinal Plant extract (ConPhyMP) (Supplementary Tables S1–S4) (Heinrich et al., 2022).

### 2.1 Inclusion and exclusion criteria

The inclusion criteria for this review are as follows:

(1) Study Type: RCTs.
(2) Study Subjects: Patients who meet the diagnostic criteria for AMI.
(3) Type of Intervention: The observation group received any one of the traditional Chinese medicine injections with TCMi-TQ interventions, including SGMI, SMI, SFI, or AI, in addition to conventional treatment. The control group received conventional treatment (CT), which included general treatment (monitoring vital signs, symptom relief, *etc.*), reperfusion therapy (Percutaneous Coronary Intervention (PCI), thrombolysis, and coronary artery bypass surgery), and pharmacotherapy (antiplatelet agents, anticoagulants, lipid-lowering drugs, *etc.*), while excluding commercial Chinese polyherbal preparation (CCPP), acupuncture, and other traditional medical treatments. The sole difference between the two groups was the administration of TCMi-TQ.
(4) Outcome Measures: The primary outcome measures were in-hospital mortality and long-term mortality. Long-term mortality was defined as mortality occurring at least 1 year after the onset of AMI. Secondary outcomes included the incidence of malignant arrhythmias affecting hemodynamics (such as ventricular fibrillation, polymorphic ventricular tachycardia, and second or third-degree atrioventricular block with hemodynamic disturbance), changes in left ventricular ejection fraction (LVEF) before and after treatment, and adverse safety events (such as dizziness, nausea, and allergic reactions).

The exclusion criteria for this review are as follows:

(1) No mention of diagnostic criteria in the literature or unclear diagnostic criteria.
(2) Control settings of clinical trials that were unreasonable or did not meet the inclusion criteria for this study, such as the inclusion of other CCPP in the experimental group, were excluded.
(3) Duplicate published literature.
(4) Studies that did not include the required effect measures.

### 2.2 Literature resources

We conducted a comprehensive literature search using multiple databases, including PubMed, EMBASE, Scopus, Cochrane Central Register of Controlled Trials, and Web of Science. Additionally, we searched the China Biological Literature Database, China National Knowledge Infrastructure, VIP database, and Wanfang Data Knowledge Service Platform. The search covered the period from the establishment of each database until July 2024. The search strategy is provided in Supplementary Table S5. In addition to electronic database searches, we also examined the reference lists of relevant articles and manually searched printed books and magazines in the field to ensure a comprehensive literature review. To identify relevant clinical trials, we also searched for registered trials on ClinicalTrials.gov to identify any unpublished articles that met our inclusion criteria.

### 2.3 Literature screening, information extraction and quality assessment

Two reviewers (YX Shi and YZ Li) independently performed each step according to established search rules. The screening process involved reviewing the title and abstract of each retrieved article and applying predefined inclusion and exclusion criteria. Irrelevant articles were excluded, and no discussion took place until the final results were summarized.

After retrieving the articles, the two reviewers (YX Shi and YZ Li) independently processed the data, identified and removed duplicate articles, retained the most recent publications with the most complete data, excluded articles that did not meet the inclusion or exclusion criteria, and documented the reasons for exclusion. The extracted data included the article title, all authors, year of publication, journal, sample size, participant characteristics, treatment interventions, blinding methods, randomization procedures, outcome measures, adverse events, and other relevant information, which were summarized in a table. In case of any disagreements, a third reviewer (HQ Zong) made the final judgment and resolved the discrepancies.

Risk of bias was assessed by two reviewers (YX Shi and YZ Li) independently, using the Cochrane risk of bias tool (RoB 2.0 Tool) (Sterne et al., 2019). Overall quality of evidence was rated using the Grades of Rec ommendations, Assessment, Development and Evaluation (GRADE) approach (Chen et al., 2018).

Any disagreement between the 2 reviewers (YX Shi and YZ Li) will be resolved by a discussion. Further disagreements will be arbitrated by the third author (HQ Zong).

### 2.4 Data analysis

RevMan 5.4.1 software was utilized to analyze the extracted clinical research data. Relative risk (RR) analysis was employed for count data, while mean difference (MD) was used for measurement data when the unit of measurement was the same. Standardized mean difference (SMD) was used for measurement data when the unit of measurement differed. All effect sizes were reported with a 95% confidence interval (CI). For continuous outcomes, the change difference was employed for meta-analysis. The mean and standard deviation of the change difference before and after the intervention were calculated using the formula provided in the Cochrane handbook (Higgins et al., 2023).

$$\begin{array}{c} {SD}_{change}=\sqrt{{SD}_{pre}^{2}+{SD}_{post}^{2}-\left(2\times Corr\times {SD}_{pre}\times {SD}_{post}\right)}\\ \text{Corr}=0.8 \end{array}$$

In this study, the mortality rate, incidence of malignant arrhythmia, and incidence of adverse events were presented using RR. LVEF was presented using the mean and standard deviation of the difference before and after treatment. Heterogeneity among the included studies was assessed using the Q test. A significance level of *P* ≤ 0.10 and an I^2^ value ≥50% were used as criteria for significant heterogeneity. If the *P* value was greater than 0.1 and the I^2^ value was less than 50%, a fixed-effect model was used for statistical analysis. If the *P* value was less than or equal to 0.1 and the I^2^ value was greater than or equal to 50%, a random-effects model was applied based on sensitivity analysis (Deeks et al., 2023). Statistical significance was defined as *P* < 0.05.

In cases where heterogeneity (I^2^ > 50%) was observed among the studies, subgroup analysis and sensitivity analysis were conducted to explore the sources of heterogeneity and verify the stability of the meta-analysis results. Funnel plots were generated using RevMan 5.4.1 software, and Egger’s test was performed using Stata 17.0 software for studies with 10 or more articles to assess potential publication bias. If the *P* value of Egger’s test was less than 0.05, it indicated the presence of publication bias among the studies (Egger et al., 1997). For studies exhibiting publication bias, the trim-and-fill method was employed to adjust the results, assuming that missing studies likely occupied symmetrical positions relative to the existing ones—these being studies potentially withheld due to publication bias, such as those reporting negative results (Shi and Lin, 2019).

The evidence quality of outcome indicators was evaluated using the GRADEpro GDT online tool. The default assumption was that the evidence quality of RCTs was high (Guyatt et al., 2008). The evidence quality of outcome indicators was assessed based on five downgrade factors: risk of bias, inconsistency, indirectness, imprecision, and publication bias (Chen et al., 2018).

## 3 Results

### 3.1 Literature screening

**FIGURE 1:**
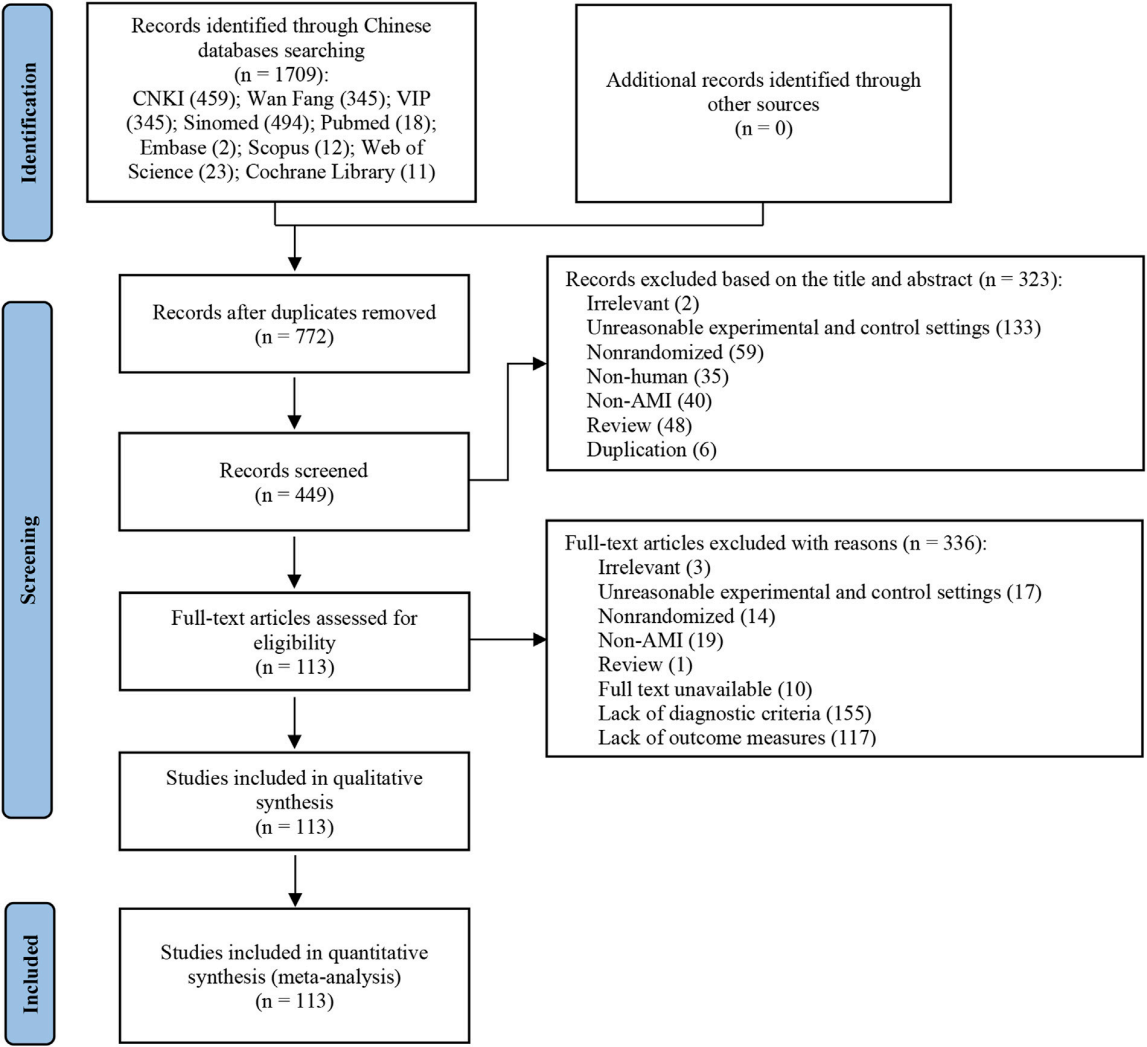
Flow chart of the study selection. The initial review yielded 1709 articles, leaving 772 articles after removing duplicates. After selection based on title or abstract, 449 articles were screened for full text review. Of these, 3 articles were irrelevant, 17 articles had unreasonable experimental and control settings, 14 articles were nonrandomized, 19 articles were non-AMI, an article was review, 10 articles’ full text were unavailable, 155 articles were lack of diagnostic criteria, 117 articles were lack of outcome measures. Finally, 113 full-text articles were included.

The process of study selection and identification is depicted in Figure 1. Initially, a total of 1709 potentially relevant articles were retrieved from electronic databases. After removing 937 duplicates, 772 articles remained for further screening. Following title and abstract screening, 323 records were excluded, leaving 449 records. Subsequently, 336 articles were excluded for the following reasons: irrelevant study (n = 3), unreasonable experimental and control settings (n = 17), Nonrandomized (n = 14), Non-AMI (n = 19), review (n = 1), full-text unavailable (n = 10), lack of diagnosis criteria (n = 155), and lack of outcome measures (n = 117). Finally, 113 full-text articles were included.

### 3.2 Study characteristics

A total of 113 studies (28–140) met the final eligibility criteria and were included in the meta-analysis (Table 1). All studies were single-center trials conducted in China. Two trial was a three-arm study (Wang, 2016; Li, 2006), while the remaining trials were two-arm studies. The sample sizes ranged from 30 (125) to 502 (139), with the mean age ranging from 35 to 75 years. The duration of treatment varied from once (Wang, 2018; Zhang D. L. et al., 2018; Zhang, 2017) to 30 days (Wang, 2016). We identified two ongoing trials that may be relevant to this review. Supplementary Table S6 provides details of the CCPP included in the study.

**TABLE 1:** Characteristics of the included studies.

Study ID	Sample size (E/C)	Sex (M/F)	Age(Y) (E/C)	Type of AMI	Reperfusion therapies	Intervention (E)	Intervention (C)	Course (days)	Follow-up	Outcomes	Adverse events	Treatment based on syndrome differentiation
Shengmai injection												
Cui and Li (2006)	38/36	42/32	56.5/54.2	ALL AMI	Thrombolytic therapy	SGMI 60 mL ivgtt Qd combined with conventional therapy,②, ⑨	Conventional therapy combined with ②, ⑨	14	NR	(1)	NR	NR
Wang et al. (2019a)	53/53	60/46	59.39 ± 10.25/59.43 ± 10.31	STEMI	NR	SGMI 60 mL ivgtt Qd combined with conventional therapy, ③, ④, ⑥, ⑧, ⑤, ⑬	Conventional therapy, ③, ④, ⑥, ⑧, ⑤, ⑬	5	NR	(4)	NR	NR
Lu and Yao (2022)	45/45	44/46	57.74 ± 7.28/58.67 ± 7.34	STEMI	NR	SGMI 60 mL ivgtt Qd combined with ⑧	⑧	15	NR	(4)	ⅣⅥ Ⅸ	NR
Xu (2022)	93/93	107/79	66.56 ± 3.35/66.69 ± 3.78	STEMI	PCI	SGMI 60 mL ivgtt Qd combined with ①, ③, ⑥, ⑩, ⑭	①, ③, ⑥, ⑩, ⑭	7	3 M	(4)	Ⅵ	NR
Chen (2017)	25/25	23/27	56.6 ± 10.2/54.9 ± 10.3	STEMI	PCI	SGMI 60 mL ivgtt Qd combined with ①, ②, ③, ⑩	①, ②, ③, ⑩	7	3 M	(1) (4)	NR	NR
Song (2018)	60/60	80/40	54.1 ± 4.6/54.8 ± 4.2	ALL AMI	Thrombolytic therapy	SGMI 40 mL ivgtt Qd combined with ⑧, ⑨	⑧, ⑨	7	NR	(4)	NR	YES
Liang (2006)	30/30	34/26	NR	STEMI	Thrombolytic therapy	SGMI 40 mL ivgtt Qd combined with ①, ②, ⑨	①, ②, ⑨	10	NR	(1)	Ⅰ	NR
Lu (2011)	34/34	44/24	53.2/54.7	STEMI	Thrombolytic therapy	SGMI 40 mL ivgtt Qd combined with ①, ②, ⑥, ⑧	①, ②, ⑥, ⑨	7 to 14	NR	NR	Ⅰ	NR
Lu (2009)	45/30	44/31	62 ± 8.5/64 ± 8.8	ALL AMI	Thrombolytic therapy	SGMI 40–60 mL ivgtt Qd combined with ①, ②, ⑥, ⑧, ⑭	①, ②, ⑥, ⑨, ⑭	10 to 14	NR	NR	Ⅰ	NR
Ding and Xu (2006)	15/15	16/14	NR	STEMI	Thrombolytic therapy	SGMI 30 mL ivgtt Q12 h combined with conventional therapy, ⑨	Conventional therapy combined with ⑨	7	NR	(1)	NR	NR
Tang (2019)	51/51	58/44	65.68 ± 3.2/65.53 ± 3.14	STEMI	Thrombolytic therapy	SGMI 20–50 mL ivgtt Qd combined with ①, ⑥, ⑧	①, ⑥, ⑧	14	NR	NR	ⅠⅨ	NR
Wang et al. (2010)	32/30	35/27	58 ± 14.9/54.9 ± 15.2	STEMI	PCI	SGMI 10 mL iv before surgery, SGMI 50 mL ivgtt Qd combined with ①, ②, ③, ④, ⑤, ⑥, ⑩	①, ②, ③, ④, ⑤, ⑥, ⑩	7	NR	(3) (4)	NR	NR
Wang (2008)	30/30	44/16	54.0 ± 14.9/54.9 ± 15.2	STEMI	PCI	SGMI 10 mL iv before surgery, SGMI 50 mL ivgtt Qd combined with ①, ②, ③, ④, ⑤, ⑥, ⑩	①, ②, ③, ④, ⑤, ⑥, ⑩	7	NR	(3)	NR	NR
Yang and Cai (2016)	98/98	101/95	57.03 ± 6.74/56.27 ± 40.31	ALL AMI	Thrombolytic therapy	SGMI 20–60 mL ivgtt Qd combined with ①, ②, ⑧, ⑨	②, ⑧, ⑨	7	NR	(1) (4)	ⅡⅦ	YES
Shenmai injection												
Wei and Liu (2001)	19/15	20/14	56 ± 2.3/56 ± 1.8	STEMI	Thrombolytic therapy	SMI 60 mL ivgtt Qd combined with Conventional therapy, ①, ②, ⑨	Conventional therapy combined with ①, ②, ⑨	14–28	NR	(1) (3)	Ⅰ	NR
Zheng (2016)	34/34	40/28	64.3 ± 4.6/66.5 ± 4.7	ALL AMI	NR	SMI 60 mL ivgtt Qd combined with conventional therapy	Conventional therapy	28	NR	(1)	NR	NR
Wang (2016)	50/50	71/29	60.73 ± 14.92/60.25 ± 14.35	ALL AMI	NR	SMI 60 mL ivgtt Qd combined with ①, ④. ⑤. ⑥. ⑧. ⑭	①, ④. ⑤. ⑥. ⑧. ⑭	30	NR	(4)	NR	NR
Guo and Zhang (1999)	243/259	355/147	64.27/65.12	ALL AMI	Thrombolytic therapy	SMI 60 mL ivgtt Qd combined with ①, ②, ⑨	①, ②, ⑨	14	NR	(1) (3)	Ⅰ	NR
Zhang (2011)	42/42	47/37	NR	ALL AMI	Thrombolytic therapy	SMI 60 mL ivgtt Qd combined with ①, ②, ⑨	①, ②, ⑨	7	NR	(1) (3)	NR	NR
Yuan (2009)	38/38	47/29	62.4 ± 12.9/62.8 ± 13.8	STEMI	Thrombolytic therapy	SMI 60 mL ivgtt Qd combined with ①, ②, ⑥, ⑨	①, ②, ⑥, ⑨	10	NR	(1) (3)	NR	NR
Yang et al. (2014)	30/30	36/24	57.77 ± 10.7/57.93 ± 10.37	AMI	PCI	SMI 5 mL iv before surgery, SMI 30 mL ivgtt Qd combined with ①, ②, ⑥, ⑩	①, ②, ⑥, ⑩	3	30D	NR	Ⅰ	YES
Zhang et al. (2019)	46/46	43/49	58.46 ± 1.99/57.69 ± 2.03	ALL AMI	NR	SMI 50 mL ivgtt Qd combined with conventional therapy	Conventional therapy	15	NR	NR	ⅣⅤ	NR
Zhao et al. (2005)	20/20	31/9	49.8 ± 11.8/50.1 ± 10.3	STEMI	Thrombolytic therapy	SMI 50 mL ivgtt Qd combined with ②, ⑨	②, ⑨	10	NR	(1)	NR	NR
Luo (2016)	46/46	54/38	58.4 ± 6.9/59.2 ± 6.5	STEMI	Thrombolytic therapy	SMI 50 mL ivgtt Qd combined with ①, ②, ⑤, ⑨, ⑮	①, ②, ⑤, ⑨, ⑮	14	NR	(4)	NR	NR
Li et al. (2016)	48/48	62/34	58.72 ± 11.63/60.19 ± 11.14	STEMI	PCI	SMI 50 mL ivgtt Qd combined with ①, ②, ④, ⑤, ⑩	①, ②, ④, ⑤, ⑩	7	3 M	(3) (4)	NR	NR
Zhao et al. (2016a)	105/105	120/90	61.8 ± 9.5/60.2 ± 10.6	STEMI	Thrombolytic therapy	SMI 50 mL ivgtt Qd combined with ①, ②, ④, ⑤, ⑨	①, ②, ④, ⑤, ⑨	14	1Y	(1) (4)	NR	NR
Guo (2014)	39/39	53/25	58.7 ± 11.7/58.7 ± 11.7	STEMI	PCI	SMI 50 mL ivgtt Qd combined with ①, ②, ③, ⑥, ⑩	①, ②, ③, ⑥, ⑩	7	3 M	(1) (4)	NR	NR
Xuan et al. (2015)	23/25	26/22	NR	ALL AMI	PCI	SMI 50 mL ivgtt Qd combined with ①, ②, ③, ⑤, ⑥, ⑩, ⑬	①, ②, ③, ⑤, ⑥, ⑩, ⑬	14	1Y	(2) (4)	NR	NR
Qu (2007)	38/30	46/22	NR	ALL AMI	Thrombolytic therapy	SMI 50 mL ivgtt Qd combined with ①, ②, ③, ⑤, ⑥, ⑨	①, ②, ③, ⑤, ⑥, ⑨	10	NR	(1) (3)	NR	NR
Wang et al. (2017)	46/46	58/34	62.72 ± 12.12/61.27 ± 10.84	STEMI	PCI	SMI 50 mL ivgtt Qd combined with ①, ②, ③, ④, ⑤, ⑩	①, ②, ③, ④, ⑤, ⑩	7	3 M	(3) (4)	NR	NR
Liu (2016)	50/50	61/39	58.41 ± 12.39/57.68 ± 12.03	STEMI	PCI	SMI 50 mL ivgtt Qd combined with ①, ②, ③, ④, ⑤, ⑥, ⑩	①, ②, ③, ④, ⑤, ⑥, ⑩	7	6 M	(3) (4)	NR	NR
Yang et al. (2017a)	38/38	46/30	35.4 ± 6.7/36.8 ± 5.4	STEMI	Thrombolytic therapy	SMI 50 mL ivgtt Qd combined with ①, ②, ③, ④, ⑤, ⑥, ⑨, ⑭, ⑮	①, ②, ③, ④, ⑤, ⑥, ⑨, ⑭, ⑮	14	NR	(4)	NR	NR
Yu et al. (2010)	22/26	28/20	NR	NSTEMI	NR	SMI 50 mL ivgtt Qd combined with ①, ②, ③, ④, ⑤, ⑥	①, ②, ③, ④, ⑤, ⑥	14	NR	NR	Ⅳ	NR
Ji et al. (2021)	44/45	56/33	53.91 ± 6.52/54.81 ± 6.79	STEMI	PCI	SMI 50 mL ivgtt five times a week combined with ①, ③, ④, ⑩	①, ③, ④, ⑩	28	NR	(4)	NR	YES
Chen (2021)	46/46	60/32	59.41 ± 7.04/58.95 ± 7.84	STEMI	Thrombolytic therapy	SMI 50–100 mL ivgtt Qd combined with conventional therapy, ⑨	Conventional therapy combined with ⑨	14	NR	(3)	NR	NR
Zhou (2024)	44/44	40/48	57.82 ± 5.88/57.74 ± 5.95	STEMI	NR	SMI 40 mL ivgtt Qd combined with conventional therapy, ①	Conventional therapy, ①	15	NR	(4)	NR	NR
Liu (2016)	44/44	51/37	NR	STEMI	NR	SMI 40 mL ivgtt Qd combined with conventional therapy	Conventional therapy	15	NR	(1) (3) (4)	NR	NR
Yan et al. (2018)	40/40	45/35	56.8 ± 8.4/55.9 ± 9.1	STEMI	Thrombolytic therapy	SMI 40 mL ivgtt Qd combined with ②, ⑨	②, ⑨	14	NR	(4)	NR	NR
Zhao and Sun (2021)	52/52	53/51	61.3 ± 9.3/60.4 ± 7.7	STEMI	PCI	SMI 40 mL ivgtt Qd combined with ①, ⑩	①, ⑩	14	NR	(4)	NR	NR
Ye (2010)	34/34	41/27	NR	ALL AMI	Thrombolytic therapy	SMI 40 mL ivgtt Qd combined with ①, ⑥, ⑨, ⑫, ⑬, ⑭	①, ⑥, ⑨, ⑫, ⑬, ⑭	NR	NR	(1) (3)	NR	NR
Zhou (2017)	75/75	95/55	60.7 ± 6.2/60.4 ± 7.3	STEMI	Thrombolytic therapy	SMI 40 mL ivgtt Qd combined with ①, ③, ④, ⑤, ⑥, ⑧, ⑨, ⑮	①, ③, ④, ⑤, ⑥, ⑧, ⑨, ⑮	NR	NR	(3) (4)	Ⅰ	NR
Qi et al. (2015b)	60/60	76/44	64.2 ± 2.3/62.4 ± 4.5	STEMI	Thrombolytic therapy	SMI 40 mL ivgtt Qd combined with ①, ②, ⑨	①, ②, ⑨	14	NR	(4)	NR	YES
Wu et al. (2022)	37/37	43/31	63.55 ± 4.59/63.67 ± 4.33	ALL AMI	Thrombolytic therapy	SMI 40 mL ivgtt Qd combined with ①, ②, ⑨	①, ②, ⑨	14	3 M	(4)	NR	NR
Du (2017)	44/44	47/41	59.71 ± 6.29/59.64 ± 6.38	STEMI	Thrombolytic therapy	SMI 40 mL ivgtt Qd combined with ①, ②, ⑤, ⑥, ⑨	①, ②, ⑨	14	NR	(3) (4)	NR	YES
Qi et al. (2015a)	60/60	67/53	56.4 ± 13.8/58.7 ± 14.2	STEMI	Thrombolytic therapy	SMI 40 mL ivgtt Qd combined with ①, ②, ④, ⑤, ⑨, ⑮	①, ②, ④, ⑤, ⑨, ⑮	14	NR	(4)	NR	NR
Qi et al. (2015a)	60/60	76/44	60.2 ± 13.8/61.5 ± 12.5	STEMI	Thrombolytic therapy	SMI 40 mL ivgtt Qd combined with ①, ②, ④, ⑤, ⑧, ⑮	①, ②, ④, ⑤, ⑨, ⑮	14	NR	(1)	NR	NR
Wu et al. (2016)	60/60	NR	NR	STEMI	Thrombolytic therapy	SMI 40 mL ivgtt Qd combined with ①, ②, ④, ⑤, ⑧, ⑨	①, ②, ④, ⑤, ⑧, ⑨	14	NR	(1) (3)	NR	NR
Wang et al. (2021a)	50/51	54/47	60.42 ± 12.39/61.27 ± 11.44	STEMI	NR	SMI 40 mL ivgtt Qd combined with ①	①	15	NR	(4)	ⅣⅥ	NR
Wang et al. (2019b)	41/41	49/33	62.14 ± 3.58/61.94 ± 3.75	STEMI	Thrombolytic therapy	SMI 40 mL iv, SMI 150 mL ivgtt Qd combined with ①, ②, ⑨, ⑭	①, ②, ⑨, ⑭	14	NR	(4)	NR	NR
Xie (2018)	42/42	44/40	61.54 ± 8.73/60.85 ± 8.01	ALL AMI	Thrombolytic therapy	SMI 40 mL iv, SMI 100 mL ivgtt Qd combined with ①, ⑨, ⑭	①, ⑨, ⑭	14	NR	(4)	Ⅵ	NR
Zong et al. (2014)	34/34	34/34	59.4 ± 9.2/60.5 ± 9.4	STEMI	Thrombolytic therapy	SMI 40 mL iv, SMI 100 mL ivgtt Qd combined with ①, ②, ⑨, ⑭	①, ②, ⑨, ⑭	14	NR	(1)	NR	NR
Wang et al. (2017)	35/35	48/22	58.18/58.31	STEMI	Thrombolytic therapy	SMI 40 mL iv, SMI 100 mL ivgtt Bid combined with conventional therapy, ①, ⑨	Conventional therapy, ①, ⑨	15	NR	(4)	NR	NR
Zou (2014)	31/31	40/22	57.69 ± 12.47/56.78 ± 11.63	ALL AMI	Thrombolytic therapy	SMI 40 mL iv for 3min (the first dose) and SMI 100 mL ivgtt (the maintenance dose) Qd combined with ②, ③, ④, ⑤, ⑨, ⑮	②, ③, ④, ⑤, ⑨, ⑮	15	NR	(1)	NR	NR
Liu et al. (2004)	41/94	104/33	NR	ALL AMI	Thrombolytic therapy	SMI 40–60 mL ivgtt Qd combined with ②, ⑨	②, ⑨	10–15	NR	(1) (3)	NR	NR
Yu et al. (2021)	49/49	57/41	59.03 ± 4.38/58.96 ± 4.35	STEMI	NR	SMI 3 mg/kg iv, SMI 150 mL ivgtt Qd combined with conventional therapy, ④, ⑤	Conventional therapy, ④, ⑤	14	NR	NR	ⅣⅥ	NR
Huang et al. (2015)	74/74	83/65	NR	ALL AMI	NR	SMI 3 mg/kg iv combined with conventional therapy, ③. If the effect was not satisfied after 30 min, an additional 150 mg iv could be given, followed by 0.5–1 mg/min ivgtt to maintain	Conventional therapy combined with ③	28	6 M	(1)	NR	NR
Han et al. (2003)	21/18	25/14	58/58	STEMI	Thrombolytic therapy	SMI 30 mL ivgtt Qd combined with ①, ②, ⑤, ⑥, ⑨	①, ②, ⑤, ⑥, ⑨	14	NR	(1)	Ⅰ	NR
Shi and Li (2016)	37/35	44/28	61.16 ± 6.51/60.85 ± 6.39	STEMI	NR	SMI 20 mL ivgtt Qd combined with conventional therapy, ⑨	Conventional therapy	14	NR	(4)	ⅢⅣⅥ	NR
Zhang (2017)	61/61	81/41	68.25 ± 2.1/67.74 ± 2.2	STEMI	Thrombolytic therapy	SMI 20 mL ivgtt once combined with conventional therapy, ⑨	Conventional therapy, ⑨	1	NR	(4)	NR	NR
He et al. (2016)	60/60	65/55	61.34 ± 4.21/62.16 ± 4.14	STEMI	PCI	SMI 10 mL was infused intracoronary, SMI 100 mL ivgtt Qd/Bid combined with ①, ②, ⑩	①, ②, ⑩	7–14	6 M	(1) (4)	NR	NR
Bai et al. (2002)	62/60	88/34	64.5/61.55	STEMI	Thrombolytic therapy	SMI 100 mL ivgtt Qd/Bid combined with ①, ②, ④, ⑤, ⑥, ⑨	①, ②, ④, ⑤, ⑥, ⑨	10–14	NR	(1)	NR	NR
Zhan and Cui (2023)	34/34	40/28	57.32 ± 5.57/57.23 ± 5.43	STEMI	PCI	SMI 100 mL ivgtt Qd combined with conventional therapy, ①, ⑩	Conventional therapy combined with ①, ⑩	14	NR	(4)	Ⅱ	NR
Sun (2019)	35/35	43/27	57.21 ± 7.93/57.79 ± 8.41	ALL AMI	NR	SMI 100 mL ivgtt Qd combined with conventional therapy	Conventional therapy	14	NR	(4)	Ⅵ	NR
Wang et al. (2019c)	32/32	35/29	52.18 ± 7.55/52.84 ± 7.63	ALL AMI	NR	SMI 100 mL ivgtt Qd combined with conventional therapy	Conventional therapy	7	NR	(4)	NR	NR
Yan et al. (2018)	49/49	57/41	63.27 ± 12.46/63.78 ± 12.32	ALL AMI	Thrombolytic therapy	SMI 100 mL ivgtt Qd combined with ⑨	⑨	15	6 M	(4)	NR	NR
Long (2007)	32/32	37/27	64.9 ± 3.96/63.89 ± 5.81	STEMI	Thrombolytic therapy	SMI 100 mL ivgtt Qd combined with ②, ⑥, ⑨	①, ⑤, ⑥, ⑨, ⑬	14	NR	(4)	Ⅳ	NR
Zhang et al. (2018b)	65/65	76/54	62 ± 5/63 ± 7	STEMI	Thrombolytic therapy	SMI 100 mL ivgtt Qd combined with ①, ②, ⑧, ⑨	①, ②, ⑧, ⑨	3	30 d	(1) (4)	ⅠⅡⅦ	YES
Shi et al. (2018)	56/56	87/25	61.6 ± 7.2/60.5 ± 5	STEMI	Thrombolytic therapy	SMI 100 mL ivgtt Qd combined with ①, ②, ③, ⑨	①, ②, ③, ⑨	5	NR	(4)	ⅠⅤⅥⅨ	NR
Liu (2012)	20/20	25/15	58.2 ± 5.6/57.9 ± 8.2	STEMI	Thrombolytic therapy	SMI 100 mL ivgtt Qd combined with ①, ②, ③, ⑤, ⑧, ⑨	①, ②, ③, ⑤, ⑧, ⑨	14	NR	(1)	NR	NR
Xu (2018)	34/35	39/30	62.51 ± 12.37/62.43 ± 12.85	ALL AMI	PCI	SMI 100 mL ivgtt Qd combined with ①, ②, ③, ④, ⑤, ⑩	①, ②, ③, ④, ⑤, ⑩	15	NR	(4)	NR	NR
Zhao (2020)	40/40	36/44	60 ± 4/60 ± 4	ALL AMI	PCI	SMI 100 mL ivgtt Qd combined with ①, ②, ③, ④, ⑤	①, ②, ③, ④, ⑤	15	NR	(4)	NR	NR
Shenfu injection												
Zhu (2006)	52/46	73/25	52.5/53.8	ALL AMI	NR	SFI ivgtt Qd combined with conventional therapy	Conventional therapy	28	NR	(4)	NR	NR
Zhang et al. (2018c)	60/60	76/44	62.97 ± 3.59/63.07 ± 3.6	STEMI	PCI	SFI 80 mL ivgtt st combined with ①, ⑩	The same dose of 0.9% saline control combined with ①, ⑩	1	30 d	(1)	Ⅵ	NR
Wang et al. (2021a)	20/20	35/5	50.4 ± 10.2/58.4 ± 8.6	STEMI	PCI	SFI 80 mL iv before surgery, and maintained for Qd combined with ⑩	Matched placebo, ⑩	5	28 d	(3)	ⅡⅥ	NR
Shen et al. (2006)	83/82	88/77	59/61	STEMI	Thrombolytic therapy	SFI 80–100 mL ivgtt Qd combined with conventional therapy, ②, ⑥, ⑨	Conventional therapy combined with ②, ⑥, ⑨	7	NR	(1) (4)	NR	NR
Zhang et al. (2019)	33/32	41/24	NR	STEMI	PCI	SFI 60 mL ivgtt Qd combined with conventional therapy, ⑩	Conventional therapy, ⑩	10	2 M	(4)	NR	NR
Zhang et al. (2011)	36/38	38/36	54.2/55.7	AMI	NR	SFI 60 mL ivgtt Qd combined with conventional therapy	Conventional therapy	14	NR	(1)	NR	NR
Zhu et al. (2020)	70/70	82/58	65.07 ± 7.24/61.67 ± 6.42	NSTEMI	PCI	SFI 60 mL ivgtt Qd combined with ①, ③, ④, ⑤, ⑩	①, ③, ④, ⑤, ⑩	10	30 d	(1)	Ⅵ	NR
Ma et al. (2022)	55/55	61/49	57.61 ± 2.1/57.62 ± 2.11	NSTEMI	NR	SFI 60 mL ivgtt Qd combined with ①, ③, ④, ⑤	①, ③, ④, ⑤	10	NR	(4)	NR	NR
Pei et al. (2019)	36/36	36/34	63.04 ± 4.69/62.38 ± 5.14	STEMI	Thrombolytic therapy	SFI 60 mL ivgtt Qd combined with ①, ②, ③, ⑧	①, ②, ③, ⑨	14	NR	NR	Ⅷ	YES
Li et al. (2017)	67/67	75/59	51.2 ± 8.2/51.4 ± 8.3	STEMI	Thrombolytic therapy	SFI 60 mL ivgtt Qd combined with ①, ②, ③, ④, ⑥, ⑧	①, ②, ③, ④, ⑥, ⑨	14	NR	(4)	Ⅷ	NR
Ma et al. (2019)	55/55	63/47	59.14 ± 4.21/59.12 ± 4.22	ALL AMI	Thrombolytic therapy	SFI 60 mL ivgtt Bid combined with ①, ②, ③, ⑧, ⑨, ⑭	①, ②, ③, ⑧, ⑨, ⑭	14	NR	(4)	NR	NR
Li (2015a)	32/32	43/21	63.5 ± 11.2/63.2 ± 11.5	ALL AMI	Thrombolytic therapy	SFI 60 mL ivgtt Bid combined with ①, ②, ③, ④, ⑤, ⑨, ⑬	①, ②, ③, ④, ⑤, ⑨, ⑬	14	NR	(1) (4)	NR	NR
Zeng (2005)	54/56	61/49	57.6 ± 15.2/56.8 ± 15.7	ALL AMI	Thrombolytic therapy	SFI 60–100 mL ivgtt Qd combined with ②, ⑥, ⑧, ⑨	②, ⑥, ⑧, ⑨	10	NR	(1)	NR	NR
Mo and Zhao (2002)	36/38	40/34	55.3 ± 15.6/54.9 ± 12.7	ALL AMI	Thrombolytic therapy	SFI 60–100 mL ivgtt Qd combined with ②, ⑥, ⑧, ⑨	②, ⑥, ⑧, ⑨	7	NR	(1)	NR	NR
Chen et al. (2018)	29/29	27/31	54.84 ± 13.93/55.61 ± 14.32	STEMI	NR	SFI 50 mL iv-vp Qd combined with ①, ③, ④, ⑤, ⑥	①, ③, ④, ⑤, ⑥	21	NR	(4)	NR	NR
Liu (2018)	50/50	53/47	62.4 ± 8.8/63.3 ± 9.1	ALL AMI	NR	SFI 50 mL ivgtt Qd combined with conventional therapy, ③	Conventional therapy, ③	21	NR	(4)	Ⅵ	NR
Meng (2014)	30/30	43/17	46.3 ± 11.9/46.7 ± 12.1	STEMI	NR	SFI 50 mL ivgtt Qd combined with ⑧	⑧	5	NR	(4)	NR	NR
Feng et al. (2011)	37/31	37/31	61.2/60.5	ALL AMI	PCI	SFI 50 mL ivgtt Qd combined with ②, ⑤, ⑥, ⑩	②, ⑤, ⑥, ⑩	14	NR	(4)	NR	NR
Kang (2017)	31/31	26/36	54.21 ± 3.52/55.32 ± 3.29	STEMI	NR	SFI 50 mL ivgtt Qd combined with ①, ③, ⑤	①, ③, ⑤	14	NR	(4)	NR	NR
Yan et al. (2017)	40/40	43/37	61.68 ± 7.54/62.03 ± 7.66	STEMI	NR	SFI 50 mL ivgtt Qd combined with ①, ③, ④, ⑤, ⑥	①, ③, ④, ⑤, ⑥	21	NR	(4)	NR	NR
Wang et al. (2018a)	58/58	71/45	60.8 ± 2.5/64.8 ± 2.5	ALL AMI	NR	SFI 50 mL ivgtt once combined with ②, ③, ⑤, ⑧	②, ③, ⑤, ⑧	1	6 M	(4)	NR	NR
Yang et al. (2014)	40/40	56/24	70.4 ± 5.2/71.1 ± 4.2	STEMI	PCI OR CABG	SFI 50 mL ivgtt Bid combined with ①, ④, ⑤, ⑨	①, ④, ⑤, ⑨	10	NR	(4)	NR	NR
Lan et al. (2021)	20/20	23/17	58.81 ± 15.21/57.37 ± 17.13	STEMI	PCI	SFI 40 mL iv-vp Qd combined with ①, ③, ⑥, ⑩	①, ③, ⑥, ⑩	7	NR	(4)	NR	NR
Li (2006)	37/36	52/21	63.3 ± 16.9/59.8 ± 17.2	STEMI	NR	SFI 40 mL ivgtt Qd combined with conventional therapy, ⑥, ⑬	Conventional therapy combined with ⑥, ⑬	14	NR	(1) (4)	NR	NR
Wang et al. (2018b)	31/31	32/30	NR	ALL AMI	NR	SFI 40 mL ivgtt Qd combined with ⑧	⑧	7	6 M	(1) (4)	NR	NR
Feng et al. (2019)	174/160	197/137	60.79 ± 9.73/61.43 ± 7.22	STEMI	NR	SFI 40 mL ivgtt Qd combined with ①, ②, ④, ⑤, ⑬	①, ②, ④, ⑤, ⑬	10	NR	(1) (4)	NR	NR
Zhao et al. (2016a)	31/30	32/30	NR	AMI including STEMI and non-STEMI in one study	PCI	SFI 40 mL ivgtt Qd combined with ①, ②, ③, ④, ⑥, ⑧, ⑩	①, ②, ③, ④, ⑤, ⑧, ⑩	7	6 M	(1) (4)	NR	NR
Hao et al. (2021)	49/48	42/55	56.98 ± 4.02/57.47 ± 3.98	ALL AMI	PCI	SFI 40 mL iv, SFI 40 mL ivgtt Qd combined with ①, ⑩	①, ⑩	7	1 M	(1)	NR	NR
Li (2015b)	42/41	51/32	56/61	STEMI	Thrombolytic therapy	SFI 40 mL iv every 15 min for 4–8 consecutive times combined with ②, ⑥, ⑨	②, ⑥, ⑨	2	NR	(1) (4)	NR	NR
Chen et al. (2003)	40/38	50/28	NR	STEMI	Thrombolytic therapy	SFI 30 mL ivgtt Qd combined with ①, ②, ⑥, ⑧	①, ②, ⑥, ⑧	14	NR	(4)	NR	NR
Li (2006)	37/36	49/24	63.7 ± 18.6/59.8 ± 17.2	STEMI	NR	SFI 20 mL ivgtt Qd combined with conventional therapy, ⑥, ⑬	Conventional therapy combined with ⑥, ⑬	14	NR	(1) (4)	NR	NR
Li et al. (2010)	58/34	55/37	68.2 ± 9.33/67.8 ± 10.72	STEMI	NR	SFI 1 mL/kg ivgtt Qd combined with ①, ④, ⑤, ⑥, ⑧, ⑬	①, ④, ⑤, ⑥, ⑧, ⑬	14	NR	(4)	NR	NR
Wang et al. (2017)	64/64	74/54	59.7 ± 14.3/58.2 ± 13.6	AMI including STEMI and non-STEMI in one study	PCI	SFI 10 mL/h iv-vp combined with ①, ②, ③, ⑤, ⑥, ⑧, ⑬	①, ②, ③, ⑤, ⑥, ⑧, ⑬	7	NR	(1)	NR	NR
Li et al. (2016)	32/32	40/24	NR	ALL AMI	PCI	SFI 100 mL ivgtt Qd combined with conventional therapy, ①, ②, ⑧, ⑩	Conventional therapy combined with ①, ②, ⑧, ⑩	No more than 14	NR	(1)	NR	NR
Wen (2014)	31/31	36/26	NR	STEMI	Thrombolytic therapy	SFI 100 mL ivgtt Qd combined with ②, ⑨	②, ⑨	15	NR	(4)	NR	NR
Li and Cheng (2014)	35/36	50/21	62.7 ± 16.6/61.8 ± 15.2	STEMI	PCI	SFI 100 mL ivgtt Qd combined with ①, ②, ⑩	①, ②, ⑩	10–14	NR	(1)	NR	NR
Li (2013)	30/30	38/22	55.3 ± 15.6/54.9 ± 12.7	ALL AMI	Thrombolytic therapy	SFI 100 mL ivgtt Qd combined with ①, ②, ④, ⑤, ⑥, ⑨, ⑭	①, ②, ④, ⑤, ⑥, ⑨, ⑭	14	2 M	(4)	NR	NR
Zhang et al. (2023)	90/90	96/84	75.13 ± 7.26/72.56 ± 6.68	STEMI	NR	SFI 100 mL ivgtt Qd combined with ①, ②, ③, ⑧	①, ②, ③, ⑧	3	30 d	(4)	NR	NR
Li et al. (2017)	31/31	34/28	66.38 ± 10.69/67.41 ± 11.98	ALL AMI	NR	SFI 100 mL ivgtt Qd combined with ①, ②, ③, ④, ⑤, ⑥, ⑧, ⑬	①, ②, ③, ④, ⑤, ⑥, ⑧, ⑬	14	NR	(1) (4)	Ⅰ	NR
Zhu et al. (2019)	80/80	94/66	56.4 ± 2.2/56.9 ± 2.1	ALL AMI	NR	SFI 100 mL ivgtt Qd combined with ①, ②	Conventional therapy combined with ①, ②	14	NR	(1)	NR	NR
Astragalus injection												
Han et al. (2000)	38/44	50/32	54.3 ± 12.4/52.8 ± 11.7	STEMI	Thrombolytic therapy	AI 60 mL ivgtt Qd combined with ①, ②, ⑤, ⑥, ⑨	①, ②, ⑨	10	1 M	(1)	Ⅰ	NR
Mi et al. (2009)	30/29	36/23	62.33 ± 10.27/60.43 ± 10.27	ALL AMI	Thrombolytic therapy	AI 50 mL ivgtt Qd combined with conventional therapy,⑧, ⑨	Conventional therapy combined with ⑧, ⑨	14	NR	(1) (4)	NR	NR
Xian (2019)	48/48	53/43	65.27 ± 7.16/64.58 ± 7.32	ALL AMI	PCI	AI 20 mL ivgtt Qd combined with ①, ③, ⑤, ⑧, ⑩	①, ③, ⑤, ⑧, ⑩	14	NR	(4)	Ⅵ	NR

Note: N, number; E, experimental group; C, control group; M, male; F, female; Y, years old; AMI, acute myocardial infarction; STEMI, ST, segment elevation myocardial infarction; NSTEMI, non-ST, segment elevation myocardial infarction; ALL AMI, STEMI, and NSTEMI; PCI, percutaneous coronary intervention; CCB, calcium channel blockers; I, intervention measures; d, day; M, month; NR, not report; SGMI: shengmai injection; SMI, shenmai injection; SFI, shenfu injection; AI, astragalus injection; Tid, 3 times a day; Bid, twice a day; Qd, once a day; st, at once; ① anti-platelet; ② anticoagulation; ③ lipid lowering; ④ β -blocker; ⑤ ACEI/ARB; ⑥ antimyocardial ischemia; ⑦ amiodarone; ⑧ vasoactive drugs; ⑨ Thrombolytic therapy; ⑩ PCI; ⑪ Alleviation pain; ⑫ lidocaine; ⑬ Diuretic medication; ⑭ Sedatives; ⑮ CCB; Outcome: (1) fatality rate in hospitalization; (2) fatality rate in the long term; (3) incidence of malignant arrhythmia; (4) left ventricular ejection fraction; Adverse event: I: bleeding events; II: abnormal renal function; III: allergy; IV: headache; V: dizziness; VI: abnormal digestive system; VII: respiratory system dysfunction; VIII: ecchymosis; IX: rash.

### 3.3 Quality evaluation

**FIGURE 2:**
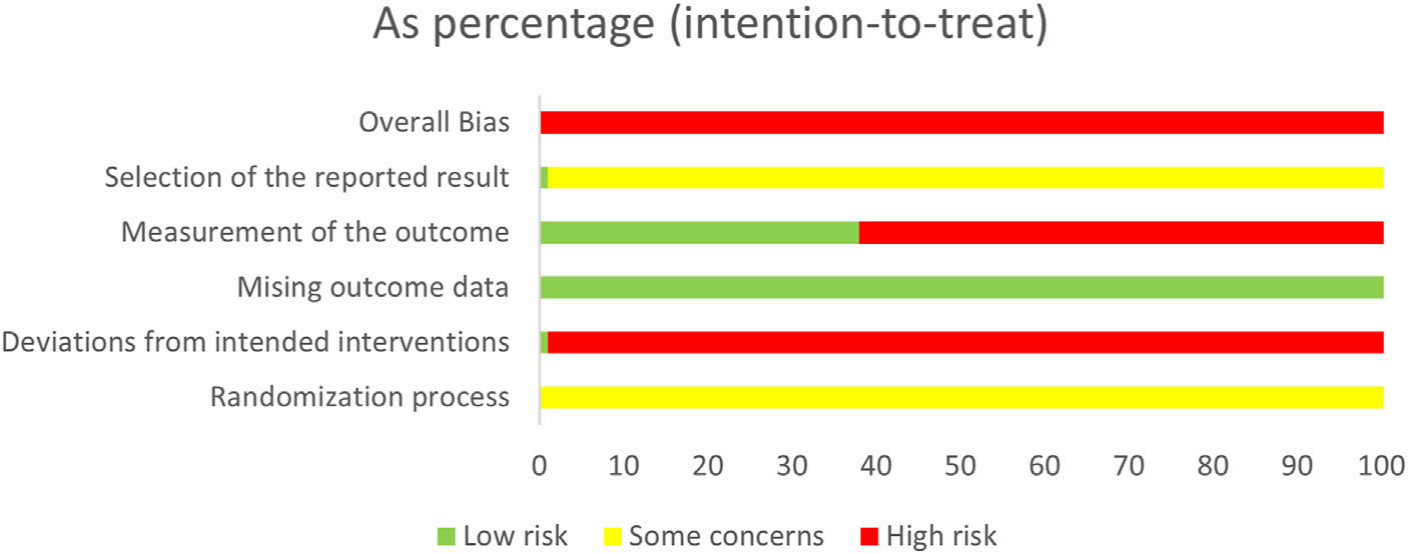
Risk-of-bias graph.

All studies (n = 113) were considered to be at high risk of bias. The results are presented in Figure 2 and Supplementary Figure S1. Regarding randomization process, 51 studies described specific randomization methods: 45 studies used the random number table method, one used the simple randomization method, one used the dice throwing method, one used the lottery method, one used the stratified randomization principle, one used the parity randomization method, and one used the randomized parallel grouping method. All included studies were deemed to some concerns risk due to inadequate description of allocation sequence concealment. Regarding deviations from the established intervention, one study specifically described the double-blind method and was rated as having a low risk of bias, and the rest of the studies did not describe the specific randomization or blinding methods and were rated as having a high risk of bias. All included studies were deemed to have a low risk of bias due to missing outcome data. 38 studies assessed no effect on outcome measures and were judged to be at low risk of bias. One study was registered on clinicaltrials.gov and presented all results, so it was judged to be at low risk of bias, while the rest of the studies did not mention registration and were assessed as being at some concerns risk.

### 3.4 Results of the meta-analysis

#### 3.4.1 Case fatality rate

**FIGURE 3:**
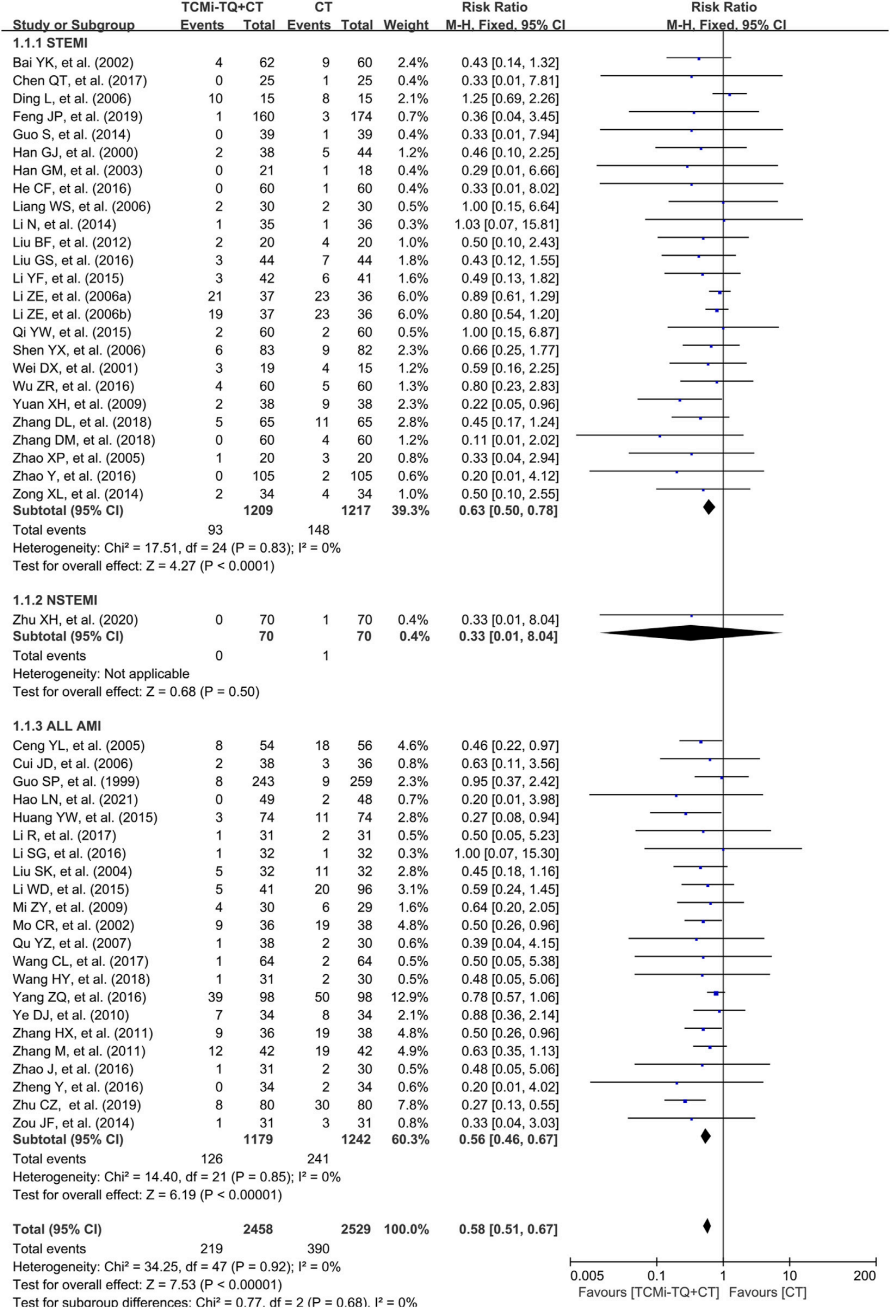
Forest plot of the effect of TCMi-TQ combined with conventional biomedicine treatment on in-hospital mortality in patients with AMI Notes: CI, confidence interval; TCMi-TQ, Traditional Chinese medicine injections for Tonifying Qi; CT, conventional treatment; AMI, acute myocardial infarction; STEMI, ST-segment elevation myocardial infarction; NSTEMI, non-ST-segment elevation myocardial infarction.

**FIGURE 4:**
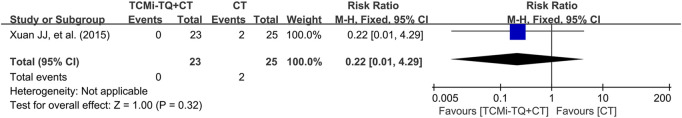
Forest plot of the effect of TCMi-TQ combined with conventional biomedicine treatment on long-term mortality in patients with AMI Notes: CI, confidence interval; TCMi-TQ, Traditional Chinese medicine injections for Tonifying Qi; CT, conventional treatment.

49 studies (Hao et al., 2021; Zhu et al., 2020; Feng et al., 2019; Zhu et al., 2019; Wang HY. et al., 2018; Zhang DL. et al., 2018; Zhang DM. et al., 2018; Li and Hou, 2017; Wang and Qing, 2017; Chen, 2017; He et al., 2016; Li, 2016; Liu, 2016; Wu et al., 2016; Qi et al., 2015a; Xuan et al., 2015; Li and Cheng, 2014; Zong et al., 2014; Zou, 2014; Liu, 2012; Zhang, 2011; Zhang et al., 2011; Ye, 2010; Mi et al., 2009; Yuan, 2009; Qu, 2007; Cui and Li, 2006; Ding and Xu, 2006; Li, 2006; Liang, 2006; Shen et al., 2006; Zeng, 2005; Zhao et al., 2005; Liu et al., 2004; Han et al., 2003; Bai et al., 2002; Mo and Zhao, 2002; Wei and Liu, 2001; Han et al., 2000; Guo and Zhang, 1999) reported the case fatality rate involving 4,939 patients (Figure 3). The analysis showed no significant heterogeneity (I^2^ = 0%), and a fixed-effects model was used for statistical analysis. The meta-analysis results demonstrated that the combined application of TCMi-TQ significantly reduced the mortality of AMI patients compared to CT alone [RR = 0.58, 95%CI (0.51, 0.67), *P* < 0.05]. This effect was observed in both the STEMI subgroup [RR = 0.53, 95%CI (0.50, 0.78), *P* < 0.05] and the subgroup with ALL AMI cases [RR = 0.56, 95%CI (0.46, 0.67), *P* < 0.05]. However, in the long-term mortality subgroup (follow-up time >12 months), the combined application of TCMi-TQ did not significantly reduce the mortality of AMI patients compared to CT alone [RR = 0.22, 95%CI (0.01, 4.29), *P* = 0.32] (Figure 4).

#### 3.4.2 Malignant arrhythmia

**FIGURE 5:**
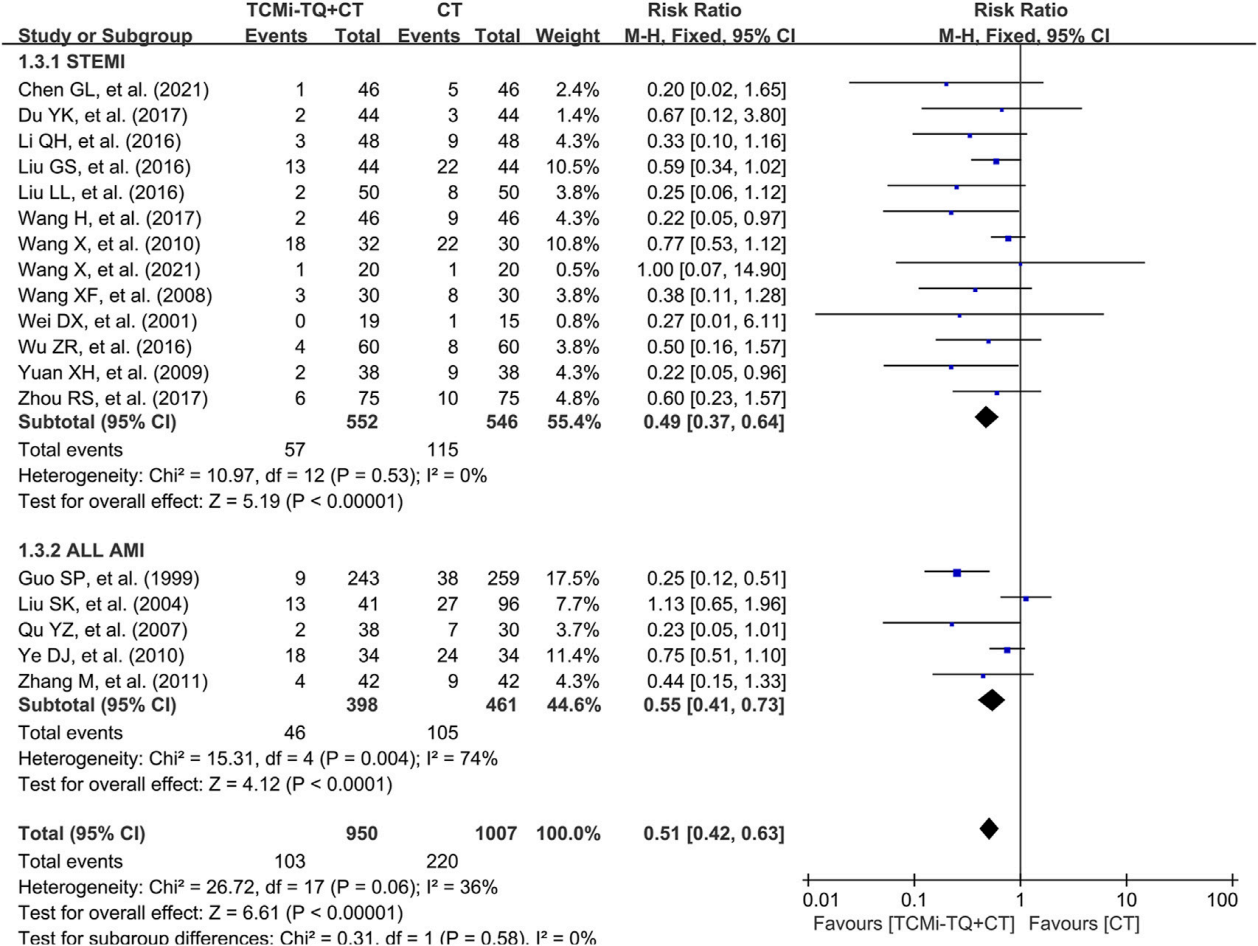
Forest plot of the effect of TCMi-TQ combined with conventional biomedicine treatment on the incidence of malignant arrhythmia in patients with AMI. Notes: CI, confidence interval; TCMi-TQ, Traditional Chinese medicine injections for Tonifying Qi; CT, conventional treatment; AMI, acute myocardial infarction; STEMI, ST-segment elevation myocardial infarction.

Malignant arrhythmia was reported in 18 studies (1957 patients) (Chen, 2021; Du, 2017; Wang et al., 2017; Zhou, 2017; Li et al., 2016; Liu, 2016; Liu and Tu, 2016; Wu et al., 2016; Zhang et al., 2011; Wang et al., 2010; Ye, 2010; Yuan, 2009; Wang, 2008; Qu, 2007; Liu et al., 2004; Wei and Liu, 2001; Guo and Zhang, 1999; Wang X. et al., 2021). These studies recorded ventricular fibrillation, polymorphic ventricular tachycardia, and second- or third-degree atrioventricular block with hemodynamic disturbances. The meta-analysis, with low heterogeneity between studies (I2 = 36%), indicated that the combination of TCMi-TQ and CT further reduced the incidence of malignant arrhythmia in AMI patients [RR = 0.51.95%CI (0.42, 0.63), *P* < 0.05]. This effect was observed in both the STEMI subgroup [RR = 0.49.95%CI (0.37, 0.64), *P* < 0.05] and the subgroup with ALL AMI cases [RR = 0.55.95%CI (0.41, 0.73), *P* < 0.05]. (Figure 5).

#### 3.4.3 LVEF

**FIGURE 6:**
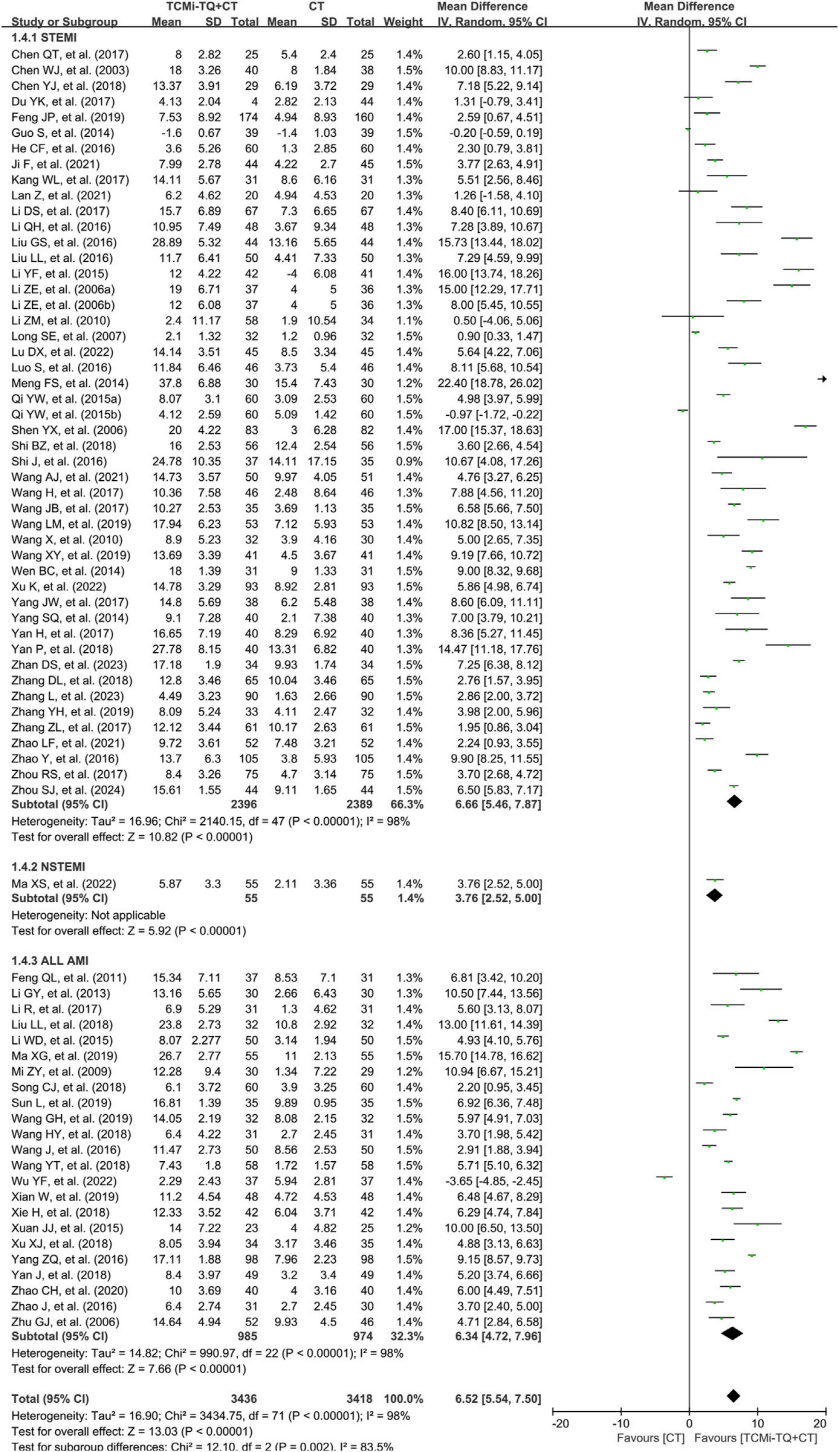
Forest plot of the effect of TCMi-TQ combined with conventional biomedicine treatment on LVEF in patients with AMI Notes: CI, confidence interval; MD, mean difference; TCMi-TQ, Traditional Chinese medicine injections for Tonifying Qi; CT, conventional treatment; AMI, acute myocardial infarction; STEMI, ST-segment elevation myocardial infarction; NSTEMI, non-ST-segment elevation myocardial infarction; LVEF, left ventricular ejection fraction.

LVEF data were reported in 71 studies (Zhou, 2024; Zhan and Cui, 2023; Zhang et al., 2023; Lu and Yao, 2022; Ma et al., 2022; Wu et al., 2022; Xu, 2022; Ji et al., 2021; Wang AJ. et al., 2021; Lan et al., 2021; Zhao and Sun, 2021; Zhao, 2020; Feng et al., 2019; Sun, 2019; Wang, 2019; Wang LM. et al., 2019; Wang XY. et al., 2019; Xian, 2019; Zhang et al., 2019; Chen and Qiao, 2018; Liu, 2018; Shi et al., 2018; Song, 2018; Wang HY. et al., 2018; Wang, 2018; Xie, 2018; Xu, 2018; Yan, 2018; Yan et al., 2018; Zhang DL. et al., 2018; Wang and Huang, 2017; Du, 2017; Kang, 2017; Li et al., 2017; Li and Hou, 2017; Wang et al., 2017; Yan et al., 2017; Yang JW. et al., 2017; Zhang, 2017; Zhou, 2017; Chen, 2017; He et al., 2016; Li et al., 2016; Liu, 2016; Liu and Tu, 2016; Luo, 2016; Wang, 2016; Yang and Cai, 2016; Zhao J. et al., 2016; Zhao Y. et al., 2016; Li WD., 2015; Li YF., 2015; Qi et al., 2015b; Qi et al., 2015c; Xuan et al., 2015; Guo, 2014; Meng, 2014; Wen, 2014; Yang, 2014; Li, 2013; Feng et al., 2011; Shi and Li, 2016; Li et al., 2010; Wang et al., 2010; Mi et al., 2009; Long, 2007; Li, 2006; Shen et al., 2006; Zhu, 2006; Chen et al., 2003). High heterogeneity was observed between these studies (I^2^ = 98%), and no clear sources of heterogeneity were identified through subgroup analysis (mean age, type of CCPP, treatment duration, sample size) (see Supplementary Figures S2–S5; Table 2 for details). Despite the heterogeneity, which was deemed acceptable in the overall population analysis, a random-effects model was employed. The meta-analysis results revealed that TCMi-TQ combined with CT significantly improved LVEF in both STEMI and NSTEMI patients compared to treatment with biomedicine alone [MD = 6.52, 95%CI (5.54, 7.50), *P* < 0.05] (Figure 6).

**TABLE 2:** Subgroup analysis of LVEF based on mean age, TCMi-TQ category, duration of treatment, and sample size.

Grouping criteria	Subgroups	N	*I* ^2^ (%)	MD (95%*CI*)	*Z*	*P*
Average age	≥60 years old	34	96	5.23 (4.07, 6.39)	8.82	<0.00001
	60 years old > age ≥40 years old	31	99	7.55 (5.87, 9.22)	8.81	<0.00001
	No report	7	96	7.93 (5.26, 10.61)	5.81	<0.00001
TCMi-TQ variety	Shengmai injection	6	91	4.87 (4.32, 5.43)	17.29	<0.00001
	Shenmai injection	36	98	3.79 (3.62, 3.95)	45.03	<0.00001
	Shenfu injection	28	97	7.51 (7.25, 7.77)	56.36	<0.00001
	Astragalus injection	2	72	7.16 (5.49, 8.83)	8.41	<0.00001
Sessions	≤7 days	22	98	4.00 (3.79, 4.21)	36.84	<0.00001
	>7 days	49	98	5.50 (5.32, 5.67)	60.99	<0.00001
	No report	1	-	3.70 (2.68, 4.72)	7.08	<0.00001
Sample size	<100 people	46	98	4.55 (4.37, 4.72)	50.57	<0.00001
	≤100 people	26	98	5.32 (5.11, 5.53)	49.83	<0.00001

Notes: N, number of studies; CI, confidence interval; MD, mean difference; TCMi-TQ, traditional Chinese medicine injections for Tonifying Qi.

#### 3.4.4 Adverse events

**FIGURE 7:**
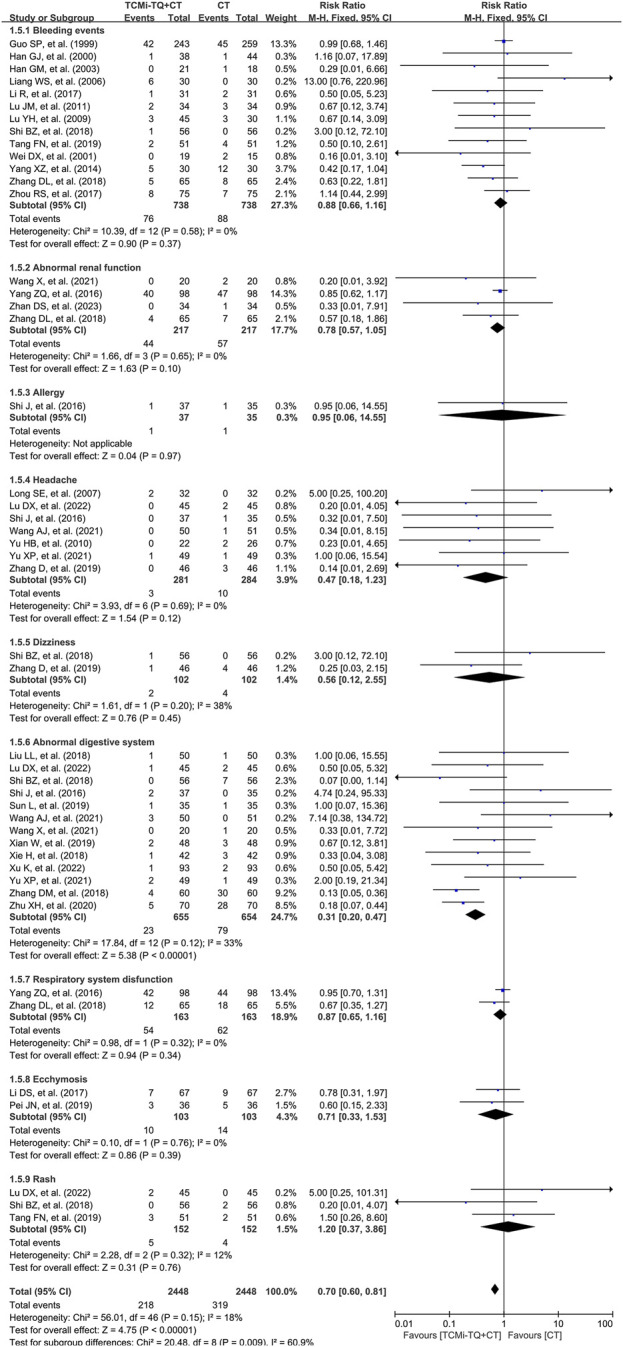
Forest plot of the occurrence of adverse events in patients with AMI treated with TCMi-TQ combined with conventional biomedicine. Notes: CI, confidence interval; RR, risk ratio; TCMi-TQ, Traditional Chinese medicine injections for Tonifying Qi; CT, conventional treatment.

Adverse events were reported in 32 studies (4,896 patients) (Zhan and Cui, 2023; Lu and Yao, 2022; Xu, 2022; Wang AJ. et al., 2021; Yu et al., 2021; Zhu et al., 2020; Pei et al., 2019; Sun, 2019; Tang, 2019; Xian, 2019; Zhang and Jia, 2019; Liu, 2018; Shi et al., 2018; Xie, 2018; Zhang DL. et al., 2018; Zhang DM. et al., 2018; Li et al., 2017; Li and Hou, 2017; Zhou, 2017; Yang and Cai, 2016; Yang et al., 2014; Lu, 2011; Shi and Li, 2016; Yu et al., 2010; Lu, 2009; Long, 2007; Liang, 2006; Han et al., 2003; Wei and Liu, 2001; Han et al., 2000; Guo and Zhang, 1999; Wang X. et al., 2021). These studies recorded bleeding events, abnormal renal function, allergies, headaches, dizziness, abnormal digestive system, respiratory system dysfunction, ecchymosis, and rash. The meta-analysis, with low heterogeneity between studies (I^2^ = 18%), indicated that the combination of TCMi-TQ and CT further reduced the incidence of adverse events in AMI patients [RR = 0.70.95%CI (0.60, 0.81), *P* < 0.05] (Figure 7). Specifically, the combination of TCMi-TQ and conventional treatment (CT) reduced the incidence of abnormal digestive system events in AMI patients [RR = 0.31, 95% CI (0.20, 0.47), *P* < 0.05], with heterogeneity I^2^ = 33%. Additionally, the combined use of TCMi-TQ and CT did not increase the risk of adverse events such as bleeding events, abnormal renal function, allergies, headaches, dizziness, respiratory system disfunction, ecchymosis, and rash (*P* > 0.05). These findings suggest that the combined use of TCMi-TQ and CT does not increase the incidence of adverse events (Table 3).

**TABLE 3:** Subgroup analysis of adverse events based on bleeding events, abnormal renal function, allergies, headaches, dizziness, abnormal digestive system, respiratory system dysfunction, ecchymosis, and rash.

Subgroups	Number of studies	Number of patients		I^2^ (%)	RR (95%CI)	Z	*P*
		TCMi-TQ + CT	CT				
Bleeding events	13	76 (738)	88 (738)	0	0.88 (0.66, 1.16)	0.90	0.37
Abnormal renal function	4	44 (217)	57 (217)	0	0.78 (0.57, 1.05)	1.63	0.65
Allergies	1	1 (37)	1 (35)		0.95 (0.06, 14.55)	0.04	0.97
Headaches	7	3 (281)	10 (284)	0	0.47 (0.18, 1.23)	1.54	0.12
Dizziness	2	2 (102)	4 (102)	38	0.56 (0.12, 2.55)	0.76	0.45
Abnormal digestive system	13	23 (655)	79 (654)	33	0.31 (0.20, 0.47)	5.38	<0.00001
Respiratory system dysfunction	2	54 (163)	62 (163)	0	0.87 (0.65, 1.16)	0.94	0.34
Ecchymosis	2	10 (103)	14 (103)	0	0.71 (0.33, 1.53)	0.86	0.39
Rash	3	5 (152)	4 (152)	12	1.20 (0.37, 3.86)	0.31	0.76

Notes: CI, confidence interval; CT, conventional treatment; RR, relative risk; TCMi-TQ, traditional Chinese medicine injections for Tonifying Qi.

#### 3.4.5 Bias and sensitivity analysis

**FIGURE 8:**
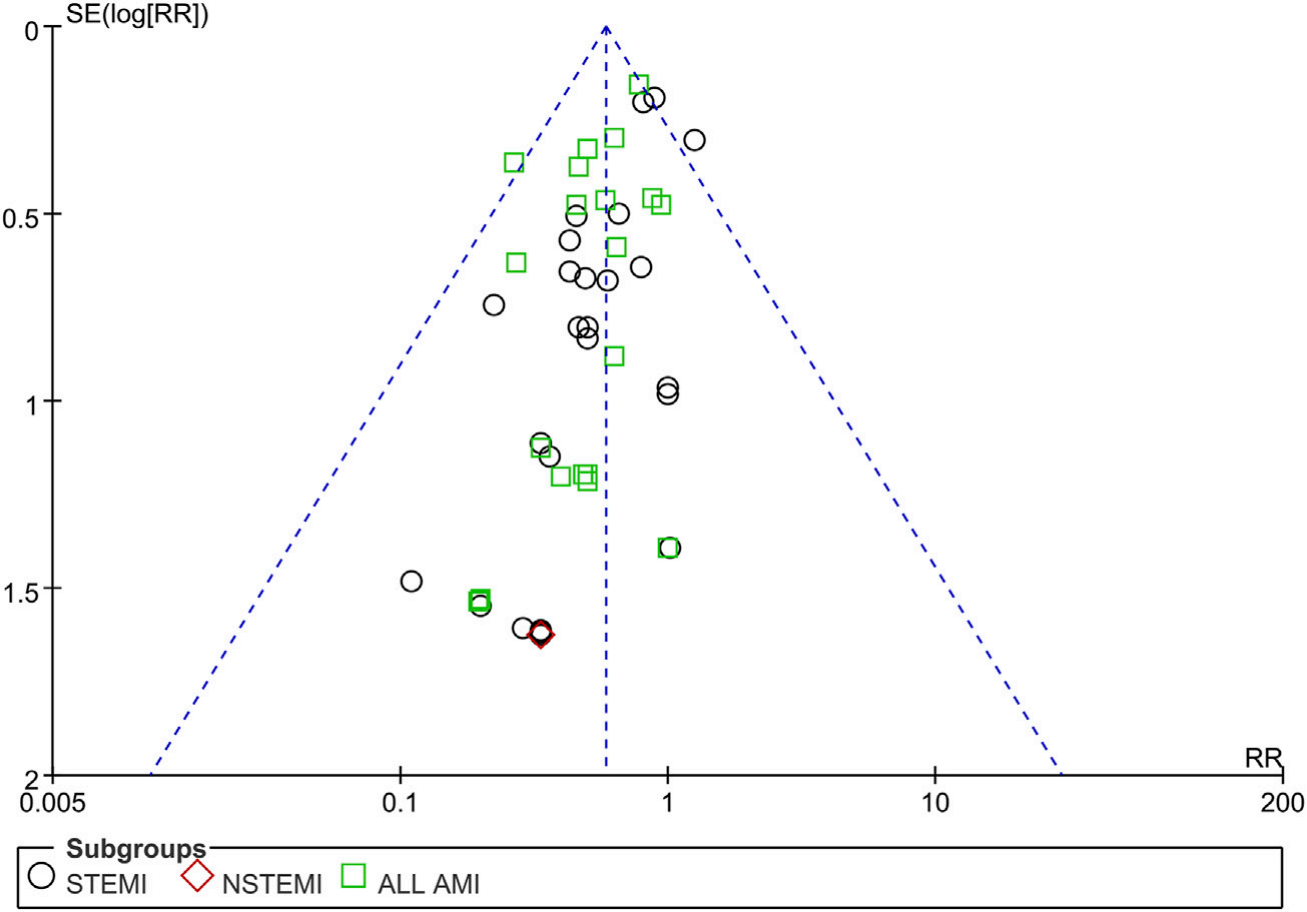
Funnel plot of case fatality rate. Notes: STEMI, ST-segment elevation myocardial infarction; NSTEMI, non-ST-segment elevation myocardial infarction; AMI, acute myocardial infarction; RR, risk ratio.

**FIGURE 9:**
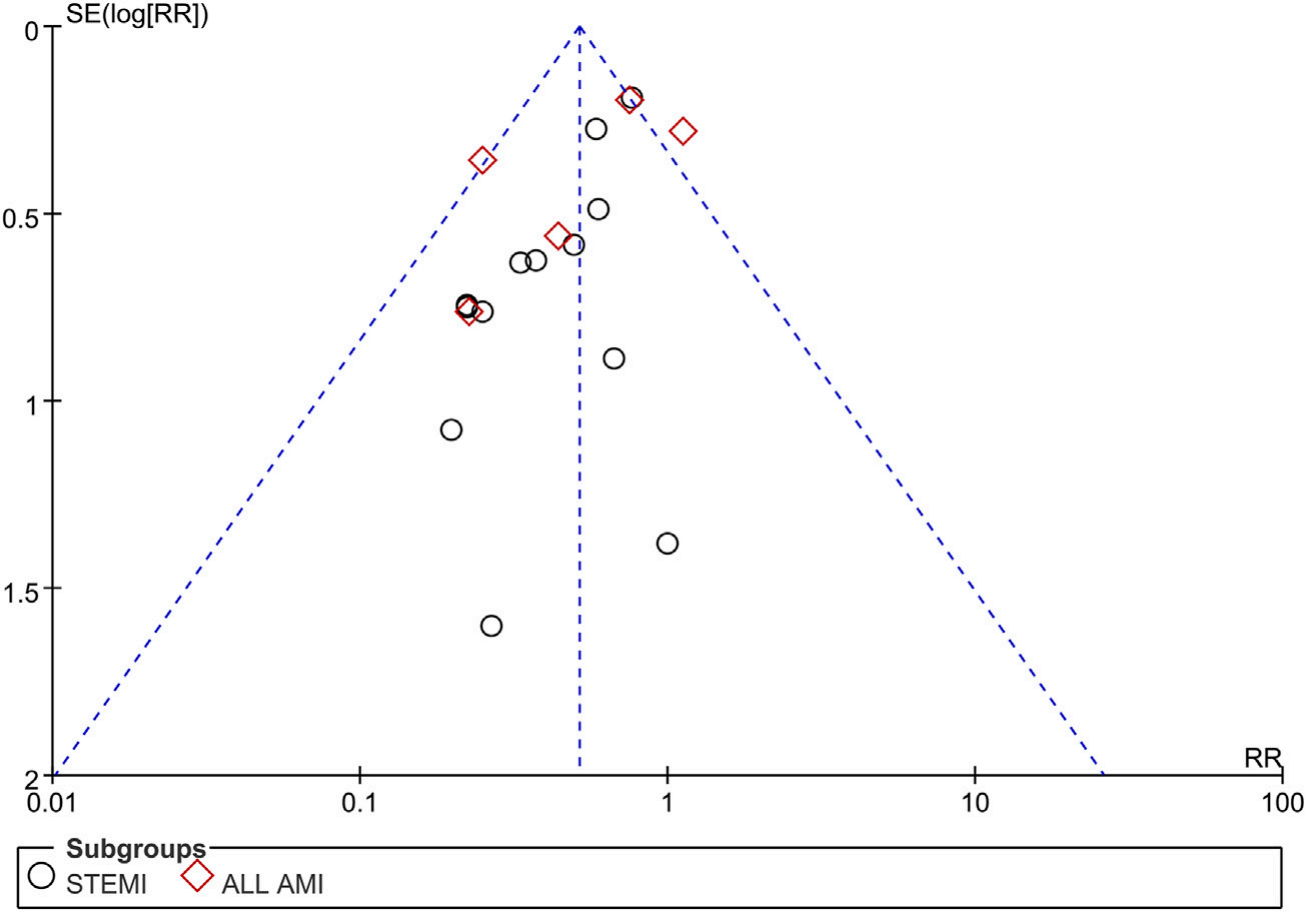
Funnel plot of malignant arrhythmia. Notes: STEMI, ST-segment elevation myocardial infarction; AMI, acute myocardial infarction; RR, risk ratio.

**FIGURE 10:**
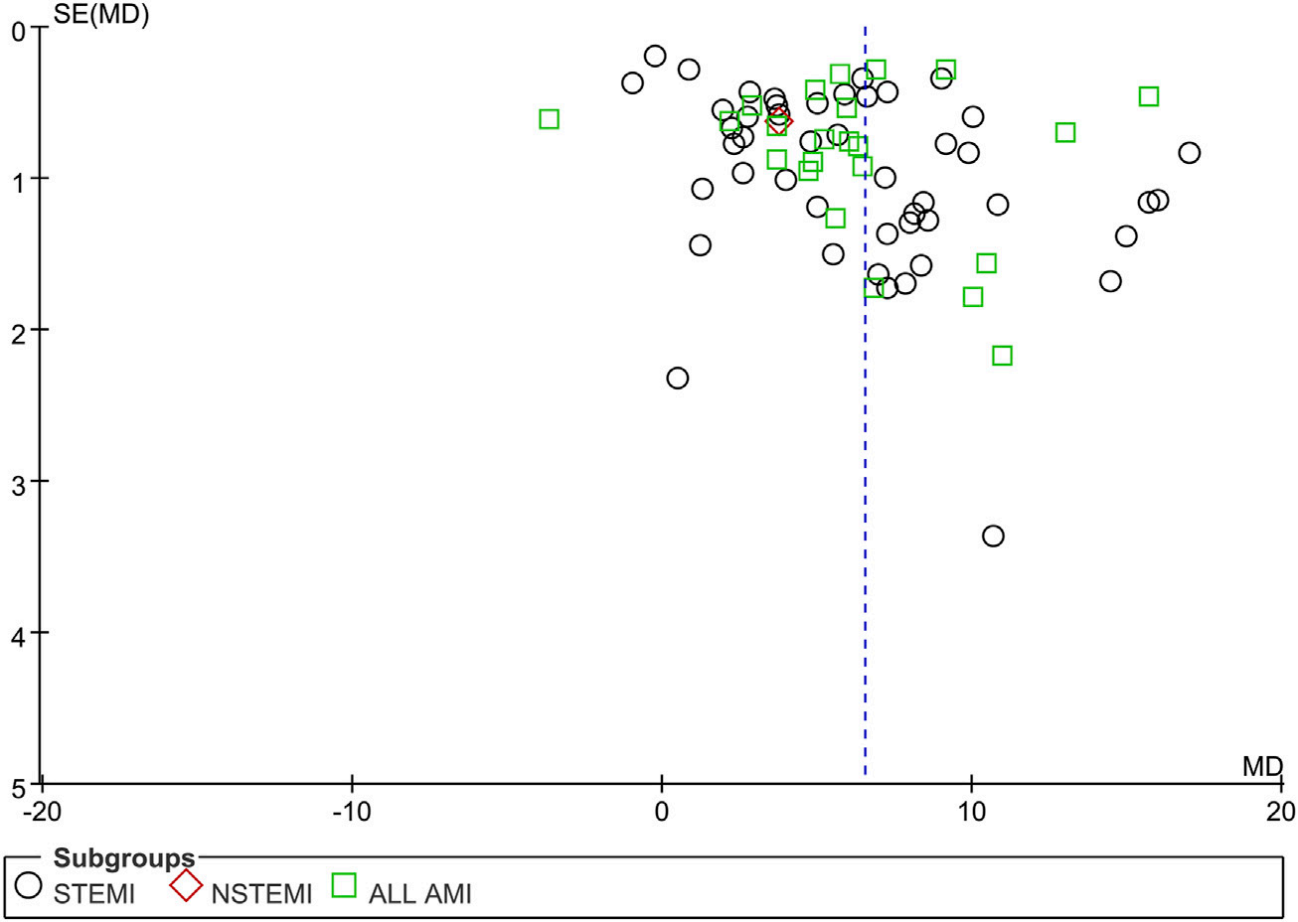
Funnel plot of LVEF Notes: STEMI, ST-segment elevation myocardial infarction; NSTEMI, non-ST-segment elevation myocardial infarction; AMI, acute myocardial infarction; LVEF, left ventricular ejection fraction; MD, mean difference.

**FIGURE 11:**
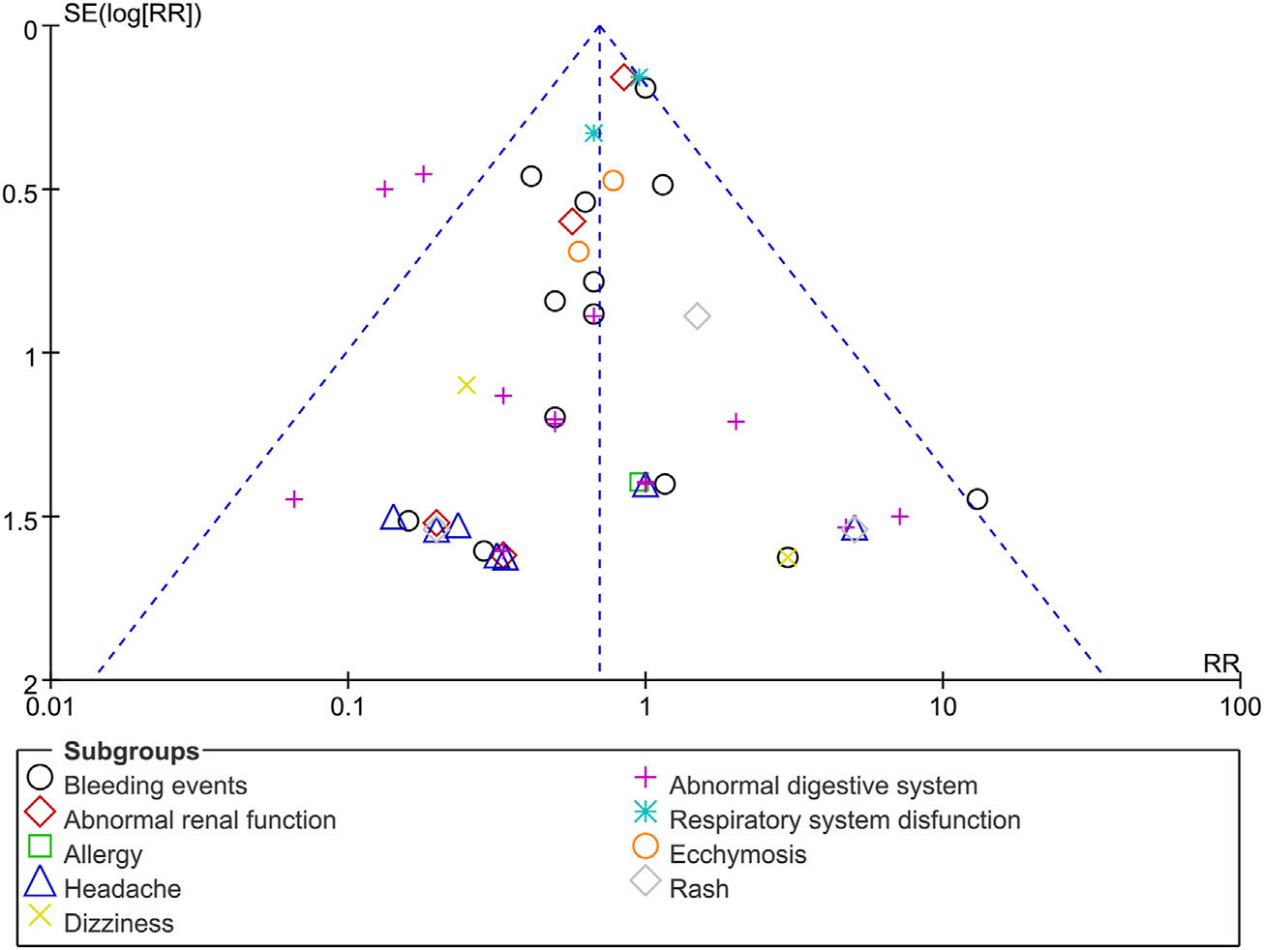
Funnel plot of adverse events. Notes: RR, risk ratio.

Funnel plots of mortality, malignant arrhythmia, LVEF, and adverse events are presented in Figures 8–11. The Egger test for two outcome indicators indicated no significant publication bias in LVEF (*P* = 0.199) and adverse events (*P* = 0.158). Mortality (*P* = 0.000) and malignant arrhythmia (*P* = 0.005) had significant publication bias. The results were corrected using the trim-and-fill method. No additional studies were included after two iterations using the Linear method. The fixed model results showed no change before and after the iterations, indicating that the meta-analysis results were stable.

Regarding other sources of bias, all included RCTs described the comparability of baseline data, indicating a low risk of bias. For LVEF, the results changed significantly after the removal of Feng et al. (2019) in STEMI, suggesting that this RCT may be the source of heterogeneity (Supplementary Figure S6). The results changed significantly after the removal of Wu et al. (2022) and Yang and Cai (2016) in all AMI cases, suggesting that these RCTs may be the sources of heterogeneity (Supplementary Figure S7). After excluding these three studies one by one, the estimated comprehensive effect points of the remaining studies did not exceed the range, and the results were relatively robust (Supplementary Figures S8–S10).

#### 3.4.6 Results of quality-of-evidence grading

The quality of evidence for the outcomes was assessed using the GRADE method. Due to limitations such as lack of blinding, insufficient allocation concealment, small sample sizes (less than 400 patients), and significant heterogeneity between studies, the quality of evidence for in-hospital mortality, malignant arrhythmias, and adverse events was rated as moderate. The quality of evidence for LVEF was rated as low. A detailed summary of the evidence for each outcome is provided in Table 4.

**TABLE 4:** Evidence summary of outcomes.

Certainty assessment							No. of patients		Effect		Certainty	Importance
No. of studies	Study design	Risk of bias	Inconsistency	Indirectness	Imprecision	Other considerations	TCMi-TQ+CT	CT	Relative (95% CI)	Absolute (95% CI)		
Case fatality rate												
48	randomised trials	serious^a^	not serious	not serious	not serious	none	219/2,458 (8.9%)	390/2,529 (15.4%)	RR 0.58 (0.51–0.67)	65 fewer per 1,000 (from 76 fewer to 51 fewer)	⊕⊕⊕○Moderate	CRITICAL
Fatality rate in the long term												
1	randomised trials	serious^a^	not serious	not serious	serious^b^	none	0/23 (0.0%)	2/25 (8.0%)	RR 0.22 (0.01–4.29)	62 fewer per 1,000 (from 79 fewer to 263 more)	⊕⊕○○Low	CRITICAL
Incidence of malignant arrhythmia												
18	randomised trials	serious^a^	not serious	not serious	not serious	none	103/950 (10.8%)	220/1,007 (21.8%)	RR 0.51 (0.42–0.63)	107 fewer per 1,000 (from 127 fewer to 81 fewer)	⊕⊕⊕○Moderate	IMPORTANT
LVEF												
72	randomised trials	serious^a^	serious^c^	not serious	not serious	none	3,436	3,418	-	MD 6.52 higher (5.54 higher to 7.5 higher)	⊕⊕○○Low	IMPORTANT
Adverse events												
32	randomised trials	serious^a^	not serious	not serious	not serious	none	218/2,448 (8.9%)	319/2,448 (13.0%)	RR 0.70 (0.60–0.81)	36 fewer per 1,000 (from 52 fewer to 25 fewer)	⊕⊕⊕○Moderate	IMPORTANT

Notes: CI, confidence interval; RR, risk ratio; MD, mean difference; TCMi-TQ, traditional Chinese medicine injections for Tonifying Qi; CT, conventional treatment; LVEF, left ventricular ejection fraction.

^a^ The blinding method and allocation concealment were not used.

^b^ Number of patients included was less than 400.

^c^ I square value was large.

## 4 Discussion

In China, the integration of TCM and biomedicine is increasingly becoming an anticipated model of medical development, as it contributes to addressing clinical issues more effectively. Taking AMI as an example, despite the rapid development of modern medical techniques, including PCI, in China, a turning point in the reduction of AMI mortality has not yet been observed (Tsao et al., 2022; GBD 2013 Mortality and Causes of Death Collaborators, 2015). Early intervention and diagnosis of diseases can reduce the incidence rate of AMI, but these areas need further research (Wang et al., 2024b; Wang et al., 2024c; Jaiswal et al., 2023). The standardized application of TCM may serve as a valuable approach to addressing this clinical issue. However, the process of integrating traditional and modern medicine requires support from high-quality evidence-based research. Our study contributes precisely to this by conducting relevant work.

Traditional Chinese Medicine injection (TCMi) refers to a sterile preparation extracted and purified from TCM, which can be in the form of a solution, emulsion, lyophilized powder, or concentrated solution (Zhang et al., 2021; Chen et al., 2022). It is known for its high bioavailability and precise therapeutic effects and has been widely used in China particularly in the treatment of AMI.

It is important to mention a concept in TCM known as “tong zheng yi bing” or “different diseases with the same pattern.” Specifically, even if it is not AMI, the same qi deficiency syndrome can be treated with medications that have the function of tonifying qi. Therefore, the use of TCMi may present issues with inappropriate indications. The TCMi selected in this study were those that have the function of tonifying qi. We identified four such TCMi through our search.

In Chinese medicine theory, qi is considered one of the fundamental substances that constitute the human body and maintain vital life activities. Functionally, qi serves roles in promoting, warming, defending, consolidating, and facilitating gasification. Thus, qi can regulate the blood, fluids, and essence; maintain body temperature; defend the body; and sustain the overall connectivity between the interior and exterior of the body. Qi transformation refers to the process of metabolism and the mutual transformation of energy among essence, blood, fluids, and other substances. None of this can be separated from the movement of qi; it can be said that qi, in its forms of ascending, descending, outgoing, and incoming, is the fundamental driving force of all life activities (Wang et al., 2023). It is evident that qi serves as the prime mover of all life activities within the human body. Mitochondria produce ATP, which is the primary source of energy for the body and the main source of power for cardiomyocytes, and the normal structure and function of mitochondria are crucial for myocardial energy metabolism (Lopaschuk and Jaswal, 2010). There is a correlation between qi and mitochondria in terms of their origin, morphology, function, and lesions (Lin et al., 2014; Zhang et al., 2001). Systematic reviews and meta-analyses of RCTs show that Qi-regulating formulations, such as Wenxin Keli and Yangxinshi tablet, may be effective and safe for treating ischemic heart disease (IHD) (Wang et al., 2016; Guo et al., 2023). Research h+as found that they play a certain role in regulating cardiac mitochondrial function (Wu et al., 2020), glucose metabolism, lipid metabolism, and amino acid metabolism (Zhang H. et al., 2018; Jiang et al., 2017). The active metabolite Ginsenoside Rb1 from Panax ginseng, known for its qi-tonifying effects, may promote myocardial recovery in AMI via mechanisms involving mitochondrial autophagy, as demonstrated by both *in vivo* and *in vitro* studies (Hu et al., 2022). Therefore, tonifying qi may have certain potential in regulating cardiac energy metabolism.

SGMI is made up of Ginseng Rubra Radix; Ophiopogonis Radix; Schisandrae Chinensis Fructus, and the main pharmacodynamic substances include ginsenoside metabolites and lignans. Clinical studies have demonstrated that SGMI can inhibit the inflammatory response in acute-phase AMI patients (Wang LM. et al., 2019). For patients in the recovery phase of AMI, SGMI can enhance clinical efficacy, boost cardiac function, improve tissue perfusion, and optimize oxygen metabolism (Luan et al., 2022). Additionally, it reduces levels of inflammatory factors (Lu and Yao, 2022), restores endothelial function (Tang, 2019), and improves hemorheological parameters (Wang, 2017). Ginsenosides, schizandrin, and ophiopogonin D are the primary active constituents of SGMI. Jiang et al. (2014) investigated the effects of this combination therapy on energy metabolism in rats with AMI and found that it can stimulate fatty acid oxidation and inhibit glycolysis, there by counteracting the metabolic reprogramming associated with AMI (Jiang et al., 2014). Li et al. (2019) found that SGMI can protect the mitochondrial structure of cardiomyocytes from Ang II-induced damage, stabilize mitochondrial membrane potential, and enhance mitochondrial oxygen utilization. Additionally, it can upregulate the expression of genes related to free fatty acid oxidation, glucose oxidation, and mitochondrial biogenesis by activating the adenosine monophosphate-activated protein kinase (AMPK) signaling pathway, which is crucial for energy metabolism (Li et al., 2019). Zhan et al. (2016) used comparative proteomics techniques to discover that SGMI may exert myocardial protection by modulating multiple energy metabolism pathways: promoting carbohydrate metabolism, inhibiting lipid metabolism, restoring the tricarboxylic acid cycle, and enhancing respiratory chain ATP production (Zhan et al., 2016).

SMI is a compound injection made of Ginseng Rubra Radix and ophiopogonis Radix, and the main pharmacodynamic substances include ginsenosides and ophiopogon saponins (Wang et al., 2020). Studies have found that SMI can alleviate oxidative stress in patients during the acute phase of AMI (Cao et al., 2022), improve vascular endothelial injury and apoptosis (Yang FF. et al., 2017), and enhance hemodynamic parameters (Qin, 2021). For patients in the recovery phase of AMI, SMI can effectively suppress inflammatory responses, reduce blood viscosity, and improve cardiac function (Zhou, 2024; Zhan and Cui, 2023). Wang et al. (2018) utilized network analysis to discover that SMI can significantly reverse the downregulation of energy metabolism-related proteins such as ATP synthase and malate dehydrogenase caused by ischemia, thereby modulating signaling pathways associated with oxidative phosphorylation and mitochondrial dysfunction. In a primary cardiomyocyte model of hypoxic injury in rats, they found that SMI can stabilize mitochondrial membrane potential, restore intracellular ATP levels, increase maximal mitochondrial respiration rate, and enhance oxygen reserve capacity, thus reversing energy metabolic imbalance (Wang Y. et al., 2018). Wang et al. (2019) found that SMI can reduce myocardial cell injury following ischemia-reperfusion (I/R). It increases the expression of glucose transporter 4, cluster of differentiation 36, and fructose-6-phosphate kinase, thereby enhancing the utilization of both free fatty acids and glucose (Wang S. et al., 2019).

SFI is made up of Ginseng Rubra Radix and Aconiti Lateralis Radix Praeparata (black shunpian), and the main active metabolites are ginsenosides and panaxynol (Zheng et al., 2022). For patients with acute-phase AMI, SFI can improve hemodynamic parameters (Zhuo et al., 2018) and reduce levels of inflammatory factors (Jin et al., 2017). For patients in the recovery phase of AMI, SFI can mitigate inflammatory responses (Li et al., 2017), improve hemorheological indicators (You and Wang, 2019), enhance fibrinolytic activity (Zhu et al., 2020), improve vascular endothelial function, and reduce oxidative damage (Jia et al., 2016). Studies have found that SFI can protect against myocardial injury by modulating mitochondrial dynamics, improving mitochondrial energy metabolism, reducing mitochondrial oxidative stress, and inhibiting structural damage to mitochondria (Lu and Xiang, 2023). Bai et al. (2018) investigated the effects of SFI on I/R injury in rats and found that it could enhance the clearance of oxygen free radicals, reduce cellular damage, reduce intracellular Ca^2+^ influx, increase ATP levels, and inhibit inflammation (Bo et al., 2018). Zhan et al. (2024) found that SFI can mediate mitochondrial autophagy in rats with I/R injury by regulating the HIF-1α/BNIP3 pathway, thereby protecting the mitochondrial structure and reducing myocardial cell apoptosis (Zhan et al., 2024). Ji et al. (2011) studied the effects of SFI on myocardial dysfunction following cardiac arrest and resuscitation in pigs and found that it could increase the activity of Na^+^-K^+^-ATPase and Ca^2+^-ATPase, and left ventricular superoxide dismutase, thereby modulating energy metabolism and enhancing antioxidant capacity (Ji et al., 2011). Additionally, Huang et al. (2020) found that Shenfu Formula could synergistically mediate metabolic flexibility of fatty acids and glucose in cardiac energy metabolism in heart failure mice induced by transverse aortic constriction through the AMPK-related pathway, thereby inhibiting cardiac metabolic remodeling (Huang et al., 2020).

AI is an injection made from Astragali Radix, and its main active metabolites include flavonoids, saponins, and amino acids (Yu H. et al., 2019). For patients with acute-phase AMI, AI can improve immune-inflammatory responses and ventricular remodeling (Hou et al., 2012). For patients in the recovery phase of AMI, AI can enhance cellular antioxidant capacity (Zhou et al., 2019), protect vascular endothelium, and increase overall antioxidant ability (Chen et al., 2015). Huang et al. (2018) investigated the effects of major extracts from Astragalus membranaceus on tert-butyl hydroperoxide-induced H9C2 cells and found that they could alleviate oxidative stress and increase cell survival by regulating mitochondrial membrane potential and enhancing mitochondrial bioenergetics parameters, including basal respiration, proton leak, maximal respiration, and non-mitochondrial respiration (Huang et al., 2018). Jin et al. (2014) found that Astragalus can correct impaired free fatty acid and glucose metabolism in AMI model rats, increase myocardial ATP, ADP, and total adenine nucleotide levels, thereby protect ischemic myocardium (Jin et al., 2014). Astragaloside IV, the primary active metabolite of AI, plays a crucial role in regulating cardiac energy metabolism. The underlying mechanisms likely involve multiple pathways: it induces the expression of mitochondria-related proteins (Wang Q. et al., 2021; Zang et al., 2020), protects the structural integrity of cardiac mitochondria (Lu et al., 2015), and modulates mitochondrial function (Dong et al., 2017).

These TCMi-TQs exhibit comparable effects. However, the safety of TCMi has become a growing concern. A retrospective investigation based on China PEACE revealed no benefits of TCMi in patients with acute heart failure (Yu Y. et al., 2019). The annual report on national adverse drug reaction monitoring (2023) revealed 2.627 million cases of suspected adverse drug reactions/events, of which traditional Chinese medicine accounted for 12.6%. Tonifying qi and yin drugs among the top five, and 25.9% of the cases involved injectable drug delivery (National Center For ADR Monitoring C, 2024). Considering the widespread use of TCMi-TQ in the AMI patient population (Spatz et al., 2018), it is necessary to conduct a high-quality systematic evaluation of its efficacy and safety.

This meta-analysis included 113 studies involving 10,779 participants. The results demonstrated that the combined application of TCMi-TQ was more effective in reducing in-hospital mortality, decreasing the occurrence of malignant arrhythmias, reducing the incidence of adverse events, and improving LVEF than biomedicine alone. Safety was also assessed in this meta-analysis, with 32 studies reporting on safety outcomes. No serious adverse events were observed, and the common adverse events included bleeding, ecchymosis, and gastrointestinal discomfort, which could be alleviated through drug withdrawal or symptomatic treatment.

Mortality rate is a crucial indicator reflecting the prognosis of AMI patients (Long et al., 2022). A retrospective study found no significant association between early application of TCMi and in-hospital bleeding or mortality rate in AMI patients (Spatz et al., 2018). However, our study revealed that the combined use of TCMi-TQ significantly reduced AMI mortality, which aligns with the findings of previous systematic reviews examining the effects of SGMI (Lu et al., 2018), SMI (Wang et al., 2015), SFI (Zhu et al., 2018), and AI (Su et al., 2017) in AMI treatment. These findings suggest that TCMi with the specific function of tonifying qi plays a unique role in reducing AMI mortality, possibly due to its comparable effects of regulating energy metabolism. Additionally, this study attempted to investigate the impact of TCMi-TQ combined with CT on the long-term mortality rate of AMI patients. However, due to the limited number of studies evaluating long-term mortality, we were unable to identify potential benefits of TCMi-TQ in long-term mortality, highlighting the need for further research.

Malignant arrhythmia is a significant cause of death in patients with acute myocardial infarction (AMI) (Eryol et al., 2002; Nasution et al., 2020). Studies have consistently demonstrated that malignant arrhythmia accompanies 60%–100% of deaths during the acute phase of AMI (Eldar et al., 1994; Berg et al., 2001). Unfortunately, the benefits of antiarrhythmic drugs for such patients are limited (Piccini et al., 2011). Although a few clinical cases have suggested that TCMi may have adverse effects leading to the occurrence of malignant arrhythmias (Jin, 2013; Zhao et al., 1995; Wu and Li, 1988), a meta-analysis revealed that the combined use of TCMi with tonifying qi properties can reduce the risk of malignant arrhythmias during hospitalization in AMI patients. This reduction in risk may be attributed to the clinical effect of TCMi-TQ in improving myocardial ischemia.

Following myocardial infarction, the loss of myocardial cells leads to myocardial remodeling and the development of heart failure (Author Anonymous, 2020), which significantly impacts the patients’ quality of life and long-term prognosis. LVEF is an essential indicator of cardiac function (McDonagh et al., 2021). This study found that the combined use of TCMi-TQ demonstrates clinically relevant improvements in cardiac function, consistent with previous meta-analysis results (Zhu et al., 2018; Wei et al., 2021). The protective effect of TCMi-TQ on ischemic myocardium may explain this improvement. However, significant heterogeneity was observed in the analysis results, and subgroup analysis and sensitivity analysis did not identify a clear source of heterogeneity. This heterogeneity may be attributed to differences in the ultrasound equipment and technical standards used for LVEF assessment. Therefore, caution must be exercised when interpreting the aforementioned results due to the presence of these heterogeneity factors.

Our meta-analysis results demonstrate that the combined administration of TCMi-TQ does not increase the occurrence of adverse events in AMI patients. Nevertheless, TCMi, when administered through direct bloodstream injection, can be influenced by various factors such as co-solvents, particulates during the manufacturing process, and solvents. This often leads to a higher occurrence of adverse reactions compared to other TCM formulations (Zhang and Niu, 2018). Therefore, healthcare providers should exercise caution in prescribing medications, prioritizing oral formulations. For patients with complex or severe conditions requiring traditional TCM injections, intramuscular administration should be preferred. In emergency situations, TCM injections via intravenous infusion may be necessary (Gao et al., 2012). Healthcare providers should strictly adhere to medication guidelines for the rational and standardized use of TCM injections. Providers should carefully prepare medications, standardize dosages and treatment plans, and accurately identify and document the evidence basis for medication use (Yu et al., 2023). Hospitals should enhance quality control and inspection during the procurement of medications. These TCM injections should be classified, stored separately, and subjected to enhanced supervision and consultation to ensure their proper use. Additionally, hospitals should integrate the quality of TCM intravenous formulations into clinical safety monitoring systems to enable traceability (Peng and Li, 2019). Assigning specialized TCM pharmacists to systematically manage these formulations can further enhance oversight and safety. Research has shown that pharmacist involvement in prescription review, dispensing, drug preparation, and patient counseling significantly reduces the incidence of adverse reactions (Deng et al., 2024). Therefore, hospitals should establish standardized management systems for TCMi and foster effective communication between pharmacists, clinical doctors, and patients to reduce the incidence of adverse reactions.

In summary, this study has the following characteristics compared to previous meta-analyses on TCMi (Mensah et al., 2023): The TCMi included in this study all possess the function of tonifying qi. This is because energy metabolism is one of the main therapeutic principles in TCM for treating AMI, reflecting the representativeness of this study (Suzuki et al., 2023). This study primarily focuses on the observing mortality rate. Meta-analyses of TCMi with mortality, a hard endpoint, as the primary outcome have been relatively rare. The conclusions of this study will provide a more valuable reference for clinical decision-making by healthcare professionals.

The present study underwent a rigorous research process, adhering strictly to a pre-registered protocol. Nonetheless, this meta-analysis still has certain limitations (Mensah et al., 2023): Regarding methodological quality, the overall quality of the included studies is suboptimal, particularly due to insufficient reporting of random sequence generation, allocation concealment methods, blinding implementation, medical follow-up, and independent assessment of the purity/potency of the TCMi-TQ utilized in the studies. These factors may introduce risks of selection bias and performance bias. Researchers should adhere to RCT design standards (Chan et al., 2013) and reporting guidelines (Butcher et al., 2022). In future clinical studies, independent collaborative laboratories should be incorporated, utilizing advanced technologies such as HPLC and GC-MS to assess the purity and efficacy of TCMi-TQ, accurately identify and quantitatively analyze active ingredients, and promote the scientific and standardized generation of high-quality clinical evidence in TCM. This will enhance the rigor and reliability of clinical trials (Suzuki et al., 2023). Regarding long-term efficacy, the included studies fail to evaluate long-term mortality, which limits the assessment of the long-term prognosis of AMI patients receiving TCMi-TQs. It is recommended to expand the evaluation of long-term survival outcomes in AMI patients who receive TCMi-TQs. This would provide a more comprehensive assessment of the clinical significance and practical application value of these treatments (National Center Cardiovascular Diseases, 2024). Regarding heterogeneity, there is notable heterogeneity in the results concerning LVEF, thereby affecting the certainty of the outcomes. These discrepancies may stem from variations in ultrasound equipment and technical standards for LVEF evaluation. To minimize the impact of human factors and ensure consistent, reliable results, it is recommended to enhance the standardization of ultrasound equipment and evaluation techniques (Roger et al., 2010). Regarding the generalizability of research results, all the included studies were conducted within China and involved a single ethnic group. While our findings provide a preliminary foundation for multicenter research, further evaluation is necessary to determine the generalizability of the conclusions. We recommend conducting multicenter trials outside of China to generate more reliable and generalizable clinical evidence.

## 5 Conclusion

The present study proposes that integrating TCMi-TQ with conventional biomedicine treatment has a favorable impact on reducing mortality rates, the incidence of malignant arrhythmias, the incidence of adverse events, and enhancing cardiac function among patients with AMI. Given the low methodological quality observed in the included studies, it is imperative to approach this conclusion with caution. Nevertheless, these findings hold significant potential for informing clinical practice guidelines, and we look forward to achieving the scientific integration of TCMi-TQ with standard care in the future.

## Data Availability

The original contributions presented in the study are included in the article/Supplementary Material, further inquiries can be directed to the corresponding authors.
