# Supplementary material for: Traditional Chinese medicine injections with Tonifying Qi, equivalent effect of regulating energy metabolism, for acute myocardial infarction: a systematic review and meta-analysis of randomized clinical trials
Source: Front Pharmacol. 2025 Mar 28;16:1511486. doi: 10.3389/fphar.2025.1511486 (PMC11985859; doi:10.3389/fphar.2025.1511486)
Supplement: Supplementary file 3 [file DataSheet1.DOCX]

**Supplementary table S1** Composition of the four TCMI-TQs***.***

| TCMI-TQs | Chinese drug name | Latin name | Botanical plant name | Family | Plant part used |
| --- | --- | --- | --- | --- | --- |
| Shengmai injection | Hongshen | Ginseng Rubra Radix et Rhizoma | Panax ginseng C.A.Mey. | Araliaceae | root |
|  | Maidong | Ophiopogonis Radix | Ophiopogon japonicus (Thunb.) Ker Gawl. | Asparagaceae | root tuber |
|  | Wuweizi | Schisandrae Chinensis Fructus | Schisandra chinensis (Turcz.) Baill. | Schisandraceae | fruit |
| Shenmai injection | Hongshen | Ginseng Rubra Radix et Rhizoma | Panax ginseng C.A.Mey. | Araliaceae | root |
|  | Maidong | Ophiopogonis Radix | Ophiopogon japonicus (Thunb.) Ker Gawl. | Asparagaceae | root tuber |
| Shenfu injection | Hongshen | Ginseng Rubra Radix et Rhizoma | Panax ginseng C.A.Mey. | Araliaceae | root |
|  | Huzi | Aconiti Lateralis Radix Praeparata | Aconitum carmichaelii Debeaux | Ranunculaceae | root |
| Astragalus injection | Huangqi | Astragali Radix | Astragalus mongholicus Bunge | Fabaceae | root |

**Supplementary table S2** Initial processing, indication, adverse reactions and contraindications of the four TCMI-TQs.

| TCMI-TQs | Description of the extract and extraction process | Indication, adverse reactions and contraindications | Source | Quality control  reported. (Y/N) |
| --- | --- | --- | --- | --- |
| Shengmai injection | Weigh 156 g of Schisandra chinensis, 312 g of Ophiopogon japonicus, and 100 g of red ginseng. The red ginseng should be ground into a fine powder and subsequently subjected to reflux extraction with ethanol five times, each extraction lasting two hours. The extracts are then combined, chilled, filtered, and the filtrate is concentrated into a thick paste. Add filtered water to the paste to obtain a final volume of 400 mL, stir thoroughly, chill, filter, and use the filtrate for further preparation. Collect 150 mL of Schisandra chinensis fruit juice via steam distillation, cool, and store it for subsequent use. The residue is then subjected to three water extractions, each lasting 40 minutes. The filtrates from each extraction are combined, filtered, and concentrated into a thick paste. Ethanol is added sequentially for two alcohol precipitations: initially to reach an ethanol concentration of 80%, followed by a second precipitation to achieve 85% ethanol concentration. The filtrate is filtered, ethanol is recovered, and the remaining filtrate is concentrated into a thick paste. The thick paste is mixed with 200 mL of sterile water, stirred, refrigerated, and then filtered. The filtrate is boiled with an appropriate quantity of activated carbon for 30 minutes, cooled slightly, and filtered until clear. The clear filtrate is used for subsequent preparation. The Ophiopogon japonicus aqueous solution was prepared using the same method as that for the Schisandra chinensis aqueous solution, with each solution having a final volume of approximately 200 mL. After equal mixing and filtration of the red ginseng extract, Schisandra distillate, Schisandra extract, and Ophiopogon extract, the combined filtrate is supplemented with water for injection to a total volume of 1000 mL. The solution's pH is adjusted to 7.5, followed by filtration, bottling, sterilization, and storage. | Indication: Nourishing yin and invigorating qi strengthens the pulse. This therapy is indicated for conditions such as myocardial infarction, cardiogenic shock, septic shock, palpitations, cold extremities, excessive sweating, and weak pulse.  Adverse reactions: According to the literature, this product has been associated with adverse reactions including erythematous maculopapules, pruritus, facial flushing, ocular edema, hypotension, anaphylactic shock, nausea and vomiting, abdominal distension, phlebitis, various forms of ventricular tachycardia, and sinus arrest.  Contraindications: 1. This product is contraindicated in individuals with a history of allergies or severe adverse reactions to its metabolites. 2. This product is contraindicated for use in infants and neonates. 3. This product is not recommended for individuals who exhibit signs of heat-related illnesses (such as summer heat) or those with unresolved exterior syndromes and associated symptoms like cough, despite having indications for its use. | 1.Jiangsu Suzhong Pharmaceutical Co., Ltd.  2.Pharmaceutical Factory, West China Medical University.  3.Sichuan Chuanda West China Pharmaceutical Co. Ltd.  4.Sanjiu Pharmaceutical Co., Ltd.  5.Made in Yibin, Sichuan. | Y-National Pharmaceutical  Standard: H20034120 |
| Shenmai injection | Red ginseng was extracted using the first extraction agent; the solvent was then removed, and water was added to obtain the red ginseng extract, adjusting its pH to 5.5–8.0. Ophiopogon japonicus was extracted using the second extraction agent; following solvent removal, water was added to obtain the Ophiopogon extract, which was adjusted to a pH of 5.0–8.0. The red ginseng extract and Ophiopogon extract were subjected to ultrafiltration, followed by combination. Both extraction agents consist of either organic solvents or mixtures of organic solvents and water, with the organic solvents being methanol, ethanol, or ethyl acetate. This process describes the preparation of Shenmai injection, an invention involving the aforementioned extraction methods. | Indication: Yiqi solid off, nourishing Yin and Jin, Sheng Mai. It is indicated for conditions such as shock, coronary heart disease, viral myocarditis, chronic cor pulmonale, and granulocytopenia associated with qi and Yin deficiency. It enhances the immune function in cancer patients, exhibits a synergistic effect when combined with chemotherapy agents, and mitigates chemotherapy-induced side effects.  Adverse reactions: 1. Allergic reactions: flushing, rash, pruritus, dyspnea, sensations of suffocation, palpitations, cyanosis, hypotension, laryngeal edema, anaphylactic shock, etc. 2. Systemic effects: chills, fever (including high fever), pain, fatigue, pallor, chest tightness, diaphoresis, syncope, etc. 3. Respiratory system: shortness of breath, cough, sneezing, asthma, etc. 4. Cardiovascular system: palpitations, chest tightness, chest pain, cyanosis, arrhythmias, tachycardia, hypertension, etc. 5. Digestive system: dry mouth, xerostomia, hiccups, nausea, vomiting, abdominal pain, diarrhea, constipation, flatulence, abnormal liver function tests, etc. 6. Nervous system: dizziness, cephalalgia, headaches, paresthesia, tremors, seizures, confusion, irritability, anxiety, insomnia, etc. 7. Skin and appendages: rash, maculopapular rash, erythema, urticaria, pruritus, edema, dermatitis, etc. 8. Medication site: pain, redness, numbness, itching, rash, phlebitis, etc.  9, others: back pain, myalgia, blurred vision and so on.  Contraindications: 1. This product, which contains red ginseng, Ophiopogon japonicus (maidong) preparations, and other listed metabolites, is contraindicated for individuals with allergies to these metabolites or a history of severe adverse reactions. 2. This product is contraindicated for use in newborns, infants, and young children. 3. This product is contraindicated for pregnant and lactating women. 4. Individuals with a family history of allergies, personal history of allergic reactions, or an atopic constitution should avoid this product. | 1.Ya'an Sanjiu Pharmaceutical Co., Ltd.  2.Zhengda Qingchunbao Pharmaceutical Co., Ltd.  Dali Pharmaceutical Co., Ltd.  3.Sichuan Chuanda West China Pharmaceutical Co. Ltd.  4.Shineway Pharmaceutical Co. Ltd.  5.Sichuan Province Shenghe Pharmaceutical Co., Ltd.  6.Yunnan Gejiu Biological Pharmaceutical Co. Ltd. | Y-National Pharmaceutical  Standard: Z51021845 |
| Shenfu injection | To prepare the solution, begin by taking an appropriate volume of water for injection and heat it to boiling. Add the effective parts of processed aconite (aconitum) and ginseng to the boiling water while stirring continuously until they are completely dissolved. Adjust the pH of the solution to approximately 3-5 using suitable acid or base as necessary. Boil the adjusted solution again to ensure complete dissolution and sterilization, then refrigerate the liquid for 8-16 hours to allow any insoluble particles to settle. Following refrigeration, filter the solution to remove precipitates or undissolved materials, add activated carbon to the filtrate to adsorb impurities and colorants, and boil this mixture to facilitate impurity removal. Filter the boiled solution through a microporous membrane to obtain a clear, particle-free filtrate. Next, add Tween-80 to the filtrate and stir until it is completely dissolved, aiding in solubilizing any metabolites that may not be fully soluble in water. Adjust the volume to the desired final amount with water for injection, and carefully fill the prepared solution into sterile containers. Finally, perform steam sterilization of the filled containers at 100 °C for 30-60 minutes to ensure sterility of the final product. Throughout the process, ensure all equipment and materials are sterilized before use, conduct quality control checks at each step to confirm purity, pH, and sterility, and follow all relevant safety protocols when handling boiling liquids and during the sterilization process. | Indication: To restore Yang qi and consolidate qi, this therapy is primarily indicated for conditions involving the depletion of Yang qi, such as septic shock, hemorrhagic shock, hypovolemic shock, etc. It can also address Yang deficiency (or qi deficiency) manifested as insomnia, epigastric pain, palpitations, asthmatic cough, diarrhea, and sequelae of poliomyelitis, among others.  Adverse reactions: Occasional adverse reactions of this product are reported:  Allergic reaction: This product can cause a range of allergic reactions, including pruritus, rash, allergic dermatitis, pallor (pale complexion), flatulence, dyspnea, laryngeal edema, palpitations, cyanosis (purple discoloration of the skin or mucous membranes), hypotension (blood pressure drop), and in severe cases, anaphylactic shock. Systemic effects may encompass chills, fever, fatigue, diaphoresis (excessive sweating), and low back pain. Nervous system symptoms can present as dizziness, headache, insomnia, tremors, sobbing, and numbness of the lips and limbs. Cardiovascular manifestations may include facial flushing, palpitations, chest tightness, tachycardia, arrhythmias, and blood pressure fluctuations. Gastrointestinal symptoms may consist of nausea, vomiting, abdominal distension, abdominal pain, diarrhea, hiccups, dry mouth, stomach discomfort, and abnormal liver function tests. Respiratory issues may involve cyanosis of the lips, cough, shortness of breath, and dyspnea. Urinary system complications might include urinary retention and edema. Additional adverse effects can comprise epistaxis (nosebleeds), injection site erythema (redness) and pain, phlebitis, and visual abnormalities. According to active safety monitoring conducted in 31 hospitals in China, the cumulative incidence of adverse reactions/events associated with this product was 0.92%, classified as "rare," with a severity status described as "general," and no "serious" adverse reactions/events were reported.  Contraindications: 1. This product is contraindicated for individuals with a history of allergies or serious adverse reactions to its metabolites. 2. The use of this product is prohibited in newborns, infants, and young children. | 1.Ya'an Sanjiu Pharmaceutical Co., Ltd.  2.Shenzhen Ya'an Pharmaceutical Co., Ltd.  3.Hebei Shineway Pharmaceutical Co. Ltd. | Y-National Pharmaceutical  Standard: Z51020664 |
| Astragalus injection | To prepare the Astragalus membranaceus extract, 2000g of Astragalus membranaceus was decocted with water three separate times, each for 1.5 hours. The resulting decoctions were combined and filtered, with the filtrate being concentrated to yield a solution containing 1-2g of the original medicinal material per 1ml. This filtrate underwent two rounds of ethanol precipitation, initially with a 75% ethanol solution and subsequently with an 85% ethanol solution, with each round followed by cold storage to precipitate impurities. The solution was then diluted with water for injection to achieve a concentration of 0.75 to 1g of the original medicinal material per 1ml and was refrigerated for 12 hours before filtration. Following this, the filtrate was concentrated to contain 5 to 6g of the original medicinal material per 1ml, cooled, and its pH adjusted to 7.5 using a 20% sodium hydroxide solution. The mixture was then boiled, and 0.125% activated carbon was added, followed by boiling for 5 minutes. While still hot, the solution was filtered, and water for injection was added to adjust the volume to 1000ml. The pH was once again adjusted to 7.5 with a 20% sodium hydroxide solution, after which the solution was filtered, filled into sterile containers, and sterilized to obtain the final product. | Indication: Nourishing qi, nourishing and eliminating pathogenic factors, nourishing the heart and tonifying the spleen and dampness. It is indicated for viral myocarditis associated with heart-qi deficiency and blood stasis, as well as for cardiac insufficiency. Furthermore, it addresses hepatitis caused by spleen deficiency and dampness. By supporting these aspects of health, the preparation seeks to restore balance and promote healing in affected individuals.  Adverse reactions: 1. Systemic damage: anaphylactoid reactions, anaphylactic shock, chills, fever, pallor, etc. 2. Respiratory system: dyspnea, cyanosis, asthma, cough. 3. Cardiovascular system: palpitations, chest tightness. 4. Digestive system: nausea and vomiting. 5. Skin and its accessories: sweating, rash, itching. 6. Nervous system: dizziness, headache.  Contraindications: 1. It is forbidden to use this product or astragalus containing preparations with a history of allergy or serious adverse reactions. 2. This product contains polysorbate 80, which is contraindicated for those allergic to polysorbate 80. 3. Pregnant women and infants are prohibited. 4. This product is warm and nourish the product, there is heat, the surface is full of evil,It is contraindicated when Yang is hyperactive, when the ulcer is ulcerated or when the heat and toxin are still excessive, and when the heart and liver are hot and the spleen and stomach are damp and hot. | 1.Shijiazhuang Luancheng Sunway Pharmaceutical Co., Ltd  2.Chengdu Diao Jiuhong Pharmaceutical Co., Ltd. | Y-National Pharmaceutical  Standard: Z51021775 |

**Supplementary table S3** Analytical methods for the chemical profile of the four kinds of TCMI-TQs according to ConPhyMP

| TCMI-TQs | Type of extract | Preferred/main methods and metabolite identification results for extract characterisation/chemical analysis | Alternative methods and metabolite identification results for extract characterisation/chemical analysis | Reference |
| --- | --- | --- | --- | --- |
| Shengmai injection | A | Determining content using high-performance liquid chromatography  Chromatographic conditions and system suitability test  The octadecylsilane bonded silica gel was used as filler. The mobile phase was acetonitrile-0.05% phosphoric acid solution (19:81). The detection wavelength was 203nm. The number of theoretical plates was calculated according to the ginsenoside Re peak, which should not be less than 4000.  Preparation of the control solution  Precision weighing ginsenoside Rg_1_15mg and ginsenoside Re 12mg, which were dried to a constant weight in a phosphorus pendant desiccator, placed in a 25ml volumetric flask, added acetonitrin-water (19:81) to the scale, and shaken well. Precision taking 5ml, placed in a 10ml volumetric flask, added acetonitrin-water (19:81) diluted to the scale, and shaken well. Instant (ginsenoside Rg_1_0.30mg/1ml, Re0.24mg).  Preparation of the test solution  Take 10ml of this product, steam to near dry, add mobile phase to dissolve, and dilute to 2ml, as the test solution. Method of determination  The control solution and the test solution of 20μl each were precisely aspirated, respectively, and injected into the liquid chromatograph for determination.  This product should contain no less than 0.08mg ginsenoside Rg1 and no less than 0.04mg ginsenoside Re per 1ml. | (1) Take about 10 ml of this product, dry on a water bath, the residue with 2ml of ethanol to dissolve, as the test solution. Ginsenosides Rb1, Re and Rg_1_ were added to make a mixed solution containing 2mg per 1ml of ethanol, which was used as a control solution. According to the TLC test, 2~4μl of each solution was sucked on the same silica gel G thin layer plate, with chloroform-methanol-water (75:20:2) as the development agent, expanded, removed, dried, sprayed with 10% ethanol sulfate solution, dried at 105℃ for a few minutes, and placed under ultraviolet light (365nm) for inspection. In the chromatogram of the test substance, fluorescent spots of the same color appear at the positions corresponding to the chromatogram of the control substance.  (2) Take 40ml of this product, add 3ml of hydrochloric acid, heat it in a water bath for 1 hour, let it cool, add 30ml of diethyl ether to shake and extract, separate the diethyl ether solution, dry, and add 1ml of chloroform to dissolve the residue, as the test solution. Another 2g of the control medicinal materials of Ophiopogon japonicus was decocted with water for 30 minutes, filtered, and the filtrate was concentrated to about 40ml. The control medicinal materials solution was prepared by the same method. According to the TLC test, 5 ~ 10μl of the above two solutions were sucked, respectively, on the same silica gel G thin layer plate, with chloroform and acetone (4:1) as the development agent, expanded, removed, dried, sprayed with 10% ethanol sulfate solution, and dried at 105℃ for about 5 minutes. In the chromatogram of the test substance, spots of the same color were shown at the corresponding positions of the chromatogram of the control medicinal substance.  (3) Take 50ml of this product, concentrate to 25ml on a water bath, move to a separating funnel, add chloroform and shake to extract 3 times, 10ml each time, filter, combine the filtrate, steam to dry, add 1ml chloroform to dissolve the residue, as the test solution. The control substance Schisandrin A was added with chloroform to make a solution containing 0.5mg per 1ml, which was used as the control substance solution. According to the thin-layer chromatography test, 4-5 μl of the test solution and 1-2 μl of the control solution were sucked on the same silica gel GF254 thin-layer plate, respectively. The upper solution of petroleum ether (30-60 ℃) -ethyl formate-formic acid (14:5:1) was used as the development agent, which was developed, removed, dried, and examined under ultraviolet light (254nm). In the chromatogram of the test substance, spots of the same color appear at positions corresponding to the chromatogram of the control substance. | National Drug Standards of China Food and Drug Administration (WS3-B-2865-98-2011) |
| Shenmai injection | A | Preparation of the control solution  The reference substance of ginsenoside Re was precisely weighed and dried to a constant weight at 60℃ under reduced pressure, dissolved in methanol, and quantitatively diluted to make a solution containing ginsenoside Re 2mg per 1ml.  Preparation of standard curves  Precision take the control solution 10, 20, 40, 60, 80, 100μl, respectively, put in a 10ml plug test tube, melt the solvent, precision add 5% vanillin glacial acetic acid solution-perchloric acid (2:8) (new preparation) mixed solution 1ml, placed in a 60℃ water bath for 15 minutes, take out, put in an ice bath cooling, precision add glacial acetic acid 5ml, The absorbance was measured immediately by spectrophotometry at a wavelength of 544nm. The absorbance was used as the ordinate and the concentration as the abscissa to draw a standard curve.  Method of determination  Precision take 1ml of this product, through the pretreatment of macroporous resin (D101 column 1.5×12cm), the first water 25ml elution, discard the liquid water. Then, the eluate was eluted with 60ml of 75% ethanol, collected, evaporated to dryness, dissolved in ethanol and quantitatively transferred to a 10ml volumetric flask, diluted to the scale with ethanol, shaken well, as the test solution. A precise amount of 1ml was taken into a stopper test tube, evaporated to dryness, according to the method under the preparation of the standard curve. From "precisely add 5% vanillin glacial acetic acid solution - perchloric acid (2:8) mixed solution 1ml", according to the law to determine the absorbance, read out the concentration of the test solution from the standard curve, calculate, then get.  The total saponins in each 1ml of this product shall not be less than 0.80mg based on ginsenoside Re(C48H82O18). | (1) Take 1ml of this product, put in the evaporating dish, dry on the water bath, add 0.5ml of acetic anhydride to dissolve, move into the test tube, add 0.5ml of sulfuric acid along the test tube wall, the interface of the two liquid is brown red ring.  (2) Take 1ml of this product, put it in a test tube, add 3 drops of the newly prepared α-naphthol ethanol solution (1→10), mix, and then add 0.5ml of sulfuric acid along the test tube wall, the interface of the two liquid is purple red ring.  (3) Take 5ml of this product, dry on water bath, add 1ml of ethanol to dissolve, as the test solution. Ginsenosides Rb1, Rg_1_, Re were added to make a mixed solution containing 2mg per 1ml of ethanol, which was used as a control solution. According to the TLC test, 2~5μl of each solution was sucked, respectively, on the same silica gel G thin layer plate, with chloroform - methanol - water (13:7:2) as the development agent, expanded, removed, dried, sprayed with ethanol solution of phospomolybdic acid (1→10) and heated at 120℃ for 5 minutes. In the chromatogram of the test substance, three spots of the same color were shown at the corresponding position of the chromatogram of the control substance. | National Drug Standards of China Food and Drug Administration (WS3-B-3428-98-2010Z) |
| Shenfu injection | A | Preparation of the control solution  Reference ginsenoside Rb1 was precisely weighed and a solution containing 3mg ginsenoside Rb1 per 1ml of methanol was prepared to be obtained.  Preparation of standard curves  Precision suction control solution 20, 30, 40, 50, 60μl, respectively, placed in a plug test tube, the solvent on the water bath, cooled, each precision added 5% vanillin glacial acetic acid solution 0.2ml, perchloric acid 0.8ml, shake well, placed in 60℃ water bath for 15 minutes, take out, cool in ice water bath, add glacial acetic acid 5ml, shake well, The absorbance was measured at a wavelength of 550nm by spectrophotometry with the corresponding reagent as blank. The absorbance was used as the ordinate and the concentration as the abscissa to draw the standard curve.  Method of determination  10ml of this product was precisely absorbed, placed in a separating funnel, extracted 4 times with chloroform shaking, 10ml each time, discarded the chloroform layer, the water layer was extracted 4 times with water-saturated n-butyl alcohol, 10ml each time, combined with n-butyl alcohol liquid, and then washed 2 times with n-butyl alcohol saturated water, 10ml each time, discarded water liquid, n-butyl alcohol liquid was placed on a water bath to dry. The residue was dissolved with methanol, quantitatively transferred to a 5ml volumetric flask, and diluted to scale, shaken, and ready to be obtained. In addition, 10ml of 0.2% (g/ml) polyssorbate 80 solution was used as blank control solution. Precisely absorb 50μl of the above test solution, place it in a stopper test tube, and determine the absorbance according to the method under the standard cold curve preparation "remove the solvent from the water bath", and then calculate it.  Each 1ml of this product contains ginsenoside Rb1(C54H92O23), not less than 0.5mg. | (1) Take 1ml of this product, put it in the evaporating dish, dry on the water bath, add 0.5ml of acetic anhydride to the residue, dissolve it, move into the test tube, add 0.5ml of sulfuric acid along the test tube wall, the interface of the two liquid is brown red ring.  (2) Take 30ml of this product, put in the separating funnel, add 30ml of chloroform, shake, stand, take the upper liquid to dry, the residue with 2ml of water to dissolve, add 10ml of water-saturated n-butyl alcohol, ultrasonic treatment for 30 minutes, absorb the supernatant, add three times the amount of ammonia test solution, shake well, place layered, take the upper liquid to dry, the residue with 1ml of methanol to dissolve, as the test solution. In addition, 1g of red ginseng control medicine was taken, 30ml of ethanol was added, refluxated on the water bath for 30 minutes, filtered, the filtrate was dried, the residue was dissolved with 30ml of water, filtered, and the filtrate was made into the control medicine solution by the same method. Then ginsenoside Rb1, Re and Rg1 were mixed with methanol to make a solution containing 2mg per 1ml, which was used as a control solution. According to the thin-layer chromatography test, 10μl of the test solution, 20μl of the control medicinal material solution and 2μl of the control solution were sucked on the same silica gel G thin layer plate, and the lower layer solution placed below 10 ° C in chloroform, ethyl acetate-methanol-water (15:40:22:10) was used as the development agent. The solution was expanded, removed, dried, and sprayed with 10% ethanol sulfate solution. They were dried at 105 ° C until the spots showed clear color. In the chromatogram of the test substance, spots of the same color were observed at the positions corresponding to the chromatogram of the control medicinal material, and spots of the same color were observed at the positions corresponding to the chromatogram of the control substance. | National Drug Standards of China Food and Drug Administration (WS3-B-3427-98) |
| Astragalus injection | A | Take 10ml of this product, put on a water bath to dry, the residue plus 1% sodium hydroxide solution 2ml to dissolve, through D101 type macroporous adsorption resin column (inner diameter 1cm, length 12cm), with 1% sodium hydroxide solution 50ml elution, discard the eluent, water elution to neutral (about water 50ml), discard the water liquid, and then with 50ml 30% ethanol elution. The eluate was discarded and then eluted with 50mL 70% ethanol. The eluate was collected, evaporated to dry, dissolved in methanol and transferred to a 2ml volumetric flask, diluted to the scale with methanol, shaken well, and used as the test solution. Another astragaloside Ⅳ control substance was added to methanol to make a solution containing 1mg per 1ml, which was used as a control substance solution. According to the thin-layer chromatography test, the test solution 5μl, the control solution 2 μl and 4 μl, respectively, cross the same silica gel G thin layer plate, with chloroform - ethyl acetate - methanol - water (15:40:22:10) placed below 10℃ as the development agent, expanded, removed, dried, sprayed with 20% sulfuric acid ethanol solution, and the solution of the lower layer was used as the development agent. They were dried at 105 °C until the spots showed clear color, removed, and covered with a glass plate of the same size on a thin plate. Secure the perimeter with tape. Scanning according to thin layer chromatography, wavelength: λs= 395nm, λR= 700nm, measure the integral value of the absorbance of the test substance and the integral value of the absorbance of the reference substance, and calculate, then obtain.  Each 1ml of this product contains astragaloside IV (C41H68O14) not less than 0.08mg. | Take 1.5ml of this product, add water to 30ml, extract 2 times with water-saturated n-butyl alcohol, 20ml each time, combine n-butyl alcohol liquid, wash 2 times with water, 20nl each time, discard the water liquid, n-butyl alcohol liquid is placed on a water bath to dry, and the residue is dissolved with 0.5ml methanol, as the test solution. The control astragaloside Ⅳ was added to methanol to make a solution containing 1mg per 1ml, which was used as the control solution. According to the TLC test, 2μl of the above two solutions were sucked on the same silica gel G thin layer plate, and the lower layer solution of chloroform, methanol and water (13:7:2) placed under 10℃ was used as the development agent. The solution was expanded, removed, dried, sprayed with 10% sulfuric acid ethanol solution, and dried at 105℃ until the spots showed clear color. They were examined under sunlight and ultraviolet light (365nm). In the chromatogram of the test substance, the same tan spots under sunlight and the same orange yellow fluorescent spots under ultraviolet light (365nm) were observed at the corresponding positions of the control substance. | National Drug Standards of China Food and Drug Administration (WS3-B-3335-98) |

**Supplementary table S4** The principal chemical metabolite s of four TCMI-TQs under fingerprint methods.

| TCMI -TQs | Literature sources | fingerprinting methods | chemical metabolites |
| --- | --- | --- | --- |
| Shengmai injection | (Zheng et al., 2012) | HPLC | Ginsenoside Rg1, Ginsenoside Re, Ginsenoside Rf, Ginsenoside Rb1, Ginsenoside Rc, Ginsenoside Rg2, Ginsenoside Rb2, Ginsenoside Rb3, Ginsenoside Rd, Schizandrol A, 20(S)－protopanaxadiol, Schisantherin A, Deoxyschizandrin, Schisandrin B |
| Shenmai injection | (Li et al., 2022) | ^1^H-NMR | Isoleucine, Valine, Alanine, Glutamate, Glutamine, Pyroglutamic acid, Proline, Pyruvate, Malic acid, Succinic acid, Malonic acid, γ-aminobutyric acid, Lactic acid, Acetic acid, Formic acid, Choline, Glucose, Fructose, Sucrose, Maltose, Adenosine, Uridine, Ginsenoside Rb1, ginsenoside Rb2, ginsenoside Rc, ginsenoside Rd, ginsenoside Re, ginsenoside Rf, ginsenoside Rg1, polysorbate 80, ethanol, acetone |
|  | (Yang et al., 2019) | UHPLC | Ginsenosides Rg1, Ginsenosides Re, Ginsenosides Rf, Ginsenosides Rb1, Ginsenosides Rc, Ginsenosides Rb2, Ginsenosides Rb3, Ginsenosides Rd, 20(S)-ginsenosides Rh1, 20(S)-ginsenosides Rg2, 20(S)-ginsenosides Rg3 |
|  | (Zhu et al., 2023) | HPLC-MS/MS | Bornenol-7-O-[β-D-apiofuranosyl-(1→6)]-β-D-glucopyranoside, Borneol-7-O-β-D-Glucopyranoside |
| Shenfu injection | (He et al., 2014) | UPLC-Q-TOF/MS | 20glucosylginsenpside Rf, Notoginsenoside R1, Ginsenoside Rg_1_, Ginsenoside Rf, Ginsenoside F3, Ginsenoside Ra_2_, Ginsenoside Rh1, Ginsenoside F1, Ginsenoside Rb_1_, Ginsenoside Re, Ginsenoside Rd, Ginsenoside Ro, Ginsenoside Rc, Ginsenoside Ra_1_, Ginsenoside Rb_2_, Chikusetsusaponin Iva, Mesaconine, Carmichaeline, Cammaconine, Fuziline, Neoline, Talatizamine, Benzoylaconine, 10-0H-mesaconitine, Benzoylmesaconine, Hypaconitine |
|  | (Liu et al., 2016) | HILIC-LC-MS | Cinnamic acid, Ferulic acid, 4-hydroxylbenzoic acid, L-(+)-lactic acid, Adipic acid, Fumaric acid, Caffeic acid, Succinic acid, Maleic acid, Malonic acid, D-malic acid, (－)-shikimic acid, D-tartaric acid, Quinic acid |
| Astragalus injection | (Yang et al., 2024) | ^1^H-NMR | Isoleucine, Leucine, Valine, Alanine, Lactate, Pyroglutamate, Asparagine, γ-aminobutyric acid, Proline, Formate, Acetate, Fumaric acid, Malic acid, Succinic acid, Malonic acid, Sucrose, Fructose, Lactose, Glucose, Choline, Betaine, Trigonelline, Uridine, Adenosine, Cytidine, Guanosine, Adenine, Astragaloside III, Astragaloside IV, Ononin, Calycosin-7-glucoside, 9,10-dimethoxypterocarpan-3-O-glucoside, Isomucronulatol 7-O-glucoside |
|  | (Dou et al., 2002) | ESI-MS/NMR | Formononetin (1), Calycosin (2), 6"-O-acetyl formononeside (3), Formononeside (7), Red clover isoflavone-7-O-β-D-glucopyranose (12), Calycosin-7-O-β-D-glucopyranose (13), 9, 10-dimethoxypterane-3-O-β-D-glucopyranose (4), 2"-hydroxy-3',4'-dimethoxy-isoflawane-7-O-β-D glucopyranose (6), Acetylastragaloside Ⅰ (5), Astragaloside Ⅰ (8), Isoastragaloside Ⅰ (9), Isoastragaloside Ⅱ (10), Astragaloside Ⅱ (11), Astragaloside Ⅳ (14) |

References

1. Zheng X W, Wang Q, Yu J D, et al. Establishment of HPLC fingerprint of Shengmai injection[J]. Chin J Pharm Anal, 2012, 32(8):1471-1475.
2. Li W Z, Yang J Y, Zhao F, et al. Fingerprint of Shenmai Injection based on 1H-NMR technique. China J. Chin. Mater. Med.,2022,47(3):581-586.
3. Zhu Z W, Xu Q P, Zhang M T, et al. Determination of Bornenol-7-O-[β-D-apiofuranosyl-(1→6)]-β-D-glucopyranoside and Borneol-7-O-β-D-glucopyranoside in Ophiopogonis Radix and Shenmai Injection by HPLC-MS/MS. Chin J Mod Appl Pharm,2023,40(6):792-797.
4. Yang L, Chen J T, Xu X Z, et al. Simultaneous determination of eleven components in Shenmai injection by UHPLC. Chin J Pharm Anal,2019,39(9):1660-1665.
5. He J L, Zhou S S, Ma Z C, et al. The material basis of Shenfu injection was studied using UPLC-Q-TOF/MS. Chin. Pharmacol. Bull.,2014,30(3):429-433.
6. Liu Y, Zhang N, Shi S P, et al. Simultaneous determination of 14 organic acids in Shenfu injection by hydrophilic interaction chromatography-tandem mass spectrometry. China J. Chin. Mater. Med.,2016,41(18):3342-3348.
7. Yang J Y, Xu S J, Zhang Q, et al. Quantitative determination of hydrophilic and hydrophobic components in Astragalus Injection based on 1H-NMR. Chin. Tradit. Herb. Drugs, 2024, 55 (10): 3321-3330.
8. Dou H, Fu T J, Zhang F, et al. The chemical composition of Astragalus injection. Nat. Prod. Res. Dev., 2002, (6): 14-17.

**Supplementary table S5** The details of search terms and literature search strategy

Take searching PubMed as an example, the search terms and strategies are as follows:

| Search | Query |
| --- | --- |
| #1 | Myocardial Infarction[MeSH] |
| #2 | Infarction, Myocardial[TIAB] |
| #3 | Infarctions, Myocardial[TIAB] |
| #4 | Myocardial Infarctions[TIAB] |
| #5 | Cardiovascular Stroke[TIAB] |
| #6 | Cardiovascular Strokes[TIAB] |
| #7 | Stroke, Cardiovascular[TIAB] |
| #8 | Strokes, Cardiovascular[TIAB] |
| #9 | Myocardial Infarct[TIAB] |
| #10 | Infarct, Myocardial[TIAB] |
| #11 | Infarcts, Myocardial[TIAB] |
| #12 | Myocardial Infarcts[TIAB] |
| #13 | Heart Attack[TIAB] |
| #14 | Heart Attacks[TIAB] |
| #15 | Inferior Wall Myocardial Infarction[TIAB] |
| #16 | Diaphragmatic Myocardial Infarction[TIAB] |
| #17 | Diaphragmatic Myocardial Infarctions[TIAB] |
| #18 | Infarction, Diaphragmatic Myocardial[TIAB] |
| #19 | Infarctions, Diaphragmatic Myocardial[TIAB] |
| #20 | Myocardial Infarction, Diaphragmatic[TIAB] |
| #21 | Myocardial Infarctions, Diaphragmatic[TIAB] |
| #22 | Myocardial Infarction, Inferior Wall[TIAB] |
| #23 | Inferior Myocardial Infarction[TIAB] |
| #24 | Infarction, Inferior Myocardial[TIAB] |
| #25 | Infarctions, Inferior Myocardial[TIAB] |
| #26 | Inferior Myocardial Infarctions[TIAB] |
| #27 | Myocardial Infarction, Inferior[TIAB] |
| #28 | Myocardial Infarctions, Inferior[TIAB] |
| #29 | Acute Inferior Myocardial Infarction[TIAB] |
| #30 | [Anterior Wall Myocardial Infarction](https://www.ncbi.nlm.nih.gov/mesh/68056988)[TIAB] |
| #31 | Myocardial Infarction, Anterior Wall[TIAB] |
| #32 | Anterolateral Myocardial Infarction[TIAB] |
| #33 | Anterolateral Myocardial Infarctions[TIAB] |
| #34 | Infarction, Anterolateral Myocardial[TIAB] |
| #35 | Infarctions, Anterolateral Myocardial[TIAB] |
| #36 | Myocardial Infarction, Anterolateral[TIAB] |
| #37 | Myocardial Infarctions, Anterolateral[TIAB] |
| #38 | Anteroseptal Myocardial Infarction[TIAB] |
| #39 | Anteroseptal Myocardial Infarctions[TIAB] |
| #40 | Infarction, Anteroseptal Myocardial[TIAB] |
| #41 | Infarctions, Anteroseptal Myocardial[TIAB] |
| #42 | Myocardial Infarction, Anteroseptal[TIAB] |
| #43 | Myocardial Infarctions, Anteroseptal[TIAB] |
| #44 | Acute Anterior Wall Myocardial Infarction[TIAB] |
| #45 | Non-ST Elevated Myocardial Infarction[TIAB] |
| #46 | Non ST Elevated Myocardial Infarction[TIAB] |
| #47 | NSTEMI[TIAB] |
| #48 | Non-ST-Elevation Myocardial Infarction[TIAB] |
| #49 | Infarction, Non-ST-Elevation Myocardial[TIAB] |
| #50 | Infarctions, Non-ST-Elevation Myocardial[TIAB] |
| #51 | Myocardial Infarction, Non-ST-Elevation[TIAB] |
| #52 | Myocardial Infarctions, Non-ST-Elevation[TIAB] |
| #53 | Non ST Elevation Myocardial Infarction[TIAB] |
| #54 | Non-ST-Elevation Myocardial Infarctions[TIAB] |
| #55 | ST Elevation Myocardial Infarction[TIAB] |
| #56 | ST Segment Elevation Myocardial Infarction[TIAB] |
| #57 | ST Elevated Myocardial Infarction[TIAB] |
| #58 | STEMI[TIAB] |
| #59 | #1-58/OR |
| #60 | Shengmai[TIAB] |
| #61 | Shengmai injection[TIAB] |
| #62 | Sheng Mai Injection[TIAB] |
| #63 | Sheng Mai[TIAB] |
| #64 | #60-63/OR |
| #65 | Shenmai[TIAB] |
| #66 | Shen Mai[TIAB] |
| #67 | ginseng[TIAB] |
| #68 | Shenmai injection[TIAB] |
| #69 | #65-68/OR |
| #70 | Shenfu injection[TIAB] |
| #71 | Shen Fu[TIAB] |
| #72 | #70-71/OR |
| #73 | Astragalus injection[TIAB] |
| #74 | Huangqi[TIAB] |
| #75 | Huang Qi[TIAB] |
| #76 | #73-75/OR |
| #77 | Clinical Trial[Publication Type] |
| #78 | #70-71/OR |
| #79 | #59 AND #64 AND #77 |
| #80 | #59 AND #69 AND #77 |
| #81 | #59 AND #72 AND #77 |
| #82 | #59 AND #76 AND #77 |
| #83 | #79 0R #80 0R #81 0R #82 |

**Supplementary table S6** The details of TCMIs of all the included studies

| **Study** | **Injections** | **Source** | **Raw material** | **Quality control reported? (Y/N)** |
| --- | --- | --- | --- | --- |
| Bai YK, et al. (2002) | Shenmai injection | Ya'an Sanjiu Pharmaceutical Co., Ltd. | *Ginseng Rubra Radix;* *Ophiopogonis Radix* | Not Mentioned |
| Ceng YL, et al. (2005) | Shenfu injection | Not Mentioned | Not Mentioned | Not Mentioned |
| Chen GL, et al. (2021) | Shenmai injection | Zhengda Qingchunbao Pharmaceutical Co., Ltd. | *Ginseng Rubra Radix;* *Ophiopogonis Radix* | Y-Prepared according to Chinese pharmacopeia |
| Chen QT, et al. (2017) | Shengmai injection | Jiangsu Suzhong Pharmaceutical Co., Ltd. | *Ginseng Rubra Radix;* *Ophiopogonis Radix; Schisandrae Chinensis Fructus* | Not Mentioned |
| Chen WJ, et al. (2003) | Shenfu injection | Not Mentioned | *Ginseng Rubra Radix;* *Aconiti Lateralis Radix Praeparata* | Not Mentioned |
| Chen YJ, et al. (2018) | Shenfu injection | Ya'an Sanjiu Pharmaceutical Co., Ltd. | *Ginseng Rubra Radix;* *Aconiti Lateralis Radix Praeparata* | Y-Prepared according to Chinese pharmacopeia |
| Cui JD, et al. (2006) | Shengmai injection | Pharmaceutical Factory, West China Medical University | *Ginseng Rubra Radix;* *Ophiopogonis Radix; Schisandrae Chinensis Fructus* | Not Mentioned |
| Ding L, et al. (2006) | Shengmai injection | Not Mentioned | *Ginseng Rubra Radix;* *Ophiopogonis Radix; Schisandrae Chinensis Fructus* | Not Mentioned |
| Du YK, et al. (2017) | Shenmai injection | Not Mentioned | *Ginseng Rubra Radix;* *Ophiopogonis Radix* | Y-Prepared according to Chinese pharmacopeia |
| Feng JP, et al. (2019) | Shenfu injection | Ya'an Sanjiu Pharmaceutical Co., Ltd. | *Ginseng Rubra Radix;* *Aconiti Lateralis Radix Praeparata* | Y-Prepared according to Chinese pharmacopeia |
| Feng QL, et al. (2011) | Shenfu injection | Shenzhen Ya'an Pharmaceutical Co., Ltd. | *Ginseng Rubra Radix;* *Aconiti Lateralis Radix Praeparata* | Not Mentioned |
| Li GY, et al. (2013) | Shenfu injection | Ya'an Sanjiu Pharmaceutical Co., Ltd. | *Ginseng Rubra Radix;* *Aconiti Lateralis Radix Praeparata* | Not Mentioned |
| Guo S, et al. (2014) | Shenmai injection | Zhengda Qingchunbao Pharmaceutical Co., Ltd. | *Ginseng Rubra Radix;* *Ophiopogonis Radix* | Not Mentioned |
| Guo SP, et al. (1999) | Shenmai injection | Hangzhou Zhengda Qingbao Pharmaceutical Co., Ltd | Not Mentioned | Not Mentioned |
| Han GJ, et al. (2000) | Astragalus injection | Not Mentioned | *Astragali Radix* | Not Mentioned |
| Han GM, et al. (2003) | Shenmai injection | Zhengda Qingchunbao Pharmaceutical Co., Ltd. | *Ginseng Rubra Radix;* *Ophiopogonis Radix* | Not Mentioned |
| Hao LN, et al. (2021) | Shenfu injection | Ya'an Sanjiu Pharmaceutical Co., Ltd. | *Ginseng Rubra Radix;* *Aconiti Lateralis Radix Praeparata* | Y-Prepared according to Chinese pharmacopeia |
| He CF, et al. (2016) | Shenmai injection | Not Mentioned | *Ginseng Rubra Radix;* *Ophiopogonis Radix* | Not Mentioned |
| Huang YW, et al. (2015) | Shenmai injection | Zhengda Qingchunbao Pharmaceutical Co., Ltd. | *Ginseng Rubra Radix;* *Ophiopogonis Radix* | Y-Prepared according to Chinese pharmacopeia |
| Ji F, et al. (2021) | Shenmai injection | Hebei Shineway Pharmaceutical Co. Ltd. | *Ginseng Rubra Radix;* *Ophiopogonis Radix* | Not Mentioned |
| Kang WL, et al. (2017) | Shenfu injection | Ya'an Sanjiu Pharmaceutical Co., Ltd. | Not Mentioned | Y-Prepared according to Chinese pharmacopeia |
| Lan Z, et al. (2021) | Shenfu injection | Not Mentioned | Not Mentioned | Not Mentioned |
| Li DS, et al. (2017) | Shenfu injection | Ya'an Sanjiu Pharmaceutical Co., Ltd. | *Ginseng Rubra Radix;* *Aconiti Lateralis Radix Praeparata* | Y-Prepared according to Chinese pharmacopeia |
| Li N, et al. (2014) | Shenfu injection | Ya'an Sanjiu Pharmaceutical Co., Ltd. | Not Mentioned | Not Mentioned |
| Li QH, et al. (2016) | Shenmai injection | Hebei Shineway Pharmaceutical Co. Ltd. | *Ginseng Rubra Radix;* *Ophiopogonis Radix* | Y-Prepared according to Chinese pharmacopeia |
| Li R, et al. (2017) | Shenfu injection | Ya'an Sanjiu Pharmaceutical Co., Ltd. | *Ginseng Rubra Radix;* *Aconiti Lateralis Radix Praeparata* | Y-Prepared according to Chinese pharmacopeia |
| Li SG, et al. (2016) | Shenfu injection | Ya'an Sanjiu Pharmaceutical Co., Ltd. | *Ginseng Rubra Radix;* *Aconiti Lateralis Radix Praeparata* | Y-Prepared according to Chinese pharmacopeia |
| Li WD, et al. (2015) | Shenfu injection | Not Mentioned | *Ginseng Rubra Radix;* *Aconiti Lateralis Radix Praeparata* | Not Mentioned |
| Li YF, et al. (2015) | Shenfu injection | Not Mentioned | *Ginseng Rubra Radix;* *Aconiti Lateralis Radix Praeparata* | Not Mentioned |
| Li ZE, et al. (2006a) | Shenfu injection | Ya'an Sanjiu Pharmaceutical Co., Ltd. | Not Mentioned | Y-Prepared according to Chinese pharmacopeia |
| Li ZE, et al. (2006b) | Shenfu injection | Ya'an Sanjiu Pharmaceutical Co., Ltd. | Not Mentioned | Y-Prepared according to Chinese pharmacopeia |
| Li ZM, et al. (2010) | Shenfu injection | Ya'an Sanjiu Pharmaceutical Co., Ltd. | *Ginseng Rubra Radix;* *Aconiti Lateralis Radix Praeparata* | Not Mentioned |
| Liang WS, et al. (2006) | Shengmai injection | Not Mentioned | *Ginseng Rubra Radix; Ophiopogonis Radix; Schisandrae Chinensis Fructus* | Not Mentioned |
| Liu BF, et al. (2012) | Shenmai injection | Zhengda Qingchunbao Pharmaceutical Co., Ltd. | Not Mentioned | Not Mentioned |
| Liu GS, et al. (2016) | Shenmai injection | Not Mentioned | Not Mentioned | Not Mentioned |
| Liu LL, et al. (2016) | Shenmai injection | Dali Pharmaceutical Co., Ltd | *Ginseng Rubra Radix;* *Ophiopogonis Radix* | Not Mentioned |
| Liu LL, et al. (2018) | Shenfu injection | Hebei Shineway Pharmaceutical Co. Ltd. | *Ginseng Rubra Radix;* *Aconiti Lateralis Radix Praeparata* | Y-Prepared according to Chinese pharmacopeia |
| Liu SK, et al. (2004) | Shenmai injection | Zhengda Qingchunbao Pharmaceutical Co., Ltd. | *Ginseng Rubra Radix;* *Ophiopogonis Radix* | Not Mentioned |
| Long SE, et al. (2007) | Shenmai injection | Ya'an Sanjiu Pharmaceutical Co., Ltd. | *Ginseng Rubra Radix;* *Ophiopogonis Radix* | Y-Prepared according to Chinese pharmacopeia |
| Lu DX, et al. (2022) | Shengmai injection | Jiangsu Suzhong Pharmaceutical Co., Ltd. | *Ginseng Rubra Radix;* *Ophiopogonis Radix; Schisandrae Chinensis Fructus* | Y-Prepared according to Chinese pharmacopeia |
| Lu JM, et al. (2011) | Shengmai injection | Sichuan Chuanda West China Pharmaceutical Co. Ltd | *Ginseng Rubra Radix;* *Ophiopogonis Radix; Schisandrae Chinensis Fructus* | Y-Prepared according to Chinese pharmacopeia |
| Lu YH, et al. (2009) | Shengmai injection | Jiangsu Suzhong Pharmaceutical Co., Ltd. | *Ginseng Rubra Radix;* *Ophiopogonis Radix; Schisandrae Chinensis Fructus* | Not Mentioned |
| Luo S, et al. (2016) | Shenmai injection | Not Mentioned | *Ginseng Rubra Radix;* *Ophiopogonis Radix* | Not Mentioned |
| Ma XG, et al. (2019) | Shenfu injection | Not Mentioned | *Ginseng Rubra Radix;* *Aconiti Lateralis Radix Praeparata* | Not Mentioned |
| Ma XS, et al. (2022) | Shenfu injection | Ya'an Sanjiu Pharmaceutical Co., Ltd. | *Ginseng Rubra Radix;* *Aconiti Lateralis Radix Praeparata* | Y-Prepared according to Chinese pharmacopeia |
| Meng FS, et al. (2014) | Shenfu injection | Not Mentioned | *Ginseng Rubra Radix;* *Aconiti Lateralis Radix Praeparata* | Not Mentioned |
| Mi ZY, et al. (2009) | Astragalus injection | Shijiazhuang Luancheng Sunway Pharmaceutical Co., Ltd | *Astragali Radix* | Not Mentioned |
| Mo CR, et al. (2002) | Shenfu injection | Ya'an Sanjiu Pharmaceutical Co., Ltd. | *Ginseng Rubra Radix;* *Aconiti Lateralis Radix Praeparata* | Not Mentioned |
| Pei JN, et al. (2019) | Shenfu injection | Ya'an Sanjiu Pharmaceutical Co., Ltd. | *Ginseng Rubra Radix;* *Aconiti Lateralis Radix Praeparata* | Y-Prepared according to Chinese pharmacopeia |
| Qi YW, et al. (2015) | Shenmai injection | Hebei Shineway Pharmaceutical Co. Ltd. | *Ginseng Rubra Radix;* *Ophiopogonis Radix* | Not Mentioned |
| Qi YW, et al. (2015a) | Shenmai injection | Not Mentioned | *Ginseng Rubra Radix;* *Ophiopogonis Radix* | Y-Prepared according to Chinese pharmacopeia |
| Qi YW, et al. (2015b) | Shenmai injection | Hebei Shineway Pharmaceutical Co. Ltd. | *Ginseng Rubra Radix;* *Ophiopogonis Radix* | Y-Prepared according to Chinese pharmacopeia |
| Qu YZ, et al. (2007) | Shenmai injection | Not Mentioned | *Ginseng Rubra Radix;* *Ophiopogonis Radix* | Not Mentioned |
| Shen YX, et al. (2006) | Shenfu injection | Ya'an Sanjiu Pharmaceutical Co., Ltd. | *Ginseng Rubra Radix;* *Aconiti Lateralis Radix Praeparata* | Y-Prepared according to Chinese pharmacopeia |
| Shi BZ, et al. (2018) | Shenmai injection | Hebei Shineway Pharmaceutical Co. Ltd. | *Ginseng Rubra Radix;* *Ophiopogonis Radix* | Y-Prepared according to Chinese pharmacopeia |
| Shi J, et al. (2016) | Shenmai injection | Ya'an Sanjiu Pharmaceutical Co., Ltd. | *Ginseng Rubra Radix;* *Ophiopogonis Radix* | Y-Prepared according to Chinese pharmacopeia |
| Song CJ, et al. (2018) | Shengmai injection | Ya'an Sanjiu Pharmaceutical Co., Ltd. | *Ginseng Rubra Radix;* *Ophiopogonis Radix; Schisandrae Chinensis Fructus* | Y-Prepared according to Chinese pharmacopeia |
| Sun L, et al. (2019) | Shenmai injection | Sichuan Chuanda West China Pharmaceutical Co. Ltd | *Ginseng Rubra Radix;* *Ophiopogonis Radix* | Y-Prepared according to Chinese pharmacopeia |
| Tang FN, et al. (2019) | Shengmai injection | Jiangsu Suzhong Pharmaceutical Co., Ltd. | *Ginseng Rubra Radix;* *Ophiopogonis Radix; Schisandrae Chinensis Fructus* | Not Mentioned |
| Wang AJ, et al. (2021) | Shenmai injection | Zhengda Qingchunbao Pharmaceutical Co., Ltd. | *Ginseng Rubra Radix;* *Ophiopogonis Radix* | Y-Prepared according to Chinese pharmacopeia |
| Wang CL, et al. (2017) | Shenfu injection | Ya'an Sanjiu Pharmaceutical Co., Ltd. | *Ginseng Rubra Radix;* *Aconiti Lateralis Radix Praeparata* | Y-Prepared according to Chinese pharmacopeia |
| Wang GH, et al. (2019) | Shenmai injection | Ya'an Sanjiu Pharmaceutical Co., Ltd. | *Ginseng Rubra Radix;* *Ophiopogonis Radix* | Y-Prepared according to Chinese pharmacopeia |
| Wang H, et al. (2017) | Shenmai injection | Not Mentioned | *Ginseng Rubra Radix;* *Ophiopogonis Radix* | Not Mentioned |
| Wang HY, et al. (2018) | Shenfu injection | Not Mentioned | *Ginseng Rubra Radix;* *Aconiti Lateralis Radix Praeparata* | Y-Prepared according to Chinese pharmacopeia |
| Wang J, et al. (2016) | Shenmai injection | Ya'an Sanjiu Pharmaceutical Co., Ltd. | *Ginseng Rubra Radix;* *Ophiopogonis Radix* | Y-Prepared according to Chinese pharmacopeia |
| Wang JB, et al. (2017) | Shenmai injection | Zhengda Qingchunbao Pharmaceutical Co., Ltd. | *Ginseng Rubra Radix;* *Ophiopogonis Radix* | Y-Prepared according to Chinese pharmacopeia |
| Wang LM, et al. (2019) | Shengmai injection | Sichuan Chuanda West China Pharmaceutical Co. Ltd | *Ginseng Rubra Radix;* *Ophiopogonis Radix; Schisandrae Chinensis Fructus* | Y-Prepared according to Chinese pharmacopeia |
| Wang X, et al. (2010) | Shengmai injection | Sanjiu Pharmaceutical Co., Ltd. | *Ginseng Rubra Radix;* *Ophiopogonis Radix; Schisandrae Chinensis Fructus* | Y-Prepared according to Chinese pharmacopeia |
| Wang XF, et al. (2008) | Shengmai injection | Sanjiu Pharmaceutical Co., Ltd. | *Ginseng Rubra Radix;* *Ophiopogonis Radix; Schisandrae Chinensis Fructus* | Not Mentioned |
| Wang XY, et al. (2019) | Shenmai injection | Shineway Pharmaceutical Co. Ltd. | *Ginseng Rubra Radix;* *Ophiopogonis Radix* | Y-Prepared according to Chinese pharmacopeia |
| Wang YT, et al. (2018) | Shenfu injection | Ya'an Sanjiu Pharmaceutical Co., Ltd. | *Ginseng Rubra Radix;* *Aconiti Lateralis Radix Praeparata* | Y-Prepared according to Chinese pharmacopeia |
| Wei DX, et al. (2001) | Shenmai injection | Not Mentioned | Not Mentioned | Not Mentioned |
| Wen BC, et al. (2014) | Shenfu injection | Ya'an Sanjiu Pharmaceutical Co., Ltd. | *Ginseng Rubra Radix;* *Aconiti Lateralis Radix Praeparata* | Y-Prepared according to Chinese pharmacopeia |
| Wu YF, et al. (2022) | Shenmai injection | Not Mentioned | *Ginseng Rubra Radix;* *Ophiopogonis Radix* | Not Mentioned |
| Wu ZR, et al. (2016) | Shenmai injection | Not Mentioned | *Ginseng Rubra Radix;* *Ophiopogonis Radix* | Y-Prepared according to Chinese pharmacopeia |
| Xian W, et al. (2019) | Astragalus injection | Chengdu Diao Jiuhong Pharmaceutical Co., Ltd. | *Astragali Radix* | Y-Prepared according to Chinese pharmacopeia |
| Xie H, et al. (2018) | Shenmai injection | Sichuan Province Shenghe Pharmaceutical Co., Ltd | *Ginseng Rubra Radix;* *Ophiopogonis Radix* | Y-Prepared according to Chinese pharmacopeia |
| Xu K, et al. (2022) | Shengmai injection | Made in Yibin, Sichuan | *Ginseng Rubra Radix;* *Ophiopogonis Radix; Schisandrae Chinensis Fructus* | Y-Prepared according to Chinese pharmacopeia |
| Xu XJ, et al. (2018) | Shenmai injection | Zhengda Qingchunbao Pharmaceutical Co., Ltd. | *Ginseng Rubra Radix;* *Ophiopogonis Radix* | Y-Prepared according to Chinese pharmacopeia |
| Xuan JJ, et al. (2015) | Shenmai injection | Zhengda Qingchunbao Pharmaceutical Co., Ltd. | *Ginseng Rubra Radix;* *Ophiopogonis Radix* | Y-Prepared according to Chinese pharmacopeia |
| Yan H, et al. (2017) | Shenfu injection | Ya'an Sanjiu Pharmaceutical Co., Ltd. | *Ginseng Rubra Radix;* *Aconiti Lateralis Radix Praeparata* | Y-Prepared according to Chinese pharmacopeia |
| Yan J, et al. (2018) | Shenmai injection | Yunnan Gejiu Biological Pharmaceutical Co. Ltd | *Ginseng Rubra Radix;* *Ophiopogonis Radix* | Y-Prepared according to Chinese pharmacopeia |
| Yan P, et al. (2018) | Shenmai injection | Zhengda Qingchunbao Pharmaceutical Co., Ltd. | *Ginseng Rubra Radix;* *Ophiopogonis Radix* | Y-Prepared according to Chinese pharmacopeia |
| Yang JW, et al. (2017) | Shenmai injection | Ya'an Sanjiu Pharmaceutical Co., Ltd. | *Ginseng Rubra Radix;* *Ophiopogonis Radix* | Y-Prepared according to Chinese pharmacopeia |
| Yang SQ, et al. (2014) | Shenfu injection | Not Mentioned | *Ginseng Rubra Radix;* *Aconiti Lateralis Radix Praeparata* | Not Mentioned |
| Yuan XH, et al. (2009) | Shenmai injection | Not Mentioned | *Ginseng Rubra Radix;* *Ophiopogonis Radix* | Not Mentioned |
| Yang XZ, et al. (2014) | Shenmai injection | Sichuan Chuanda West China Pharmaceutical Co. Ltd | *Ginseng Rubra Radix;* *Ophiopogonis Radix* | Y-Prepared according to Chinese pharmacopeia |
| Yang ZQ, et al. (2016) | Shengmai injection | Not Mentioned | *Ginseng Rubra Radix;* *Ophiopogonis Radix* | Not Mentioned |
| Ye DJ, et al. (2010) | Shenmai injection | Not Mentioned | *Ginseng Rubra Radix;* *Ophiopogonis Radix* | Not Mentioned |
| Yu HB, et al. (2010) | Shenmai injection | Ya'an Sanjiu Pharmaceutical Co., Ltd. | *Ginseng Rubra Radix;* *Ophiopogonis Radix* | Y-Prepared according to Chinese pharmacopeia |
| Yu XP, et al. (2021) | Shenmai injection | Dali Pharmaceutical Co., Ltd | *Ginseng Rubra Radix;* *Ophiopogonis Radix* | Y-Prepared according to Chinese pharmacopeia |
| Zhan DS, et al. (2023) | Shenmai injection | Ya'an Sanjiu Pharmaceutical Co., Ltd. | *Ginseng Rubra Radix;* *Ophiopogonis Radix* | Not Mentioned |
| Zhang D, et al. (2019) | Shenmai injection | Zhengda Qingchunbao Pharmaceutical Co., Ltd. | *Ginseng Rubra Radix;* *Ophiopogonis Radix* | Y-Prepared according to Chinese pharmacopeia |
| Zhang DL, et al. (2018) | Shenmai injection | Dali Pharmaceutical Co., Ltd | *Ginseng Rubra Radix;* *Ophiopogonis Radix* | Y-Prepared according to Chinese pharmacopeia |
| Zhang DM, et al. (2018) | Shenfu injection | Ya'an Sanjiu Pharmaceutical Co., Ltd. | *Ginseng Rubra Radix;* *Aconiti Lateralis Radix Praeparata* | Y-Prepared according to Chinese pharmacopeia |
| Zhang HX, et al. (2011) | Shenfu injection | Not Mentioned | *Ginseng Rubra Radix;* *Aconiti Lateralis Radix Praeparata* | Not Mentioned |
| Zhang L, et al. (2023) | Shenfu injection | Ya'an Sanjiu Pharmaceutical Co., Ltd. | *Ginseng Rubra Radix;* *Aconiti Lateralis Radix Praeparata* | Y-Prepared according to Chinese pharmacopeia |
| Zhang M, et al. (2011) | Shenmai injection | Not Mentioned | *Ginseng Rubra Radix;* *Ophiopogonis Radix* | Not Mentioned |
| Zhang YH, et al. (2019) | Shenfu injection | Ya'an Sanjiu Pharmaceutical Co., Ltd. | *Ginseng Rubra Radix;* *Aconiti Lateralis Radix Praeparata* | Y-Prepared according to Chinese pharmacopeia |
| Zhang ZL, et al. (2017) | Shenmai injection | Hebei Shineway Pharmaceutical Co. Ltd. | *Ginseng Rubra Radix;* *Ophiopogonis Radix* | Y-Prepared according to Chinese pharmacopeia |
| Zhao CH, et al. (2020) | Shenmai injection | Zhengda Qingchunbao Pharmaceutical Co., Ltd. | *Ginseng Rubra Radix;* *Ophiopogonis Radix* | Y-Prepared according to Chinese pharmacopeia |
| Zhao J, et al. (2016) | Shenfu injection | Not Mentioned | *Ginseng Rubra Radix;* *Aconiti Lateralis Radix Praeparata* | Y-Prepared according to Chinese pharmacopeia |
| Zhao LF, et al. (2021) | Shenmai injection | Sichuan Chuanda West China Pharmaceutical Co. Ltd | *Ginseng Rubra Radix;* *Ophiopogonis Radix* | Y-Prepared according to Chinese pharmacopeia |
| Zhao XP, et al. (2005) | Shenmai injection | Not Mentioned | Not Mentioned | Not Mentioned |
| Zhao Y, et al. (2016) | Shenmai injection | Zhengda Qingchunbao Pharmaceutical Co., Ltd. | *Ginseng Rubra Radix;* *Ophiopogonis Radix* | Y-Prepared according to Chinese pharmacopeia |
| Zheng Y, et al. (2016) | Shenmai injection | Ya'an Sanjiu Pharmaceutical Co., Ltd. | *Ginseng Rubra Radix;* *Ophiopogonis Radix* | Y-Prepared according to Chinese pharmacopeia |
| Zhou RS, et al. (2017) | Shenmai injection | Not Mentioned | *Ginseng Rubra Radix;* *Ophiopogonis Radix* | Not Mentioned |
| Zhou SJ, et al. (2024) | Shenmai injection | Not Mentioned | *Ginseng Rubra Radix;* *Ophiopogonis Radix* | Y-Prepared according to Chinese pharmacopeia |
| Zhu CZ, et al. (2019) | Shenfu injection | Not Mentioned | *Ginseng Rubra Radix;* *Aconiti Lateralis Radix Praeparata* | Not Mentioned |
| Zhu GJ, et al. (2006) | Shenfu injection | Ya'an Sanjiu Pharmaceutical Co., Ltd. | *Ginseng Rubra Radix;* *Aconiti Lateralis Radix Praeparata* | Not Mentioned |
| Zhu XH, et al. (2020) | Shenfu injection | Ya'an Sanjiu Pharmaceutical Co., Ltd. | *Ginseng Rubra Radix;* *Aconiti Lateralis Radix Praeparata* | Not Mentioned |
| Zong XL, et al. (2014) | Shenmai injection | Hebei Shineway Pharmaceutical Co. Ltd. | *Ginseng Rubra Radix;* *Ophiopogonis Radix* | Y-Prepared according to Chinese pharmacopeia |
| Zou JF, et al. (2014) | Shenmai injection | Not Mentioned | *Ginseng Rubra Radix;* *Ophiopogonis Radix* | Not Mentioned |
| Wang X, et al. (2021) | Shenfu injection | Ya'an Sanjiu Pharmaceutical Co., Ltd. | *Ginseng Rubra Radix;* *Aconiti Lateralis Radix Praeparata* | Y-Prepared according to Chinese pharmacopeia |


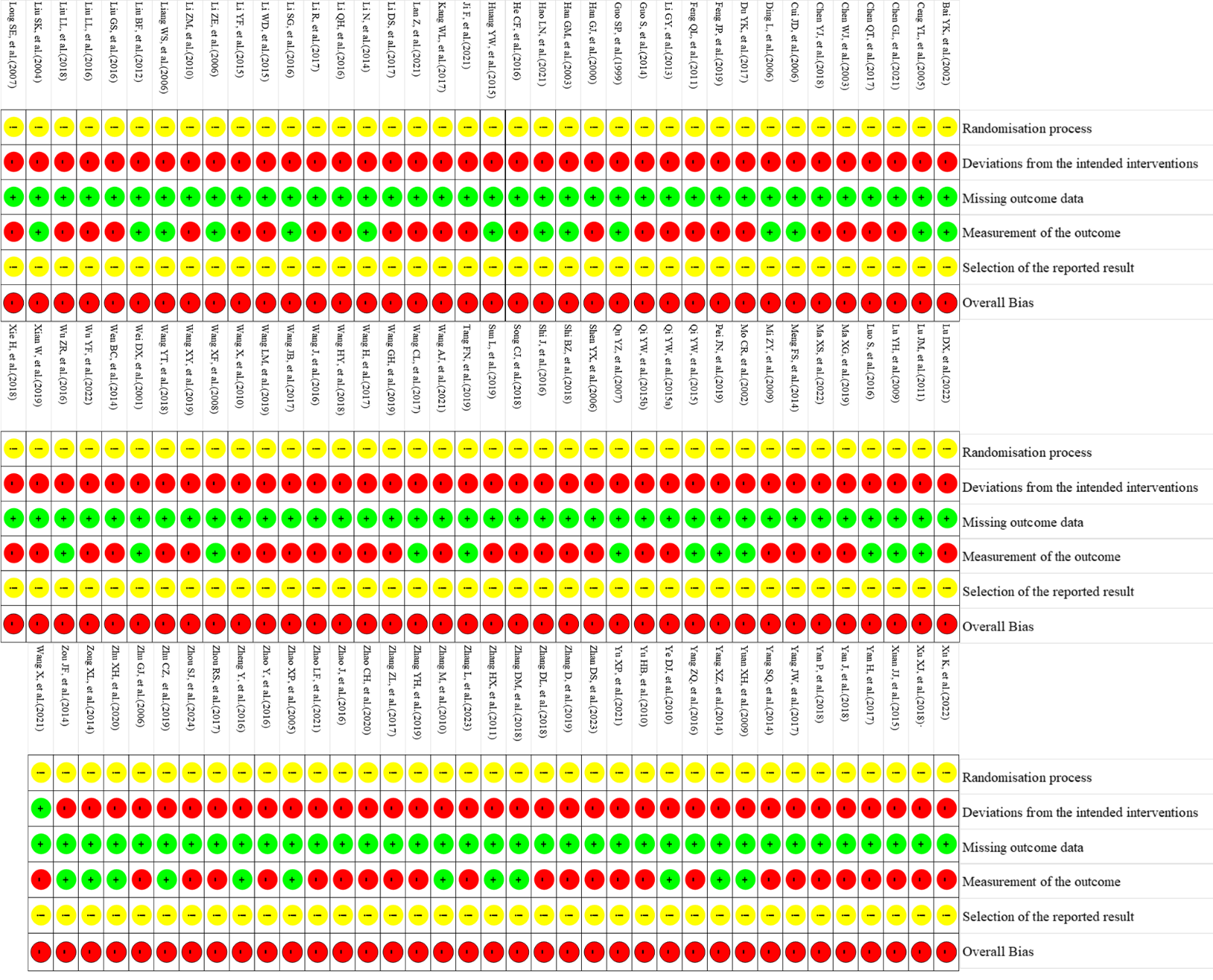
**Supplementary figure F1** Summary of results from assessment of studies using the Cochrane risk of bias tool.


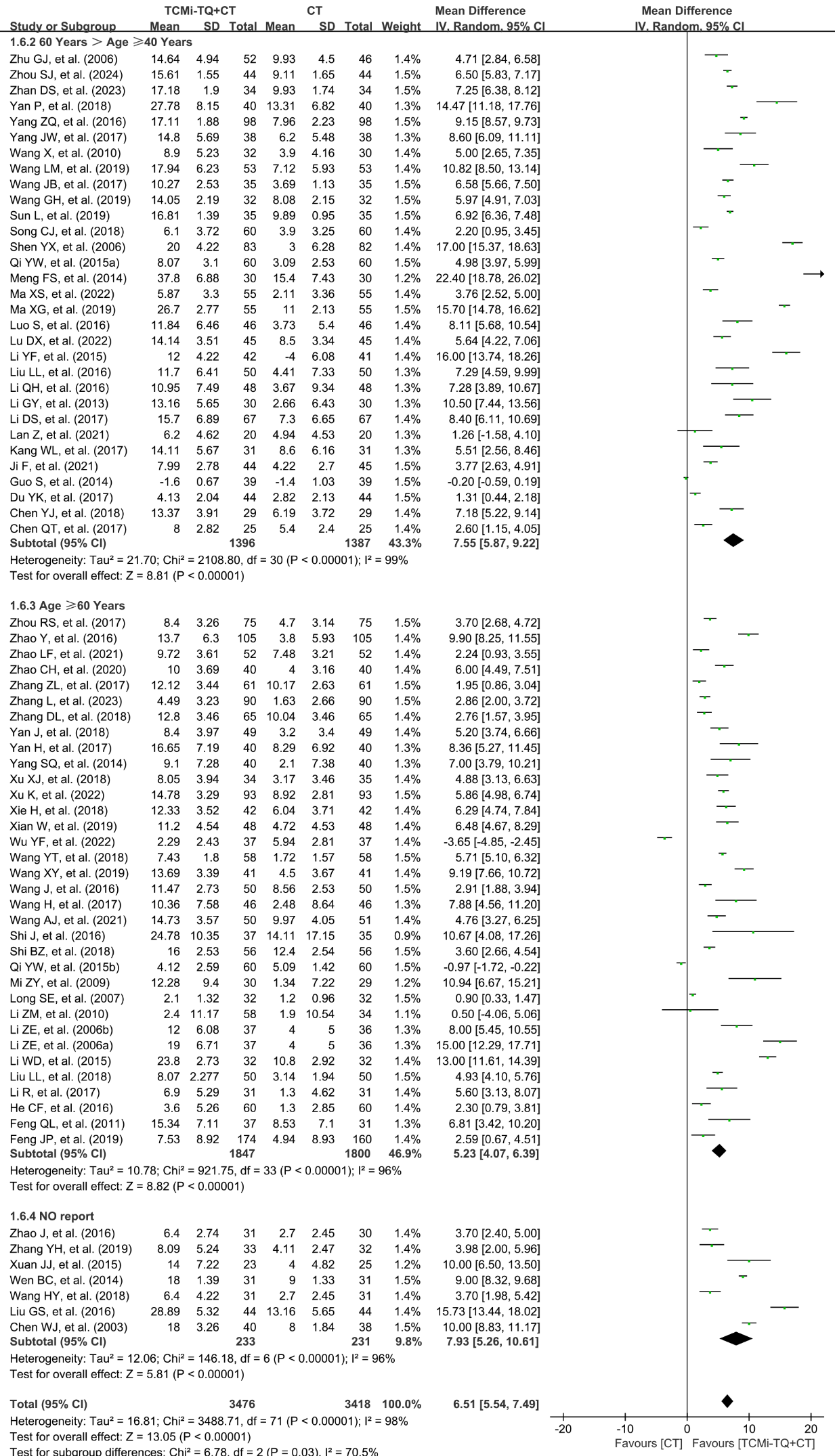


**Supplementary figure F2** Subgroup analysis of the impact of TCMi-TQ combined with conventional treatment on LVEF in AMI patients (based on age). Notes: CI, confidence interval; TCMi-TQ, Traditional Chinese medicine injections for Tonifying Qi; CT, conventional treatment.

**
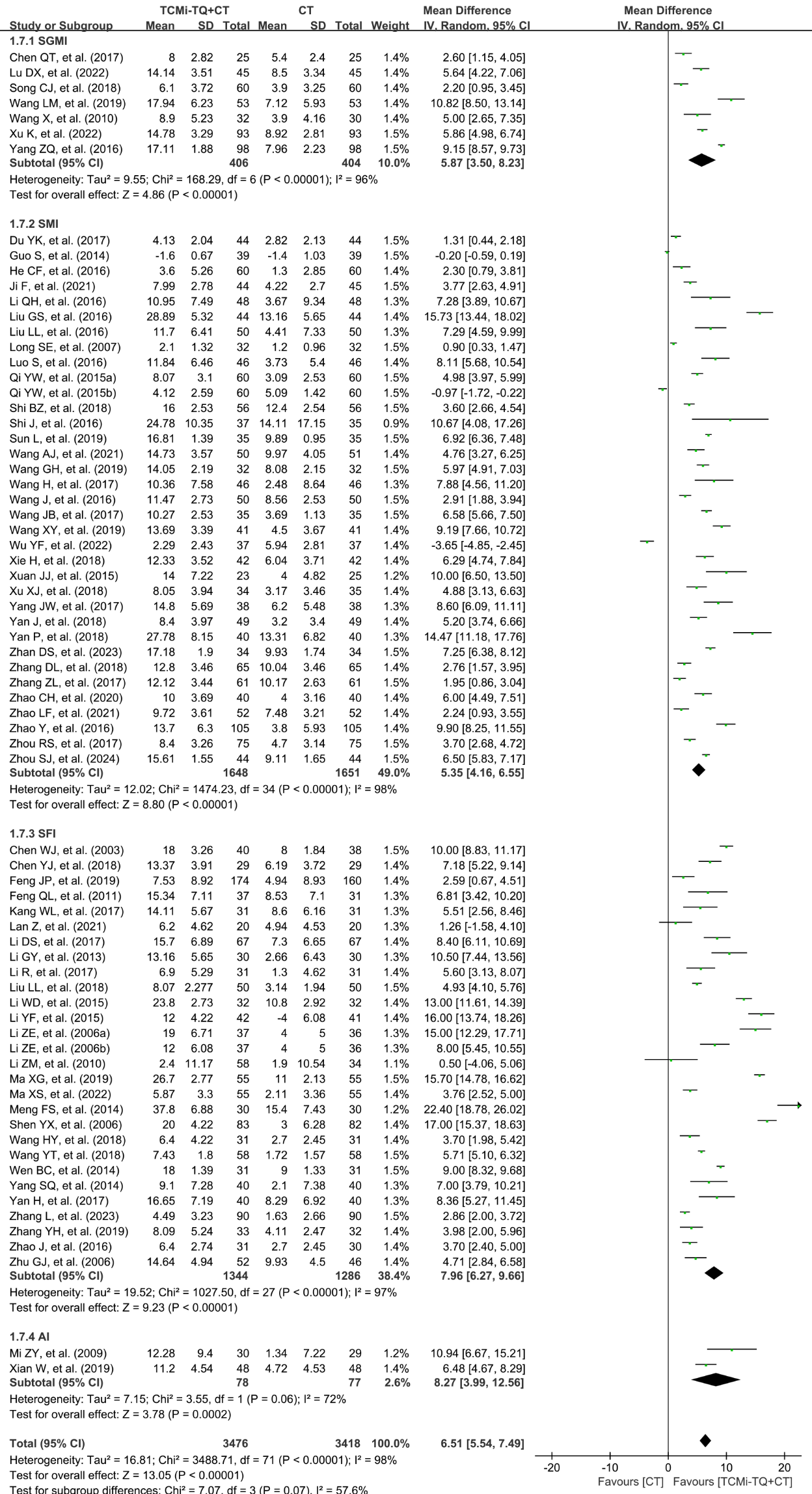
Supplementary figure F3** Subgroup analysis of the impact of TCMi-TQ combined with conventional treatment on LVEF in AMI patients (based on the kind of Traditional Chinese Medicine injections). Notes: CI, confidence interval; TCMi-TQ, Traditional Chinese medicine injections for Tonifying Qi; CT, conventional treatment; SGMI, Shengmai injection; SMI, Shenmai injection; SFI, Shenfu injection; AI, Astragalus injection.


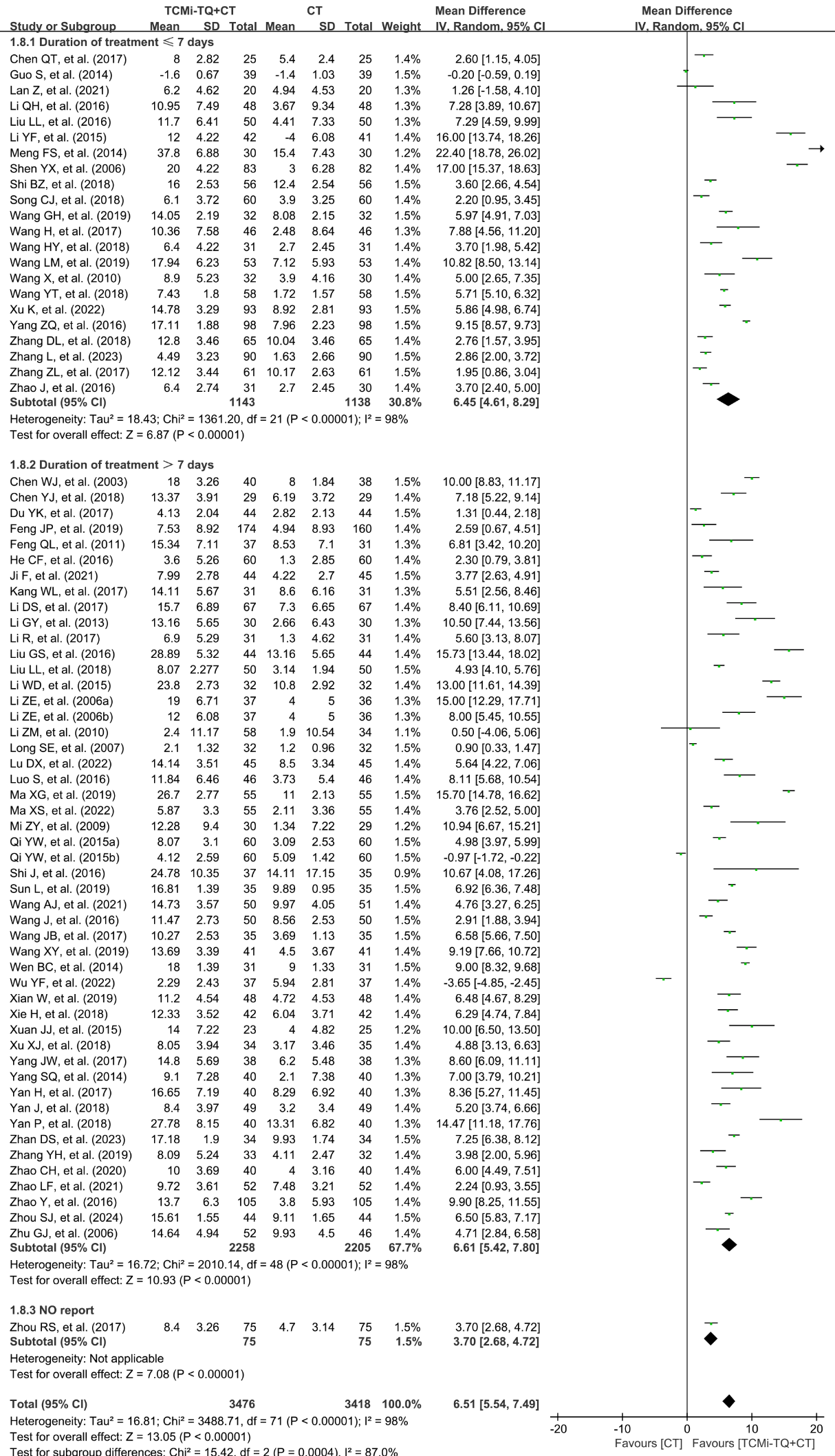
**Supplementary figure F4** Subgroup analysis of the impact of TCMi-TQ combined with conventional treatment on LVEF in AMI patients (based on the duration of treatment). Notes: CI, confidence interval; TCMi-TQ, Traditional Chinese medicine injections for Tonifying Qi; CT, conventional treatment; SGMI, Shengmai injection; SMI, Shenmai injection; SFI, Shenfu injection; AI, Astragalus injection.

**Supplementary**
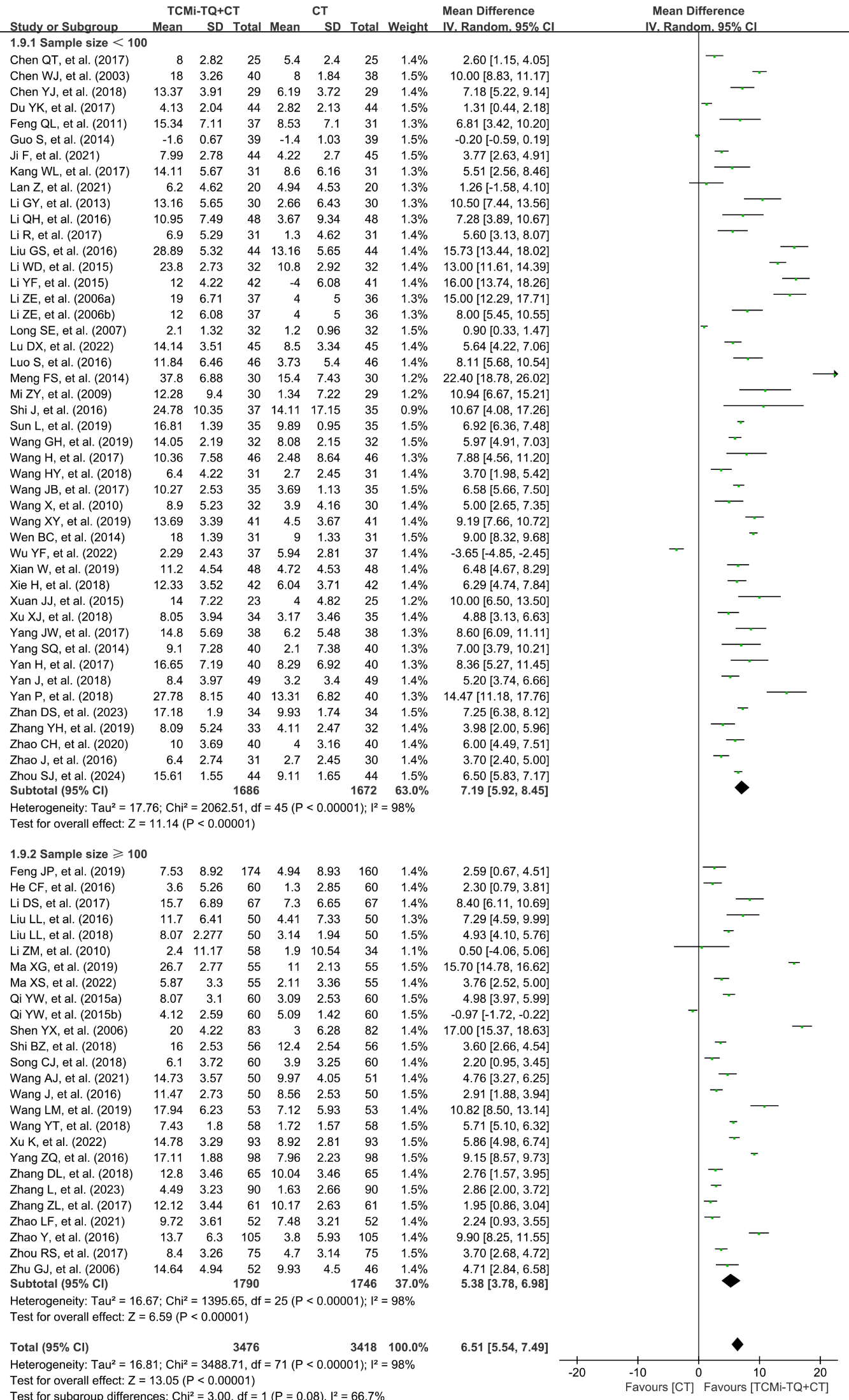
**figure F5** Subgroup analysis of the impact of TCMi-TQ combined with conventional treatment on LVEF in AMI patients (based on the sample size). Notes: CI, confidence interval; TCMi-TQ, Traditional Chinese medicine injections for Tonifying Qi; CT, conventional treatment.


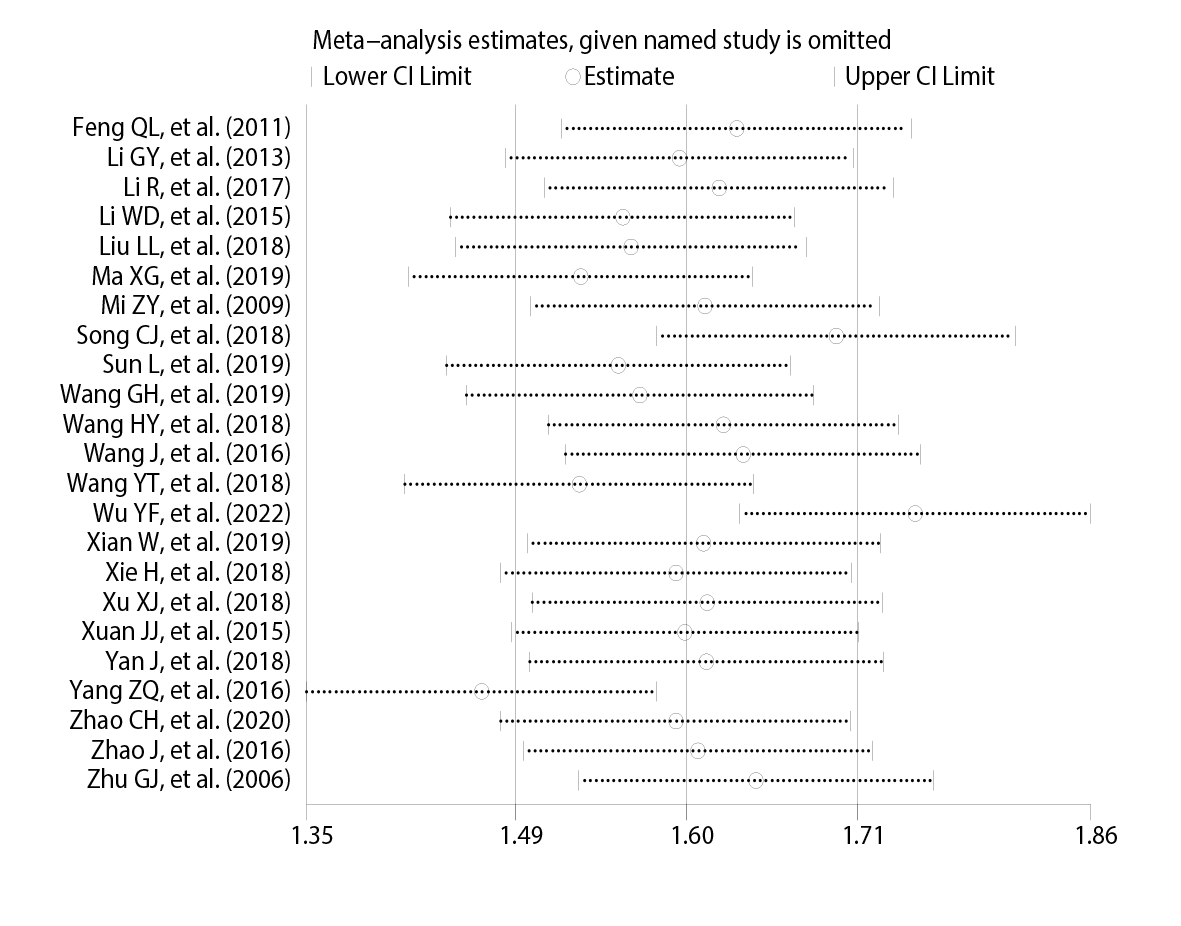

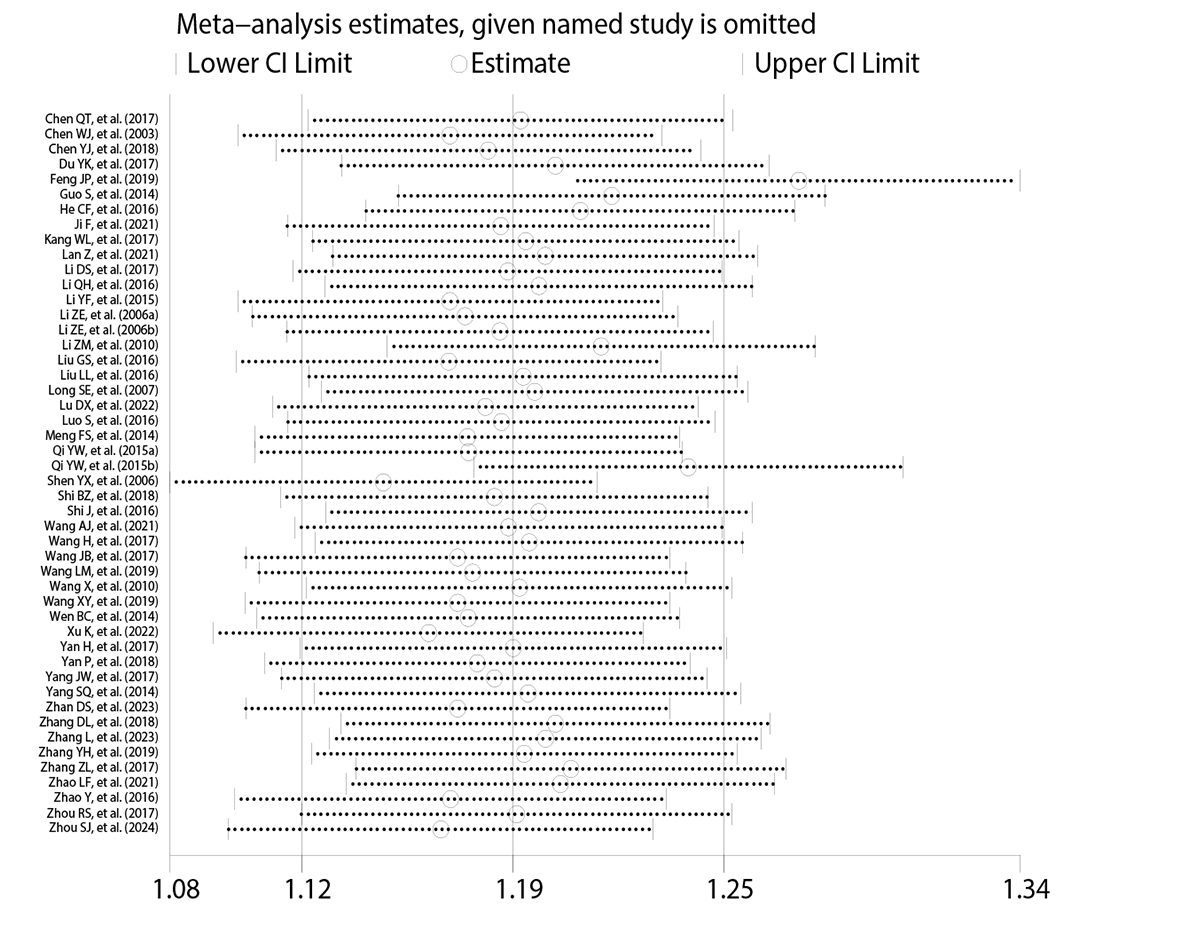
**Supplementary figure F6** Sensitivity analysis of the impact of TCMi-TQ combined with conventional treatment on LVEF in STEMI patient. Notes: CI, confidence interval.

**Supplementary figure F7** Sensitivity analysis of the impact of TCMi-TQ combined with conventional treatment on LVEF in ALL AMI patient. Notes: CI, confidence interval.

**
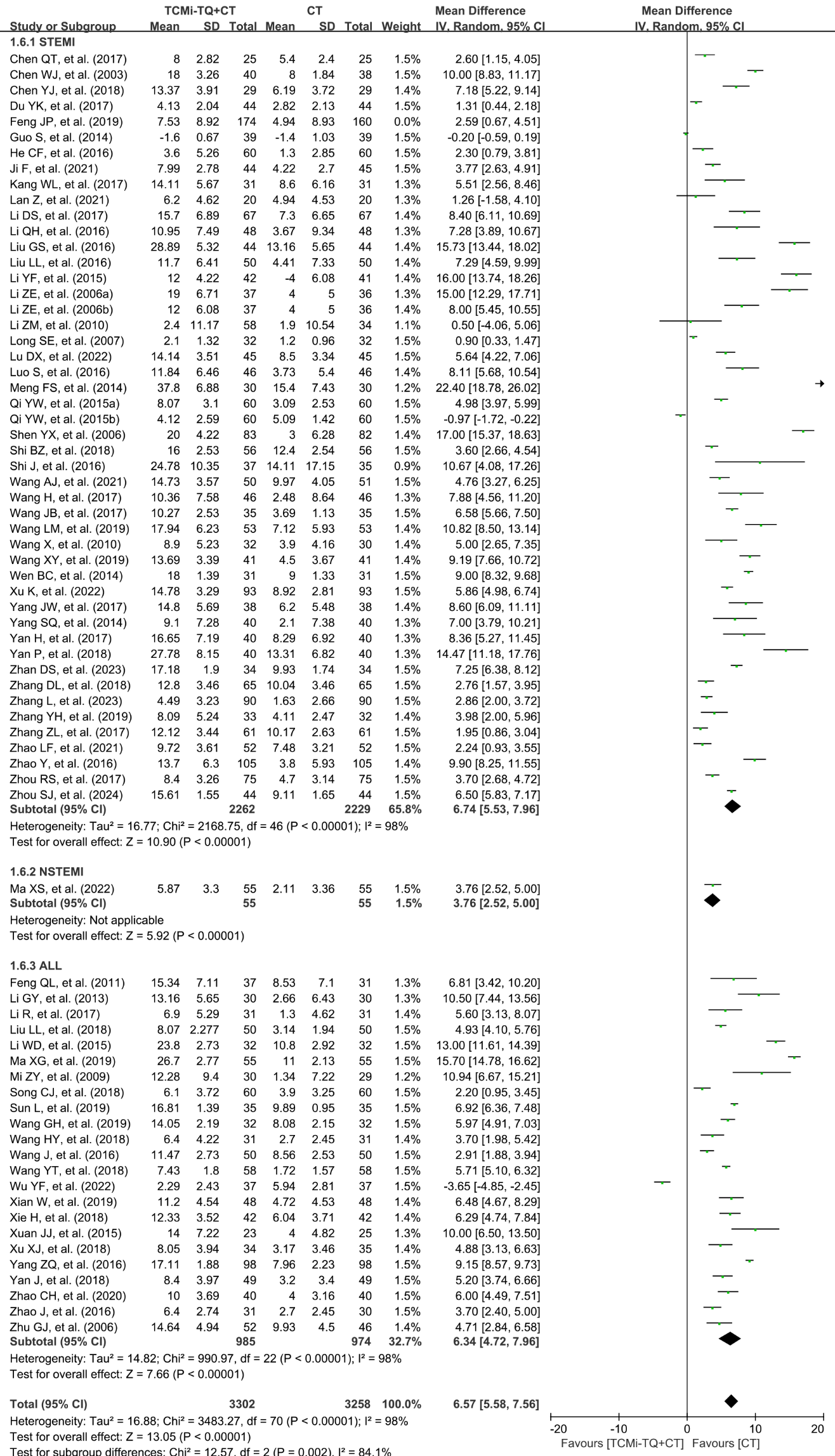
**

**Supplementary figure F8** Forest plot of the effect of TCMi-TQ combined with conventional biomedicine treatment on LVEF in patients with AMI (Excluding Feng JP et al., 2019). Notes: CI, confidence interval; MD, mean difference; TCMi-TQ, Traditional Chinese medicine injections for Tonifying Qi; CT, conventional treatment; AMI, acute myocardial infarction; STEMI, ST-segment elevation myocardial infarction; NSTEMI, non-ST-segment elevation myocardial infarction; LVEF, left ventricular ejection fraction.


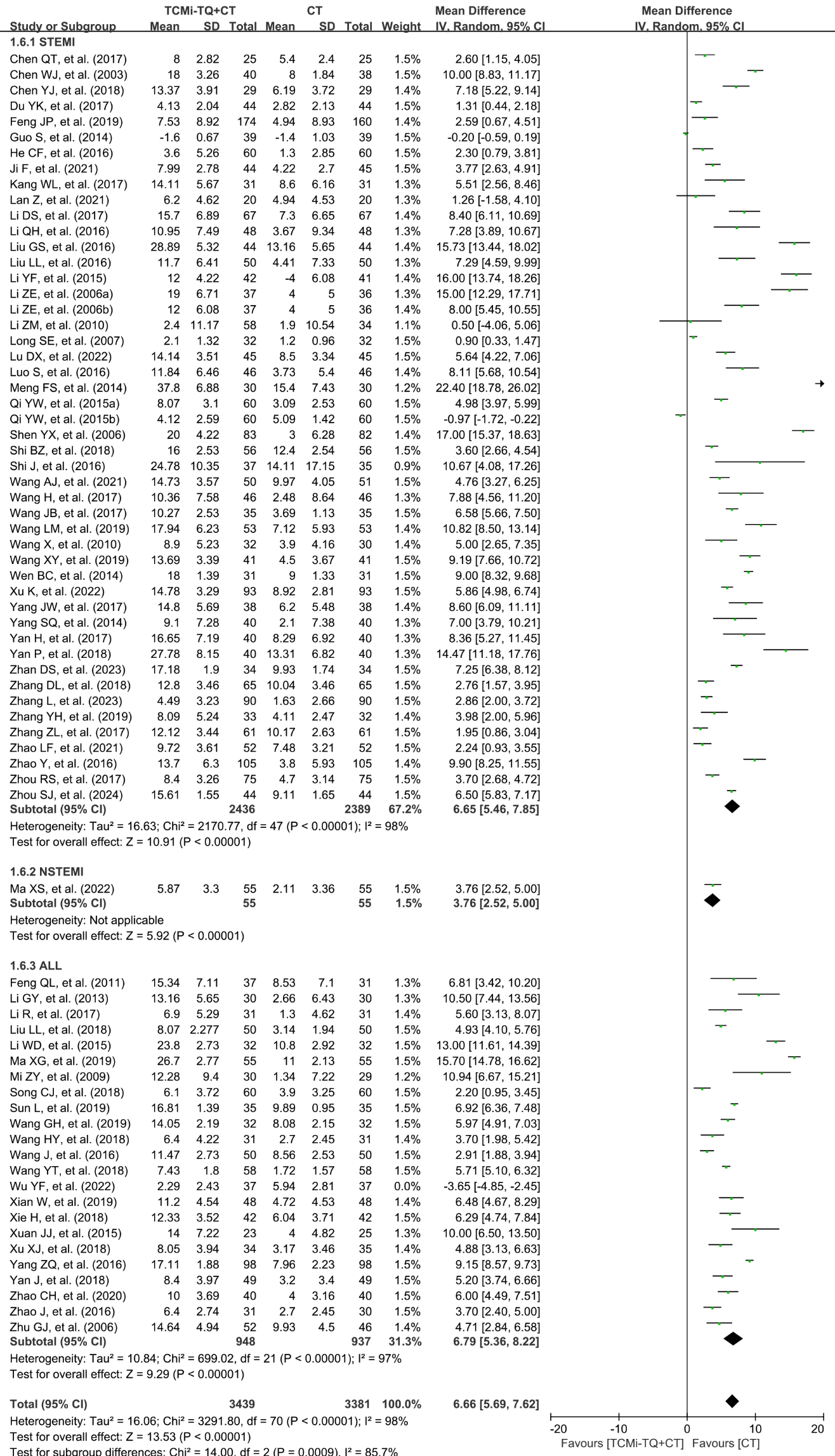


**Supplementary figure F9** Forest plot of the effect of TCMi-TQ combined with conventional biomedicine treatment on LVEF in patients with AMI (Excluding Wu YF et al., 2022). Notes: CI, confidence interval; MD, mean difference; TCMi-TQ, Traditional Chinese medicine injections for Tonifying Qi; CT, conventional treatment; AMI, acute myocardial infarction; STEMI, ST-segment elevation myocardial infarction; NSTEMI, non-ST-segment elevation myocardial infarction; LVEF, left ventricular ejection fraction.


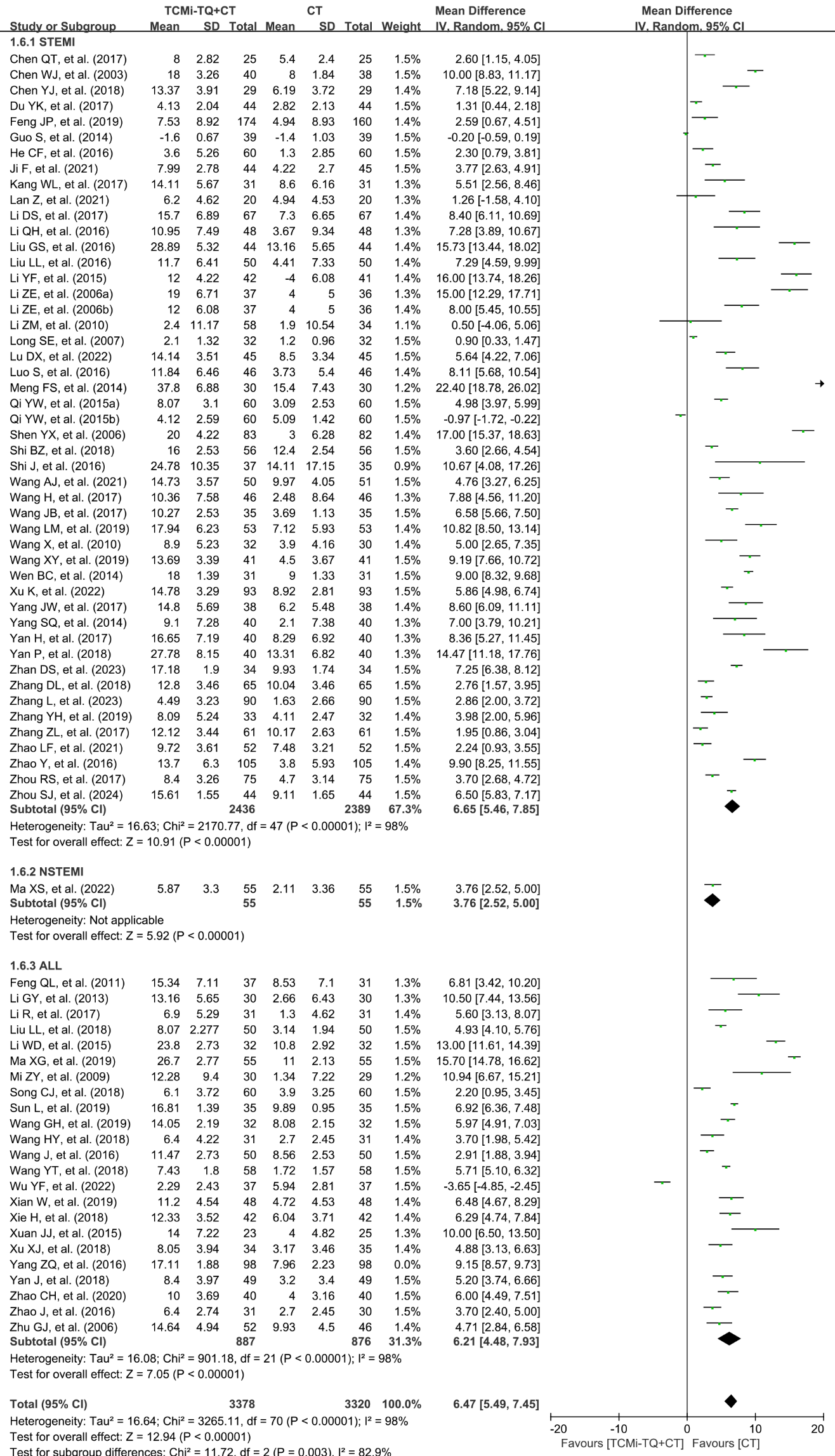


**Supplementary figure F10** Forest plot of the effect of TCMi-TQ combined with conventional biomedicine treatment on LVEF in patients with AMI (Excluding Yang ZQ et al., 2016). Notes: CI, confidence interval; MD, mean difference; TCMi-TQ, Traditional Chinese medicine injections for Tonifying Qi; CT, conventional treatment; AMI, acute myocardial infarction; STEMI, ST-segment elevation myocardial infarction; NSTEMI, non-ST-segment elevation myocardial infarction; LVEF, left ventricular ejection fraction.
